# The Glial Regenerative Response to Central Nervous System Injury Is Enabled by Pros-Notch and Pros-NFκB Feedback

**DOI:** 10.1371/journal.pbio.1001133

**Published:** 2011-08-30

**Authors:** Kentaro Kato, Manuel G. Forero, Janine C. Fenton, Alicia Hidalgo

**Affiliations:** NeuroDevelopment Group, School of Biosciences, University of Birmingham, Birmingham, United Kingdom; Stanford University School of Medicine, United States of America

## Abstract

Organisms are structurally robust, as cells accommodate changes preserving structural integrity and function. The molecular mechanisms underlying structural robustness and plasticity are poorly understood, but can be investigated by probing how cells respond to injury. Injury to the CNS induces proliferation of enwrapping glia, leading to axonal re-enwrapment and partial functional recovery. This glial regenerative response is found across species, and may reflect a common underlying genetic mechanism. Here, we show that injury to the Drosophila larval CNS induces glial proliferation, and we uncover a gene network controlling this response. It consists of the mutual maintenance between the cell cycle inhibitor Prospero (Pros) and the cell cycle activators Notch and NFκB. Together they maintain glia in the brink of dividing, they enable glial proliferation following injury, and subsequently they exert negative feedback on cell division restoring cell cycle arrest. Pros also promotes glial differentiation, resolving vacuolization, enabling debris clearance and axonal enwrapment. Disruption of this gene network prevents repair and induces tumourigenesis. Using wound area measurements across genotypes and time-lapse recordings we show that when glial proliferation and glial differentiation are abolished, both the size of the glial wound and neuropile vacuolization increase. When glial proliferation and differentiation are enabled, glial wound size decreases and injury-induced apoptosis and vacuolization are prevented. The uncovered gene network promotes regeneration of the glial lesion and neuropile repair. In the unharmed animal, it is most likely a homeostatic mechanism for structural robustness. This gene network may be of relevance to mammalian glia to promote repair upon CNS injury or disease.

## Abstract 2

A gene network involving Notch and Pros underlies the glial regenerative response to injury in the *Drosophila* central nervous system.

## Author Summary

The process of tissue regeneration has long been studied as a route to understanding what promotes structural robustness of cellular networks in animals. In the central nervous system (CNS), neurons and glia interact throughout adult life and during learning, at the same time accommodating functional changes while preserving the structural integrity necessary for function. The mechanisms that confer this combination of structural robustness and functional plasticity in the CNS are unknown, but they may be shared with the cellular responses to injury, which also require structural changes while retaining function. The glial cells that enwrap axons respond to injury by dividing and re-enwrapping them, leading to partial recovery of function. Here, we use *Drosophila* genetics to uncover a gene network underlying this glial regenerative response. This gene network enables glia to divide upon injury, prevent uncontrolled proliferation, and differentiate. We find that the network also has homeostatic properties: two cell-cycle activators (Notch and NFκB) promote the expression of a cell cycle inhibitor (Pros), providing negative feedback on cell division. Pros is also essential for glial differentiation, enabling the clearance of cellular debris and axonal enwrapment, and priming glia for further responses. By removing these genes or adding them in excess, we can shift the response to injury from prevention to promotion of lesion repair. This gene network is thus a homeostatic mechanism for structural robustness. Our findings from *Drosophila* may also help manipulation of glia to repair the damaged human CNS.

## Introduction

The structure of organisms is robust. Cells accommodate changes in their environment during development and throughout life by adjusting cell number and cell morphology to preserve overall organismal integrity. In the central nervous system (CNS), adjustments are carried out in interacting populations of neurons and glial cells, from development to learning, ultimately enabling function. Injury and regeneration experiments and theoretical models have long been used to uncover the cellular and molecular mechanisms of how cells “sense” and maintain normal organ structure [1]. The premise is that shared mechanisms may underlie normal structural homeostasis and plasticity, and cellular responses to injury. Understanding such mechanisms is one of the frontiers in biology. It will also lead to a greater understanding of regeneration and repair of relevance to the treatment of human injury and disease. The fruitfly Drosophila is an ideal model organism for discovering gene networks, and has been successfully used to investigate cellular responses to CNS injury [2]–[6]. Here, we use Drosophila to uncover a gene network that controls the glial regenerative response to injury and promotes robustness in the normal CNS.

Previous experiments had revealed two important findings about glial responses to injury in Drosophila. Firstly, enwrapping glial cells become phagocytic upon injury clearing cellular debris. This phagocytic function requires the corpse engulfment receptor Draper, which is specifically expressed in enwrapping glia, and whose function involves Simu, Src42A, and Shark [4],[6]–[9]. Secondly, stabbing injury in the adult head [5] and neuronal ablation in the embryonic Ventral Nerve Cord (VNC) [10] induce the proliferation of glial cells (including the enwrapping Longitudinal Glia). Similar findings had long before been observed in the cockroach [11]–[13]: surgical lesioning and chemical ablation of enwrapping glial cells induced cell transformation leading to the phagocytosis of cellular debris, and most remarkably glial proliferation. This restored glial numbers, enwrapment, and normal electrophysiological function.

Insect glia may be evolutionarily distant from mammalian glia, but it can be insightful to compare them. Injury induces distinct responses in mammalian CNS glial cells. Astrocytes normally maintain ionic homeostasis, provide nutrients, and participate in synapses. Microglia are the immune cells of the brain, and are normally in a resting state. Upon injury, microglia and astrocytes phagocytose degenerating axons and other cellular debris, and they can also form a glial scar that inhibits axonal regeneration [14]. Ensheathing glia (oligodendrocytes) normally myelinate axons for saltatory conduction in vertebrates, maintain ionic homeostasis, and provide metabolic and trophic support to axons [15],[16]. Oligodendrocyte progenitor cells (OPCs) respond to injury by dividing, and resulting oligodendrocytes remyelinate [17]–[19]. This latter response is regenerative, leading to spontaneous re-enwrapment of axons and partial functional recovery, for instance of locomotion [20]–[22]. Conditions such as spinal cord injury, stroke, and multiple sclerosis induce the proliferation of OPCs resulting in spontaneous remyelination of CNS axons, and underlie the “remission” phases of multiple sclerosis [18],[23]–[28].

Thus, the regenerative response of ensheathing glia occurs across the animals, from insects [11] to fish [23] and humans [20]. In cockroach, fruitflies, and vertebrates, ensheathing glia proliferate upon injury, and both in insects and mammals this response can lead to limited remyelination and some recovery of function. This reveals that there is an endogenous tendency of the CNS to repair itself. Its manifestation across species may reflect a common underlying gene network. If understood, it could be harnessed to stimulate CNS repair. Here, we search for a Glial Regenerative Response (GRR) gene network that can promote repair after injury and confer structural robustness in the normal animal.

The following factors are promising candidates to belong to this gene network. The Drosophila TNF super-family member Eiger triggers the proliferation of adult brain glia upon injury in fruitflies [5]. TNFα also triggers the proliferation of mammalian oligodendrocytes progenitors through its receptor TNFR2 upon injury [29]. While in other contexts TNFR2 is thought to function by activating NFκB [30] (which can promote the cell cycle), whether this is the case for CNS glial cells and whether this activates glial proliferation are unknown.

Notch maintains the undifferentiated and stem cell state in many contexts [31]. In Drosophila, Notch maintains the mitotic potential of embryonic ensheathing glia in interaction with the Jagged1 homologue, Serrate, from axons [10],[32]. Similarly, in vertebrates, Notch1 maintains the oligodendrocyte progenitor state by interacting with its ligand Jagged1 present in axons [33]. However, the functions of Notch in the glial regenerative response remain unsolved. Notch1 is present in adult NG2+ OPCs, and it is upregulated upon injury and during regeneration, but conditional Notch1 knock-out in OPCs does not prevent the regenerative response [34],[35]. Notch1 can inhibit the differentiation of progenitors into myelinating oligodendrocytes, preventing repair [33],[36], but presence of Notch1 signaling in these cells does not prevent the regenerative response either [34],[35]. Finding out how to control Notch1 function, to enable glial proliferation, and to subsequently promote ensheathing glial differentiation is thus a key issue.

The transcription factor Prospero (Pros) interacts with Notch in ensheathing glia in Drosophila embryos [10], but how Pros and Notch affect each other's function is not understood. Pros appears to have opposite functions in neuroblasts and in glia. In neuroblasts Pros is a tumour suppressor, as mutations in *pros* result in over-proliferation [37]–[40]. Instead in glia both Pros and Notch are necessary for proliferation, and mutations in *pros* do not result in glial hyperplasia [10],[32]. Unravelling the relationship between Notch and Pros may hold the key to understanding how glial proliferation and differentiation are regulated.

Here, we uncover the Glial Regenerative Response gene network in Drosophila. For this, we establish a new CNS injury paradigm in the larval VNC. We use the larva because it is more accessible than the adult while it has locomotion, senses, learning, and memory, enabling the investigation of repair in the context of a fully functional CNS. We show that the ensheathing Interface Glia of the VNC respond to injury by phagocytosing and clearing cellular debris and by dividing. We reveal a gene network that controls the balance of glial proliferation and differentiation, and it is comprised of two feedback loops: one involving Pros and Notch, and a second involving Pros and Dorsal/NFκB, connected via Eiger/TNF and Wgn/TNFR signaling. By manipulating this gene network we could shift from exacerbating the damage to promoting repair of the damaged neuropile. The uncovered gene network is a homeostatic mechanism for structural robustness and plasticity.

## Results

### Characterisation of Glial Responses to Stabbing Injury in the Drosophila Larval CNS

**Figure 1:**
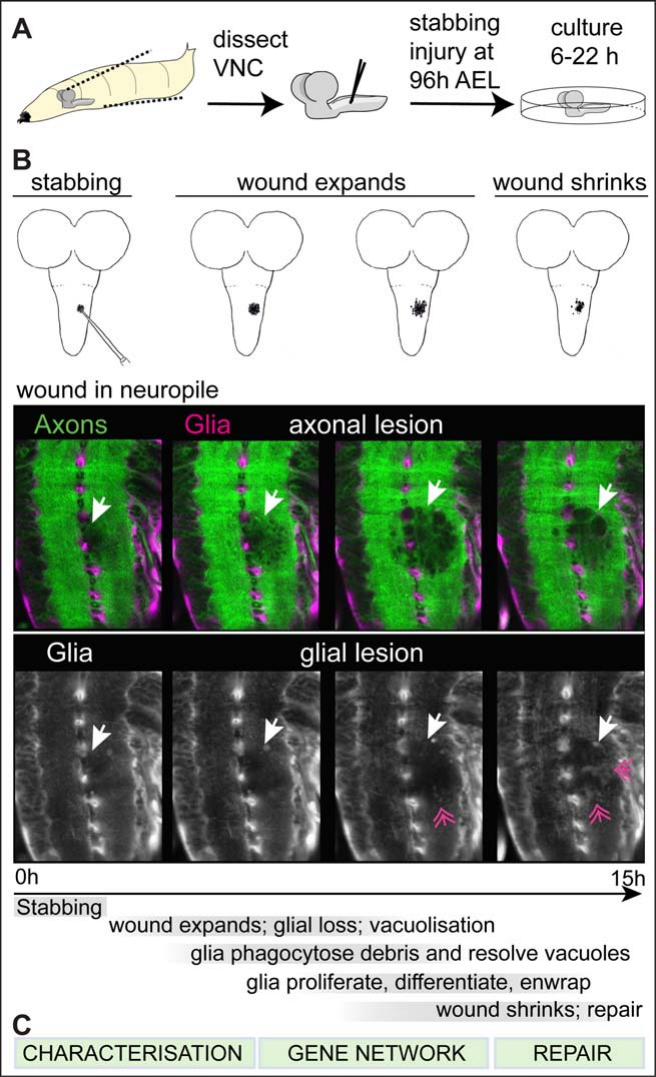
Stabbing injury in the Drosophila larval ventral nerve cord. (A) The ventral nerve cord (VNC) is dissected out of the larva, stabbed with a tunsgeten needle, and cultured in a well-plate, after which it is fixed and processed for immunohistochemistry, or it is filmed. (B) Progression of the wound over time: the drawings show longitudinal views of the VNC plus optic lobes to illustrate that in normal larvae, the wound initially expands and subsequently shrinks. The confocal images are snapshots of a time-lapse movie of a VNC bearing *repoGAL4>UASdsRed* to visualise the glia, and the G9 exon-trap line expressing GFP in all axons. Both the axonal and the glial lesions repair naturally to some extent. Arrows indicate the wound, and feathered arrows indicate glial processes invading the lesion as the wound shrinks. (C) The next figures in the article show the characterisation of the glial responses to injury, the gene network underlying the glial regenerative response, and how altering the balance of gene functions in this network can shift from exacerbating the damage to promoting repair. Anterior is up. Genotype: *UASDsRed/+;G9/+;repoGAL4/+*.

To test how Drosophila larval glia respond to injury, dissected ventral nerve cords (VNCs) were stabbed with a fine needle and cultured (Figure 1A). Stabbing was applied dorsally into the neuropile, which comprises the bundles of CNS axons and Interface Glia (IG) [41]. To obtain an overview of the injury response, we performed a time-lapse analysis. Axons were visualised with GFP driven by the protein trap line G9, and glia with *repoGAL4>DsRed* (Figure 1B, Video S1 and Video S2). The wound initially expanded, and vacuoles formed within the neuropile. However, after 6 h of culture, glial processes invaded the wound, the axonal and glial wound began to shrink, and by 22 h the wound could heal considerably. This suggested that there is a natural mechanism that can promote repair. Here (Figure 1C), we (1) characterised the glial responses to injury, (2) investigated the gene network controlling the regenerative potential of glia, and (3) tested whether altering the functions of this gene network could promote glial regeneration and axonal repair.

**Figure 2:**
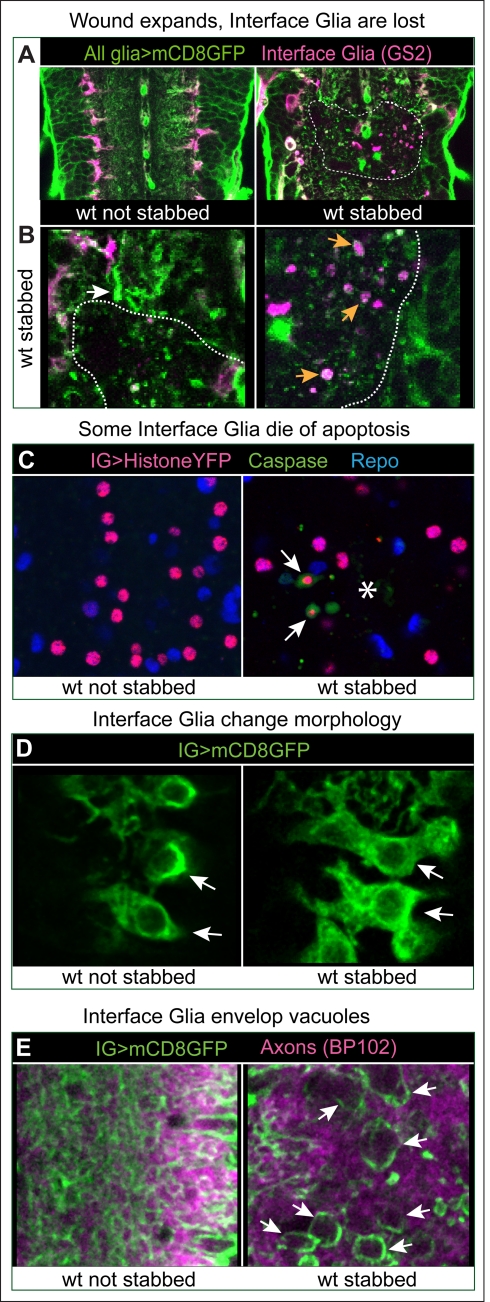
Glial responses to injury. (A) Injury affects most prominently the Interface Glia (magenta), visualised with GS2. Longitudinal views of the VNC, dashed lines delineate wound edges. (B) Higher magnification views of (A) showing loss of GS2+ glia upon injury, accumulation of glial material (white arrow), and GS2+ glial debris (orange arrows). (C) Some Interface Glia die of apoptosis, as the cytoplasmic apoptotic marker cleaved-Caspase-3 surrounds Interface Glial nuclei labelled with Repo and Histone-YFP expressed in IG only with *alrmGAL4* (arrows). Asterisk indicates lesion site. (D) Glia project more filopodia and lamellipodia (arrows) after injury compared to non-stabbed controls. Glial membranes are visualised with mCD8GFP and anti-GFP. (E) Interface Glial processes envelop vacuoles that form in the axonal neuropile after injury (arrows). All axons are labelled with BP102 antibodies. Anterior is up. Genotypes: (A,B) all glia: *w; UASmCD8GFP/+*;*repoGAL4/+*. (C,D,E) Interface Glia: (C) IG nuclei: *w;alrmGAL4/UAShistoneYFP*; (D,E) glial processes and membranes *w;alrmGAL4/UASmcD8GFP*.

To characterise the glial responses to injury, glial membranes were visualised with *repoGAL4>mCD8-GFP* and Interface Glia (IG) with anti-Glutamine Synthetase 2 (GS2) (Figure 2A,B and Figure S1). This revealed the stabbing wound dorsally and an indentation ventrally (Figure S1, Video S3 and Video S4). Although stabbing damaged to some extent surface and cortex glia (unpublished data), it affected most prominently the IG, causing GS2+ glial loss (Figure 2A,B and Figure S1) and GS2+ glial debris at 6 h after injury (Figure 2B). We used the *alrmGAL4* driver, which is restricted to the Pros+ IG (Figure S2A), to visualize IG nuclei with HistoneYFP, and this showed that some IG were lost through apoptosis (Figure 2C, intact VNCs had 0 cleaved-Caspase-3+ YFP+ IG *n* = 10 VNCs versus stabbed VNCs with an average of 1.5 cleaved-Caspase-3+ YFP+ in 57% of the VNCs *n* = 14). In remaining IG, injury provoked an increase in the size and complexity of cytoplasmic projections (seen with the membrane reporter *mCD8GFP*, Figure 2D). As observed in time-lapse, injury led to vacuolization of the neuropile, as holes formed within the axonal bundles (Figure 1B and Figure 2E). IG projections enveloped these vacuoles (Figure 2E).

**Figure 3:**
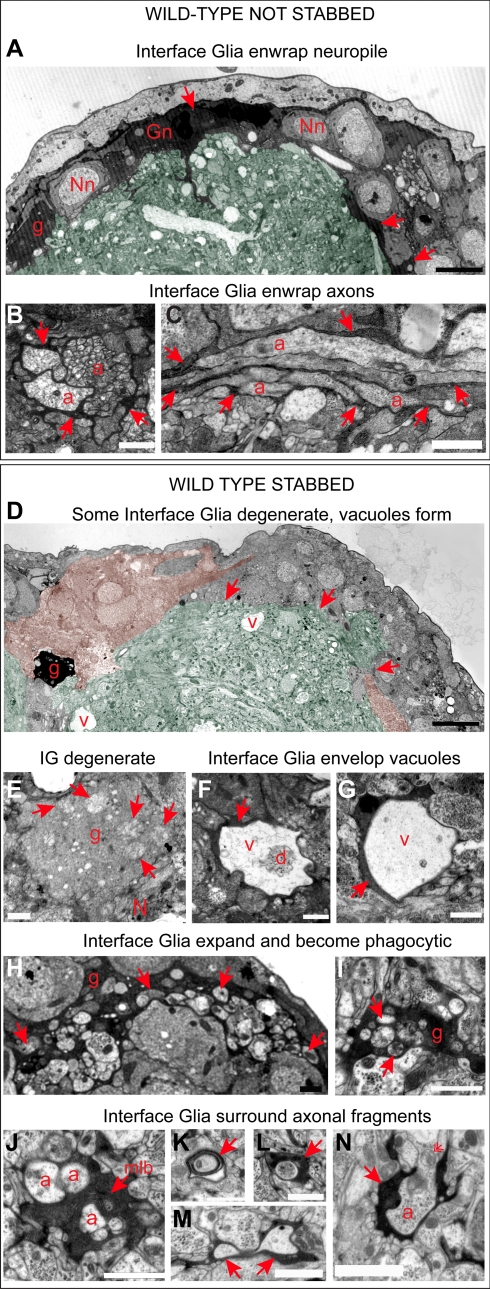
IG enwrap the neuropile and axons, and they are phegocytic upon injury. Transmission Electron Microscopy images showing that: (A,B,C) in wild-type non-stabbed VNCs, IG nuclei overlie the neuropile and their cytoplasmic projections enwrap the neuropile (A, arrows; in normal, not stabbed speciments IG processes are dark grey/black). IG also send processes into the neuropile where they can enwrap small axonal bundles (B, arrows) or individual axons (C, arrows). (D) Upon injury, IG are depleted and enwrapment of the neuropile breaks down (arrows). Only a small black glial cell can be seen here (g, compare with A). There are degenerating cells surrounding the neuropile, identified by their swollen mitochondria (colour coded filled-in pink; green colouring indicates the neuropile in A as well as D). Large vacuoles (v) form in stabbed VNCs. (E) A degenerating glial cell with multiple swollen mitochondria (arrows). (F,G) Processes of remaining IG envelop vacuoles (arrows, note degenerating axon within vacuole in F). (H,I) IG are phagocytic and their processes expand outside (H) and within (I) the neuropile, engulfing cell debris (arrrows point to phagosomes). (J) IG with multi-lamellar bodies (mlb), a hallmark of phagocytosed degenerating cells. (K,L,M) IG processes (arrows) surround isolated axons or axonal fragments. (N) IG processes surround a degenerating axon, identified by its swollen mitochondria (feathered arrow). In TEM axons appear off-white and granular, glial cytoplasms are dark grey or black. (A,B,D,H) are transverse sections, dorsal is up; (C) is a longitudinal view of the axons. Gn, Interface Glia nucleus; Nn, neuronal nucleus; g, glial process; a, axon; N, neuropile. Scale bars: (A,D) 5 microns; rest: 1 micron.

To further characterise these aspects, we used Transmission Electron Microscopy (TEM). In wild-type wandering larvae, the IG nuclei were located outside the neuropile and their cytoplasms enwrapped the entire neuropile (Figure 3A). IG processes projected into the neuropile, where they could enwrap smaller axonal bundles (Figure 3B) and individual axons (Figure 3C). As seen with confocal microscopy, TEM confirmed that injury caused glial loss with breakdown of neuropile enwrapment after 6 h (Figure 3D), and vacuoles formed within the neuropile (Figure 3D). Some Interface Glia degenerated via necrosis as seen by swelling of mitochondria (Figure 3E). Remaining IG expanded their cytoplasmic projections both around and within the neuropile (Figure 3F–J), which was never observed in intact specimens. IG processes lined vacuoles (Figure 3F,G), phagocytosed axonal fragments and other cellular debris, as revealed by phagosomes and multilamellar bodies within the glial processes (Figure 3H–J). IG processes frequently wrapped around isolated axons that could be degenerating (Figure 3K–N). These data show that upon injury IG phagocytose cellular debris, presumably clearing the lesion. Altogether, these data demonstrate that larval IG enwrap CNS axons, and that they are damaged by, and respond to, injury.

### Characterisation: Stabbing Induces a Glial Proliferative Response

**Figure 4:**
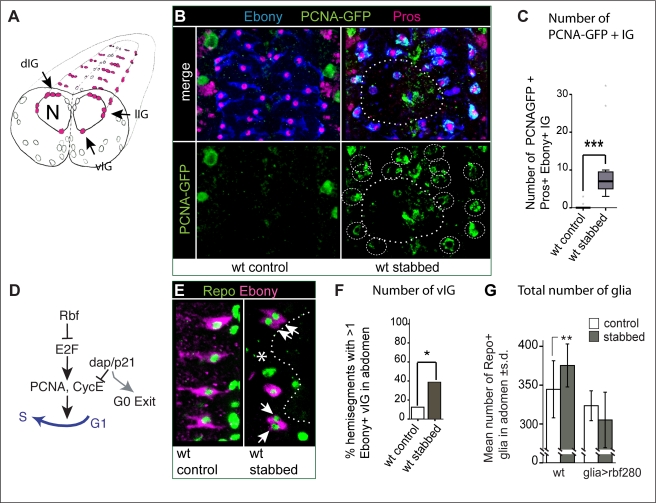
Stabbing induces an increase in glial proliferation. (A) Illustration of a transverse view through the larval VNC, showing the IG (pink, arrows) enwrapping the neuropile (N): dIG, dorsal; lIG: lateral; and vIG: ventral Interface Glia. (B) Stabbing injury induces the local expression of the *PCNA-GFP* reporter in Ebony+ and Pros+ IG (circled) 4 h after stabbing; (C) quantification in boxplots around the median. (D) Illustration of how PCNA and other cell cycle regulators control the G1/S transition. (E) Stabbing injury induces the local increase in Ebony+ and Pros+ ventral IG (vIG, arrows), (F) quantification. (G) The number of abdominal Repo+ glial cells, counted automatically with DeadEasy Glia software, increases in wild-type upon stabbing; expression of constitutively active Rbf in glia abolishes this response, demonstrating that the increase in glial number is due to cell proliferation. Numbers above bars indicate sample size *n* = number of VNCs; error bars in (G) are standard deviation (s.d.). * *p*<0.05,** *p*<0.01, *** *p*<0.001. Dashed lines delimit injury (and PCNA-GFP positives cells in B). Sample sizes, statistical tests, and *p* values are given in Table S1. Genotypes: (B,C) *PCNA-GFP*; (E,F) *yw*; (G) *yw* and *w*; *repoGAL4/UASRbf280*.

Next, we investigate if stabbing induced IG proliferation. Based on their location, the IG are classified into dorsal (dIG), lateral (lIG), and ventral (vIG) IG (Figure 4A). They are identified by co-localisation of the pan-glial marker Repo and the transcription factor Pros in nuclei, surrounded by the cytoplasmic marker Ebony (Figure S2B,C). IG do not normally divide [42], but are arrested in G1 and have mitotic potential (Text S1 and Figure S3). To examine cell proliferation, we used PCNA-GFP, a reporter with E2F binding sites that reveals GFP expression when cells go through S-phase (Figure 4B,C,D) [43]. PCNA-GFP+ Pros+ Ebony+ IG were rarely seen in non-stabbed controls, but stabbing increased their frequency at the lesion site (Figure 4B) and throughout the neuropile (Figure 4C). We did not find any Ebony-negative IG with PCNA-GFP (unpublished data), suggesting that Ebony+ Pros+ IG are the only IG that divide in response to injury. Normally there is one Ebony+ vIG per hemisegment, but the number of Ebony+ vIG adjacent to the wound increased significantly in stabbed larvae (Figure 4E,F). Altogether these data show that stabbing causes a local increase in proliferation of Pros+ Ebony+ IG at the lesion site.

A BrdU pulse experiment (a commonly used method to visualise proliferating cells) also revealed an overall increase in the number of dividing IG upon stabbing, to 50% of Ebony+ IG being also BrdU+ (*p*<0.05), comprising local IG at the lesions site (Figure S4) and at some distance along the VNC. This suggested that neuronal damage may also affect glial cells at a distance from the original lesion, which is explained as axons extend along the whole length of the VNC. Stabbing may also affect other glial classes than IG. To take these facts into account, we purposely developed DeadEasy Glia software to automatically count in vivo all Repo+ glial cells (Figure 4G and Figure S5). After 22 h culture, stabbed VNCs had more glial cells than non-stabbed controls (Figure 4G). This effect was abolished when a cell cycle inhibitor—constitutively active *Retinoblastoma protein factor (Rbf280/Rb)*—was expressed in glia (Figure 4G). This demonstrates that the increase in glial number upon stabbing was due to the induction of glial proliferation. These data show that stabbing the larval VNC causes an increase in glial proliferation and a consequent increase in glial cell number. Although we cannot rule out that other glial cells might also divide, our data demonstrate that this response involves the IG.

### The Gene Network: The Glial Proliferative Response to Stabbing Injury Depends on Pros, Notch, Eiger/TNF, Wgn/TNFR, and Dorsal/NFκB

**Figure 5:**
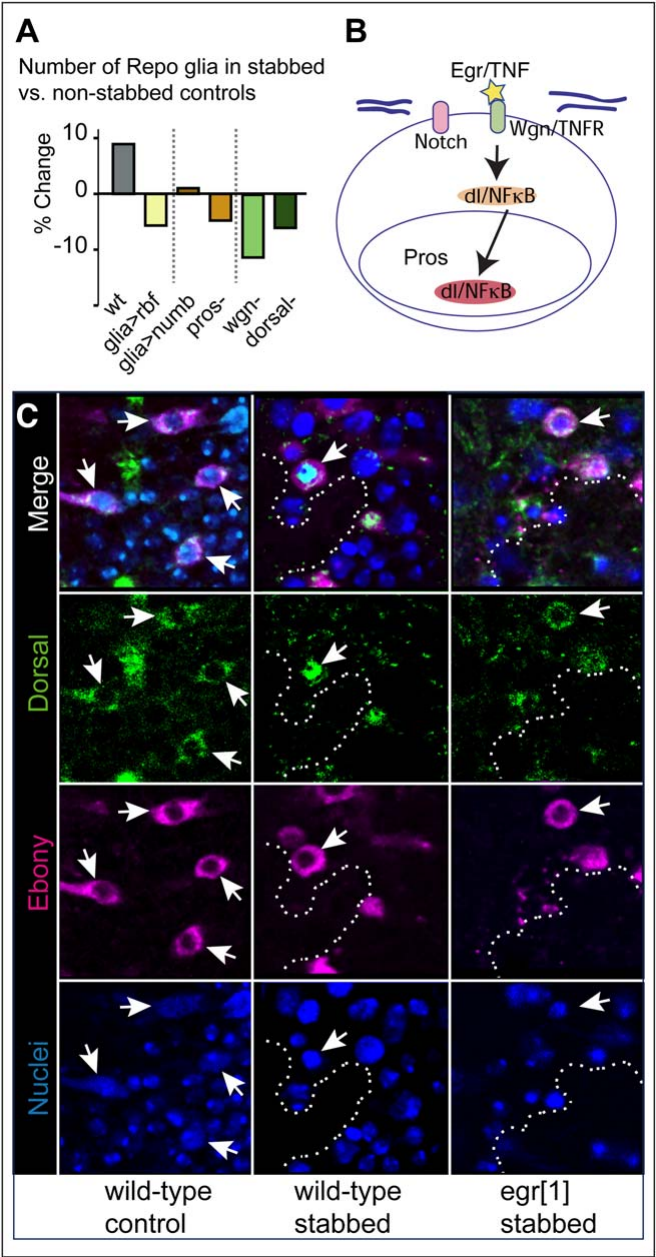
The glial regenerative response requires Pros, Notch, Egr/TNF, Wgn/TNFR, and Dorsal/NFκB. (A) Bars represent percentage change in the number of Repo+ glia in stabbed abdominal VNCs normalised over non-stabbed controls of the respective genotypes. The glial proliferative response to injury is abolished in larvae expressing *Rbf* or *numb* in glia, and in *pros*, *wgn* and *dorsal* mutant larvae. (B) Diagram showing that stabbing injury induces Egr/TNF, which binds Wgn/TNFR and activates Dorsal/NFκB, which is translocated to the nucleus, where it functions as a transcription factor. (C) In the wild-type non-stabbed control VNC, Dorsal is cytoplasmic in Ebony+ IG (arrows). Intense nuclear Dorsal is observed in stabbed wild-type (arrows), but not in *egr^1^* mutant larvae, where Dorsal remains cytoplasmic (arrows). Longitudinal sections. Nuclear dye is Hoechst33342. Dashed lines indicate lesion boundaries. Sample sizes, statistical tests, and *p* values are given in Table S1. Genotypes from left to right: (A) (1) *yw*; (2) *repoGAL4/UASrbf280*; (3) *repoGAL4/UASnumb*; (4) *pros^voila1^/pros^S044116^*; (5) *Df(1)Exel7463/wgn^e00637^*; and (6) *dorsal^1^/dorsal^H^*. (C) (1,2) *yw*; (3) *egr^1^*.

We next asked what genes might control the proliferative glial response to injury. Notch and Pros regulate the mitotic potential of embryonic glia [10]; thus, we wondered if they might be involved. *pros^voila1^/pros^S044116^* hypomorphic mutants specifically affected larvae, since embryogenesis proceeded normally but the levels of Pros dropped in IG by the third instar larval stage (Figure S6A,B). In *pros^voila1^/pros^S044116^* VNCs, Ebony was downregulated, meaning that Ebony is a downstream target of Pros (Figure S6B), but there were no major developmental defects as Repo and GS2 expression were normal (Figure S6C,D). Expression of the Notch antagonist *numb* with *repoGAL4* to knockdown Notch specifically in glia did not cause general developmental defects either (see below). However, the glial proliferative response to injury was significantly reduced both upon the glial over-expression of *numb* (Figure 5A) and in *pros^voila1^/pros^S044116^* mutant larvae (Figure 5A). In particular, IG number decreased upon stabbing in *pros^S044116^* mutant larvae (Figure S7 *p*<0.01). These data show that Notch and Pros are required for the glial proliferative response.

Drosophila Egr/TNF is required for glial proliferation in response to injury in the adult brain [5], but how it implements this is unknown. In mammals, TNF can induce cell proliferation via the activation of NFkB [30], but whether it does in glial progenitors upon CNS injury is unknown. Thus we asked whether in our injury paradigm, IG proliferation required Egr/TNF, Wengen (Wgn)/TNFR, and Drosophila NFκB, Dorsal. *egr/TNF* and *wgn/TNFR* are expressed in the VNC (Figure S8A–D) and Dorsal/NFkB is distributed preferentially in Ebony+ *pros-lacZ+* IG (Figure S8E). There were no major developmental defects in the VNC of *dorsal^H^/dorsal^1^* or *egr^1^/egr^3^* mutant larvae (Figure S9). However, the glial proliferative response was abolished in stabbed *egr^1^* (unpublished data), *wgn^e00637^/Df(1)Exel7463*, and *dorsal^1^/dorsal^H^* mutant larval VNCs (Figure 5A). These data suggest that Wgn/TNFR, its ligand Egr/TNF, and Dorsal/NFκB are required for the glial proliferative response to injury.

To verify this, we asked whether stabbing resulted in the activation of Dorsal/NFκB in glia. In its inactive form, Dorsal/NFκB is cytoplasmic, and upon signaling it is translocated to the nucleus to function as a transcription factor. We found intense nuclear distribution of Dorsal/NFκB in IG upon injury in wild-type (Figure 5C *p*<0.05), but not in *egr^1^* mutants (Figure 5C). This shows that stabbing induces the activation of Dorsal/NFκB in IG, which depends on Egr/TNF (Figure 5B).

Therefore, we sought to find out how might Pros, Notch, Eiger/TNF, and Dorsal/NFκB implement their functions in the glial proliferative response to injury.

### The Gene Network: Pros-Notch Feedback Keeps Glial Cells on the Brink of Dividing

**Figure 6:**
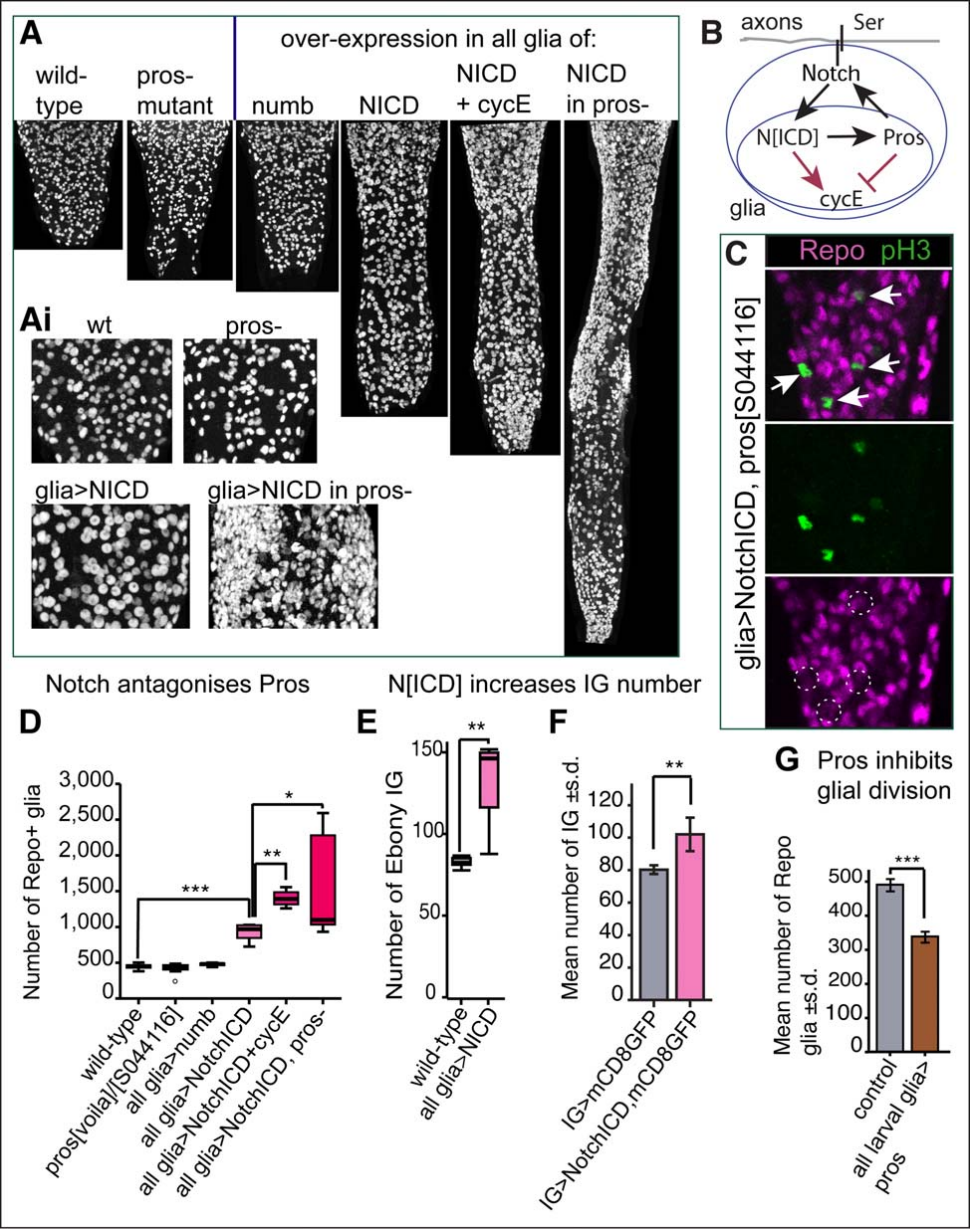
Pros-Notch feedback maintains glial cells on the brink of dividing. (A,Ai,D) The number of glial cells in *pros* mutants or upon over-expression of the Notch antagonist *numb* in glia was indistinguishible from wild-type at the wandering stage. However, the number of glial cells increased upon over-expression of *Notch^ICD^* in glia alone, further together with over-expression of *cycE*, and further still in a *pros* mutant background. (A) Longitudinal projections of abdominal VNCs labelled with anti-Repo at the wandering stage; (Ai) higher magnification of (A), except for *glia>Notch^ICD^* in *pros*, where a different specimen was used. (B) Diagram illustrating that Notch and Pros have antagonistic functions on the cell cycle and maintain each other through positive feedback, thus keeping glial cells on the brink of dividing. (C) Only glial divisions (Repo+ pH3+, arrows and dotted circles) are detected in the abdomen of *pros^S044116^repoGAL4/pros^S044116^UASNotch^ICD^myc* larvae. (E) Over-expression of *Notch^ICD^* in glia causes an increase in Ebony+ IG number. (F) Over-expression of *Notch^ICD^* in IG causes an increase in IG number. (G) Over-expression of *pros* in larval glia reduces glial number at the wandering stage, showing that *pros* inhibits cell division. (D,E) are box-plots; in (F,G) error bars are standard deviation (s.d.); * *p*<0.05; ** *p*<0.01; *** *p*<0.001. Sample sizes, statistical tests, and *p* values are given in Table S1. Genotypes from left to right: (A,Ai,D) (1) *yw*, (2) *pros^voila1/^/pros^S044116^*, (3) *w;;repoGAL4/UASnumb*, (4) w;;*repoGAL4/UASNotch^ICD^myc*, (5) *w;;repoGAL4/UASNotch^ICD^myc UAScycE*, and (6) *pros^S044116^repoGAL4/pros^S044116^UASNotch^ICD^myc*. (C) *pros^S044116^repoGAL4/pros^S044116^UASNotch^ICD^myc*. (E) (1) *yw*, (2) *w;;repoGAL4/UASNotch^ICD^myc*. (F) (1) *w;UASmCD8GFP/+;alrmGAL4/+*, and (2) *w;UASmCD8GFP/+;alrmGAL4/UASNotch^ICD^myc*. (G) (1) Control = *tubGAL80^ts^; repoGAL4*; (2) larval glia>pros = *tubGAL80^ts^;repoGAL4/UASpros*.

To investigate the function of Pros in glial proliferation, we generated *pros^J013^* null mutant MARCM clones in larval glia. The number of IG in *pros^J013^* mutant clones (1–8 cells per clone in 8 clones generated in *n* = 786 VNCs) did not differ from the number of IG in wild-type clones (1–9 cells per clone in 15 clones in *n* = 1,254 VNCs). Furthermore, in wandering larvae (120 h AEL) the number of glial cells in *pros^voila1^/pros^S044116^* mutants was indistinguishable from wild-type (Figure 6A,D). These data demonstrate that Pros does not affect the extent of glial proliferation in the normal, non-stabbed larva. However, loss of *pros* function affected the timing of glial cell division (Figure S10A,B). In younger (96 h AEL) *pros^voila1^/pros^S044116^* mutant larvae, there were more glial cells than in wild-type (Figure S10A), implying that the excess glial cells arose from faster (but not more) cell divisions. Cell division is speeded up by shortening the G1 phase, for instance with the up-regulation of CycE. Consistently, Pros activates the expression of the CycE repressor Dacapo (the p21/p27 homologue) in glia (Figure S10C *p*<0.05). To further test if Pros can halt larval glial proliferation, we over-expressed *pros* in larval glia using *tubGAL80^ts^;repoGAL4*. This resulted in early larval lethality, and escapers had decreased glial number compared to controls (Figure 6G), showing that Pros inhibits glial proliferation. Altogether, our data show that Pros functions as a repressor of *cycE* in glia and it inhibits cell cycle progression by keeping glia arrested in G1 (Figures 6B).

If Pros inhibits cell division, why isn't there glial hyperplasia in the mutants? And why can't *pros* mutant glia proliferate upon injury? To solve this conundrum, we wondered if Pros might interact with Notch. Notch signalling is present in larval IG, its ligand Serrate is in axons (Figure S11A), and Notch maintains the expression of *pros*. Pros is also required for Notch signalling (Figure S11B–G), like in embryonic LG [10]. Thus, Notch and Pros maintain each other in IG. In other contexts, Notch promotes cell division by regulating the G1/S transition (Figure S10H) [43],[44]. We found that constitutive activation of Notch signalling—expressing the Notch Intra Cellular Domain (*Notch^ICD^*) —in all glia increased both total glial number (Figure 6A,Ai,D) and the number of Ebony+ IG (Figure 6E). Activation of Notch restricted to the IG only also resulted in an increase in IG cell number (Figure 6F). Consistently, transient activation of Notch signalling induced PNCA-GFP expression (Figure S10D,E) and BrdU incorporation (Figure S10F,G) specifically in IG. These data show that Notch can promote glial cell division.

So if Notch signalling is normally activated in IG, why don't they divide in the intact larva? Our data show that Pros and Notch have antagonistic functions in the control of glial proliferation. Since they also maintain each other, a “tug of war” between Notch and Pros is likely to keep IG in cell cycle arrest. To test this, we asked whether cell cycle arrest could be evaded by interfering with this feedback loop. Over-expression of *Notch^ICD^* in glia resulted in the up-regulation of Pros (Figure S11E), which would repress *cycE* expression. When we expressed *cycE* together with *Notch^ICD^*, this increased glial number and expanded VNC size (Figure 6A,D). Over-expressing *Notch^ICD^* in glia in *pros^S044116^* mutant larvae further increased glial number, causing a tumourous expansion of the VNC (Figure 6A,Ai,B). The increase in abdominal VNC size was not due to a non-autonomous effect on neuroblast proliferation or increased neuronal number (Text S2 and Figure S12 and Figure S13), but to increased glial divisions (Figure 6C). Altogether, our data show that Notch promotes cell cycle progression in glia while Pros inhibits it, and positive feedback between Notch and Pros counterbalances the effects of each other, maintaining glial cells on the brink of dividing (Figure 6B and Figure S10H). Interfering with this feedback loop has dramatic consequences in glial number and VNC size.

### The Gene Network: Pros Promotes Glial Differentiation

**Figure 7:**
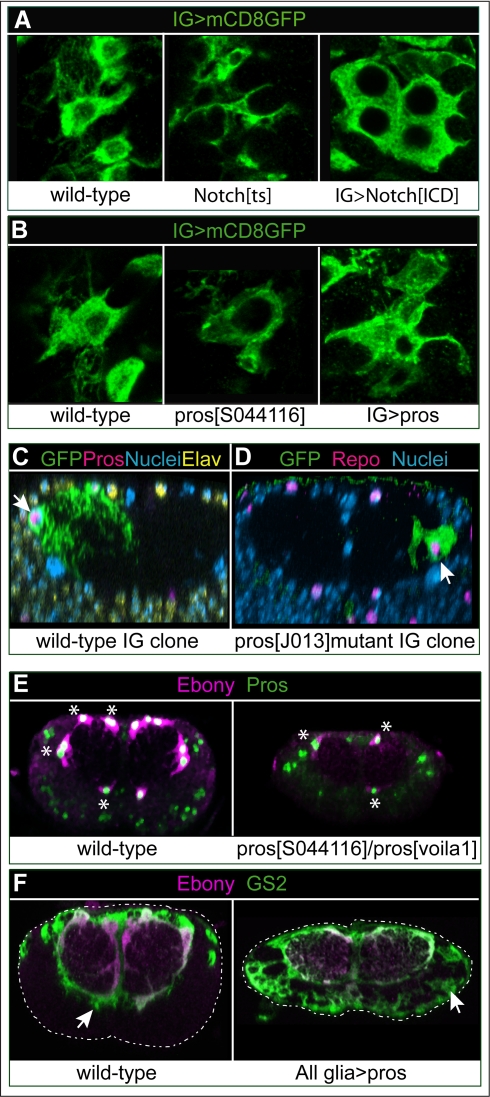
Pros promotes glial differentiation. (A,B) Notch and Pros have opposite effects in glial differentiation, seen here using membrane targeted mCD8GFP expressed by the IG driver *alrmGAL4* and detected with anti-GFP. (A) Glial differentiation is influenced by Notch, as filopodia and lamellipodia are thin in *Notch^ts1^* mutants, and cells are large and round up upon the over-expression of *Notch^ICD^*. (B) Pros is necessary for glial differentiation, as there is loss of IG filopodia in *pros^S044116^* mutants and more filopodia upon over-expression of *pros*. (C,D) Loss of *pros* does not affect glial number but prevents glial differentiation. Wild-type (C) and (D) *pros^J013^* null mutant MARCM clones showing that the number of progeny cells in a *pros^J013^* mutant clone (arrow) did not differ from the number of cells in a wild-type clone (quantification in text), while glial projections were deficient in the *pros* mutant clone, meaning that Pros is necessary for glial differentiation. Transverse views, nuclear dye is DAPI. (E) Ebony is virtually lost in *pros* hypomorphic mutants, and residual Ebony colocalises with residual Pros protein (asterisks). This shows that Ebony is a downstream differentiation target of Pros. (F) Over-expression of *pros* induces the expression of GS2, a differentiation marker for enwrapping glia (left image, arrow), in non-enwrapping glia outside the neuropile (right image, arrow indicates cortex glia). Dashed line indicates edges of the VNC, which was smaller in *pros* mutants. Genotypes from left to right: (A) (1) *w;alrmGAL4/UASmCD8GFP*, (2) *Notch^ts1^/Y;alrmGAL4/UASmCD8GFP*, and (3) *w;UASmCD8GFP/+;alrmGAL4/UASNotch^ICD^myc*. (B) (1) *w;*a*lrmGAL4/UASmCD8GFP*, (2) *w;*a*lrmGAL4/UASmCD8GFP; pros^S044116^/pros^S044116^*, and (3) *w;UASpros/UASmCD8GFP*; a*lrmGAL4/+*. (C) (1) *hsFLP tubP-GAL80 FRT19A/FRT19A*; *repoGAL4/UASmCD8GFP*, and (2) *hsFLP*;*actGAL4, UASGAPGFP/+;neoFRT82B tubGAL80/neoFRT82B pros^JO13^F263*. (F) (1) *w;tubGAL80^ts^/+;repoGAL4/+.*, and (2) *w;UASpros*/*tubGAL80^ts^; repoGAL4/+*.

To find out whether Notch and Pros influence IG differentiation, we visualised IG morphology using *alrm>mCD8GFP* upon loss or gain of function for each of these genes. To knockdown Notch function only in larvae, we used a temperature sensitive allele of Notch—*Notch^ts1^*. In *Notch^ts1^* mutant larvae, IG filopodia and lamellipodia are thinner than in wild-type controls (Figure 7A). Conversely, over-expression of *Notch^ICD^* in glia results in larger and rounder glial cells (Figure 7A). In hypomorphic *pros^S044116^* mutant larvae, IG hardly developed cytoplasmic projections (Figure 7B). Conversely, over-expression of *pros* in glia induced more elaborate IG projections (Figure 7B). These findings show that Notch and Pros have opposite effects on glial differentiation.

To further test how loss of *pros* function affects IG differentiation, we analysed MARCM clones of *pros^J013^* null mutant IG: glial morphology was aberrant, with dramatic loss of glial projections compared to wild-type (Figure 7C,D). The glial differentiation markers Ebony and GS2 were also influenced by Pros. Ebony is a glial enzyme involved in neurotransmitter recycling [45]–[48], and it was down-regulated in *pros^voila1^/pros^S044116^* mutants (Figure 7E and Figure S6B). GS2 is an enzyme involved in Glutamate recycling normally restricted to enwrapping glia [49]. Larval over-expression of *pros* with *tubGAL80^ts^; repoGAL4* induced its ectopic expression in non-enwrapping glia (Figure 7F). Over-expression of *Notch^ICD^* did not induce Pros, Ebony, or GS2 expression in non-enwrapping glia (Figure S14). Thus, GS2 and Ebony are directly regulated by Pros but not by Notch. Altogether, these findings demonstrate that Pros controls IG differentiation.

### The Gene Network: Pros-Dorsal/NFκB Feedback Enables Glia to Respond to Injury and to Restore G1 Arrest

**Figure 8:**
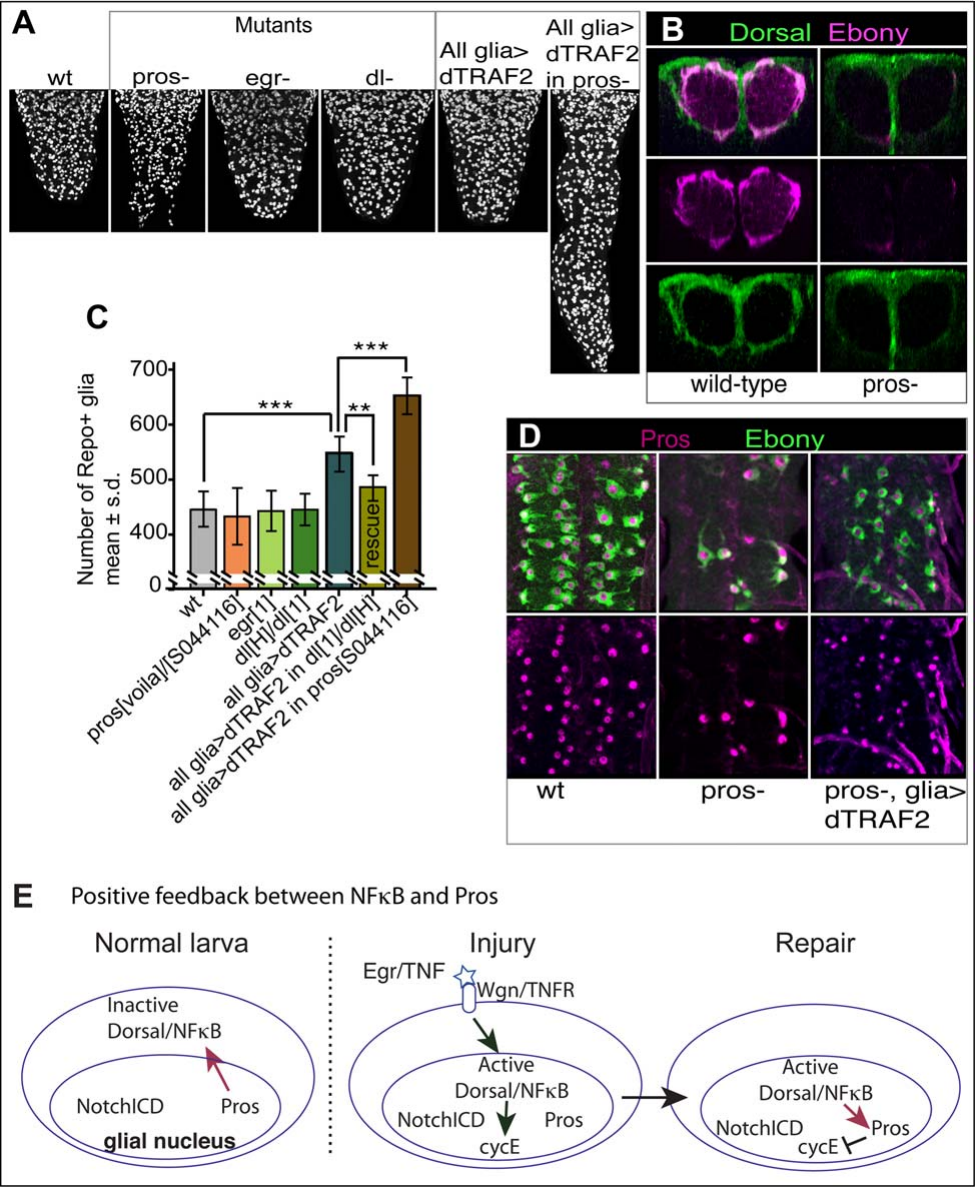
Pros-Dorsal/NFκB feedback enables glia to respond to Egr/TNF and restore arrest. (A,C) Activation of Dorsal by over-expressing *dTRAF2* in glia results in an increase in Repo+ glial number, which increases even further when *dTRAF2* is expressed in a *pros* mutant background. (A) Longitudinal projections of adominal VNCs stained with anti-Repo; (C) bar chart quantification of data in (A), plus the rescue experiment showing that the increase in glial number upon *dTRAF2* expression depends on Dorsal. (B) Dorsal is reduced from IG in *pros^voila1^/pros^S044116^* mutants, showing that its expression is postively regulated by Pros. Transverse view projections. (D) Hypomorphic *pros* mutants have residual Pros and Ebony in some glia, and over-expression of *dTRAF2* upregulates *pros* and *ebony* expression in many glial cells, demonstrating that Drosal activates *pros* expression. (E) Diagram showing the positive feedback loop between Pros and Dorsal. In the normal larva, Dorsal/NFκB is inactive in the cytoplasm, and its expression depends on Pros. Stabbing injury activates Dorsal/NFκB, which functions as a transcription factor to promote cell division. Dorsal/NFκB also activates *pros*, halting further cell division and restoring cell cycle arrest. All data are from wandering stage larvae. Error bars are standard deviation (s.d.); ** *p*<0.01; *** *p*<0.001. Sample sizes, statistical tests, and *p* values are given in Table S1. Genotypes from left to right: (A,C) (1) *yw*, (2) *pros^voila1^/pros^S044116^*, (3) *egr^1^*, (4) *dorsal^1^/dorsal^H^*, (5) *UASdTRAF2/+;;repoGAL4/+*, (6) *UASdTRAF2;dorsal^1^/dorsal^H^;repoGAL4/+*, and (7) *UASdTRAF2/+;; repoGAL4pros^S044116^/pros^S044116^*; (B) (1) *proslacZ/+*, (2) *pros^S044116^/pros^S044116^*. (D) (1) *yw*, (2) *pros^S044116^/pros^S044116^*, and (3) *UASdTRAF2/+;;pros^S044116^/pros^S044116^repoGAL4*. “All glia” stands for *repoGAL4* driven expression.

We have shown above that the glial proliferative response to injury is abolished in Egr/TNF, Wgn/TNFR, and Dorsal/NFκB mutants. The number of Repo+ glia, as well as the expression of GS2 and Ebony, were normal in *egr^1^* and *dl^H^/dl^1^* mutant wandering larvae (Figure 8A,C and Figure S9), meaning that the glial functions of Egr/TNF and Dorsal/NFκB are dormant in the normal, non-stabbed larva.

To investigate if activation of Dorsal/NFkB could promote glial proliferation, we over-expressed *dTRAF2* in all glia. The Drosophila TRAF6 homologue dTRAF2 binds Wgn/TNFR and induces the nuclear translocation of NFκB homologues [50]–[52]. When *dTRAF2* was expressed in glia, the number of glial cells increased (Figure 8A,C). This effect was rescued by expressing *dTRAF2* in a *dorsal^H^/dorsal^1^* mutant background (Figure 8C), showing that the effect of dTRAF2 is mediated by Dorsal. Activation of Dorsal/NFκB by expressing *dTRAF2* in glia resulted in an increased number of Ebony+ IG (*p*<0.01). Temporal over-expression of *dTRAF2* also induced BrdU incorporation in Ebony+ IG, showing that it activated mitosis cell-autonomously (Figure S15). These data show that activation of Dorsal by dTRAF2 promotes glial proliferation. Glial number and VNC size increased further upon glial expression of *dTRAF2* in *pros* mutants (*UASdTraf2;repoGAL4 pros^S044116^/pros^S044116^* Figure 8A,C), indicating that Pros antagonizes the proliferative function of Dorsal/NFkB (Figure 8E).

Our data show that Pros-Notch feedback keeps glia on the brink of dividing, and upon injury Egr/TNF signalling via dTRAF2 activates Dorsal/NFκB, tipping the balance towards cell division (Figure 8E). Thus we asked whether these two genetic mechanisms are linked. We found that in *pros^voila1^/pros^S044116^* mutant larvae Dorsal is decreased from IG (Figure 8B), suggesting that Pros is required for *dorsal/NF*κ*B* expression. Since the glial response to injury critically depends on Dorsal/NFkB, this means that the ability of IG to respond to injury is regulated by Pros (Figure 8E).

The glial regenerative response is constrained, as it induces glial proliferation but not tumours, indicating that cell cycle arrest is restored in daughter cells. Tumorous-like over-growth was induced by expressing *dTRAF2* in glia in *pros* mutants (Figure 8A,C), as was the case when expressing *Notch^ICD^* in *pros* mutants (Figure 6A,Ai,D). This suggested that Dorsal/NFκB might activate *pros* expression restoring arrest. To test this, we used hypomorphic *pros^S044116^* mutant larvae that still produce Pros at low levels in a few glial cells. Expression of *dTRAF2* in glia in *pros^S044116^* mutant larvae resulted in the up-regulation of Ebony and Pros (Figure 8D). These data show that Dorsal/NFκB activates *pros* expression in glia. Since Pros inhibits cell cycle progression whereas Dorsal/NFκB promotes it, the “tug of war” between Pros and Dorsal/NFkB is likely to restore G1 arrest in the daughter cells (Figure 8E).

Thus, we have shown that a gene network involving Notch, Pros, TNF, and NFκB controls the balance between glial proliferation, arrest, and differentiation.

### Repair: The Glial Response Is Regenerative and This Depends on Notch and Pros

**Figure 9:**
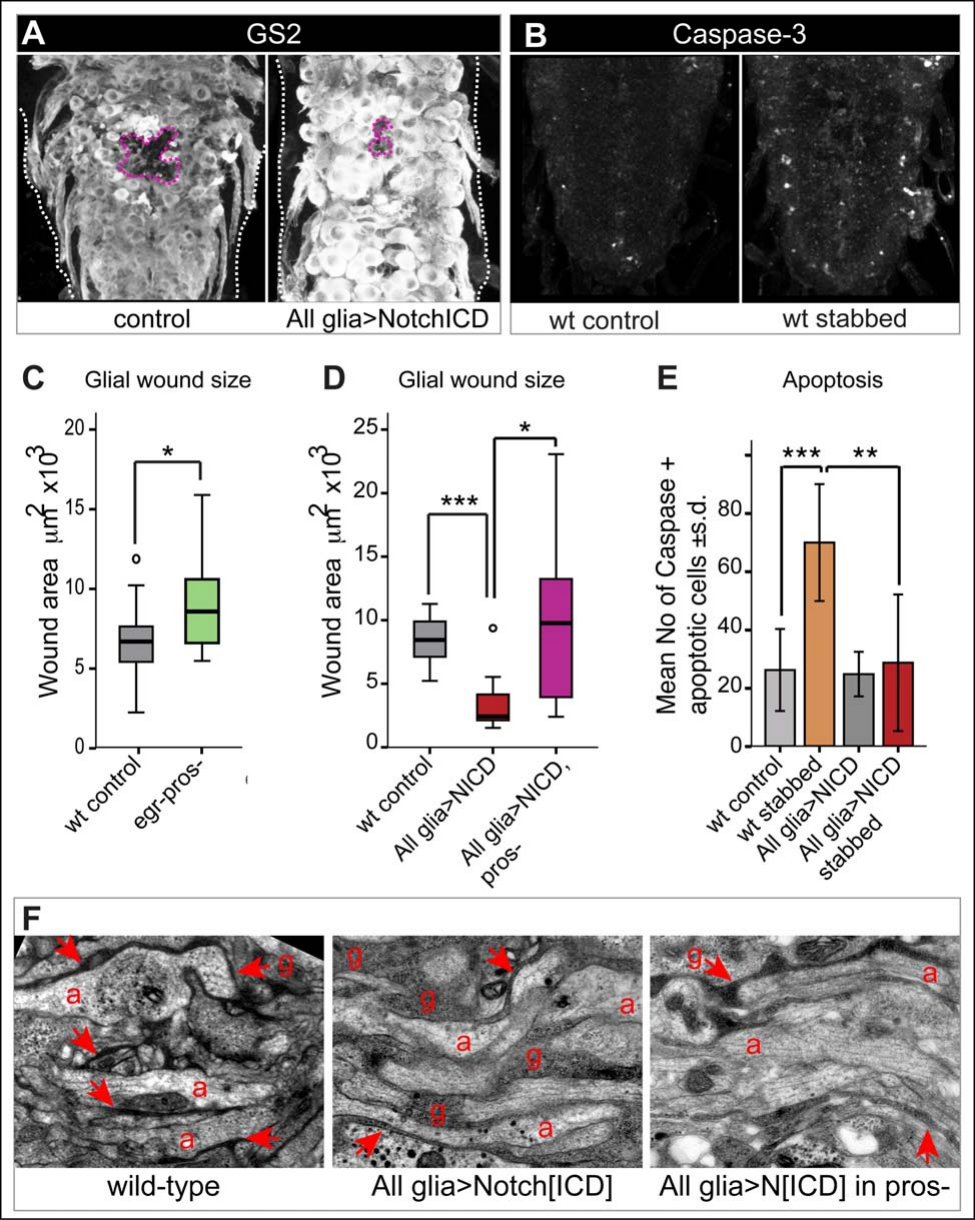
The glial response is regenerative and this depends on Notch^ICD^ and Pros. (A,C,D) The GRR gene network influences wound size. (A) The glial wound (pink dashed line) is visualised with anti-GS2, and it decreases upon expression of *Notch^ICD^* in glia (D, quantification). The white dashed line indicates the edges of the VNC. (C) The glial wound area is measured in VNCs labelled with anti-Ebony. Wound area increases in *egr^1^;pros^voila1^/pros^S044116^* double mutant larvae. (D) Wound area is measured with anti-GS2. It decreases upon over-expression of *Notch^ICD^* in glia, but it increases in larvae that are also mutant for *pros* (box plots around the median). (B,E) Expression of *Notch^ICD^* in glia does not rescue naturally occurring cell death, but it rescues stabbing injury induced apoptosis, visualised with anti-cleaved-Caspase-3; in (E) the number of apoptotic cells is quantified with DeadEasy Caspase. (F) Longitudinal TEM sections showing axons enwrapped by glia in wild-type (arrow); upon over-expression of NotchICD in all glia, glial enwrapment is considerably thicker and more prominent than in wild-type (arrows show a couple of examples, rest indicated by g = glia); however, enwrapment is deficient if *Notch^ICD^* is over-expressed in *pros* mutant larvae (arrows indicate persistent enwrapment), revealing that enwrapment depends on Pros. a, axons, off-white; g, glial cytoplasms and membranes, dark grey to black. (A–E) Wild-type larvae were stabbed at 96 h AEL and fixed 6 h later; controls in (B,E) are non-stabbed VNCs; (F) are wandering stage. (C,D) are boxplots; in (E) error bars are standard deviation (s.d.); * *p*<0.05, ** *p*<0.01; *** *p*<0.001. All glia> stands for expression driven by *repoGAL4*; wild-type is *yw*. Sample sizes, statistical tests, and *p* values are given in Table S1. Genotypes from left to right: (A) (1) *yw*, (2) *w;repoGAL4/UASNotch^ICD^myc*; (C) (1) yw, (2) *egr^1^;pros^voila1^/pros^S044116^*; (D) (1) yw, (2) *w;repoGAL4/UASNotch^ICD^myc*, (3) *pros^S044116^repoGAL4/pros^S044116^UASNotch^ICD^myc*; (E) (1) *yw*, (2) *w;repoGAL4/UASNotch^ICD^myc*. (F) prosS044116 repoGAL4/prosS044116UASNotclCDmyc.

To test whether manipulating this gene network was regenerative to enwrapping glia, we examined the glial wound. Stabbing disrupted the GS2+ Ebony+ glial mesh in the neuropile, and the area devoid of these markers was measured (Figure 9A). In *egr^1^; pros^voila1^/pros^S04416^* double mutant larvae, in which the proliferative glial response and glial differentiation were both affected, the glial wound increased significantly compared to controls (Figure 9C). In larvae expressing *Notch^ICD^* in glia, resulting in over-proliferation, the glial wound was consistently significantly smaller than in controls (Figure 9A,D). This indicates that either Notch itself or increased glial number is regenerative. We showed above that over-expression of *Notch^ICD^* in glia also induced *pros* expression, and that Pros promoted glial differentiation. Thus we asked whether the regenerative function of Notch relied on Pros. When stabbing was carried out in larvae that over-expressed *Notch^ICD^* in glia but were also mutant for *pros* (*repoGAL4 pros^S044116^/UASNotch^ICD^pros^S044116^*), wound size increased significantly (Figure 9D). Since glial cells proliferated in excess in this genotype (Figure 6A,D), this means that glial proliferation alone is not sufficient for repair and glial differentiation is also required.

To test what consequence the uncovered gene network might have on neuropile repair, we examined cell death levels. Apoptotic cells were visualized with anti-cleaved-Caspase3 antibodies, and counted in vivo automatically using purposely adapted DeadEasy Caspase software [53]. Injury increased the extent of apoptosis over non-injured controls (Figure 9B,E). Expression of *Notch^ICD^* in glia did not rescue baseline apoptosis, but it rescued injury-induced apoptosis (Figure 9B,E). This suggests that either Notch^ICD^ itself or the resulting increase in glial cell number is protective upon injury.

To test the effect of these genes in enwrapment, we used TEM. Over-expression of *Notch^ICD^* in glia dramatically increased glial projections and axonal enwrapment (Figure 9F). However, enwrapment was reduced when *Notch^ICD^* was over-expressed in *pros* mutant larvae (Figure 9F), indicating that Pros is required for enwrapment. Altogether, these data show that the glial response is regenerative, that both glial proliferation and differentiation are necessary for glial regeneration, and that Notch and Pros play central roles.

### Repair: Glial Proliferation and Differentiation Via the Gene Network Promote Neuropile Repair

**Figure 10:**
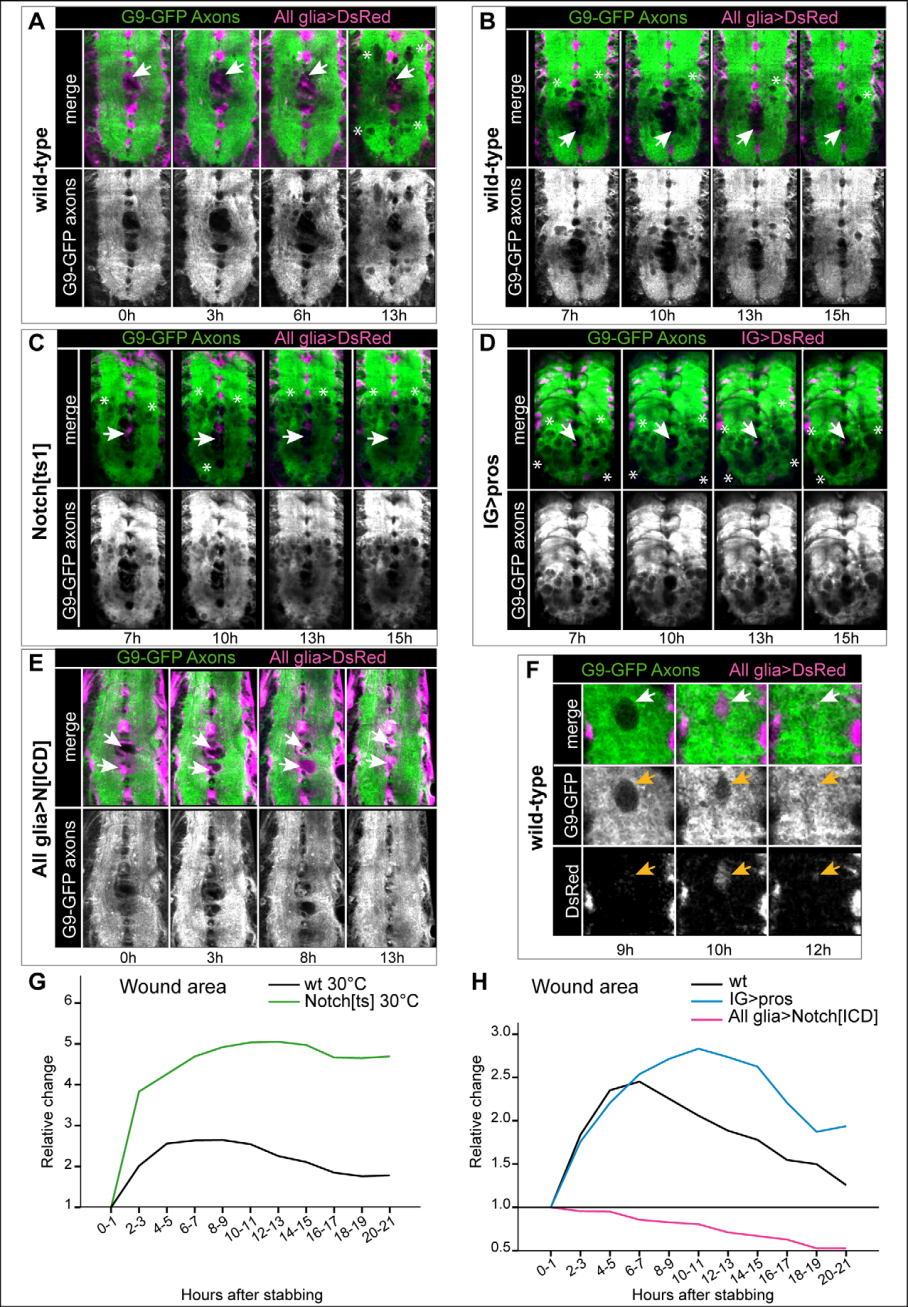
Glial proliferation and differentiation through the GRR gene network promote neuropile repair. (A,B and Video S1, Video S2) In wild-type, the stabbing wound (arrows) first enlarges; mutliple holes in the axonal neuropile appear in the process, many of which are filled in with glia, and may later disappear. Over time, the wound shrinks (arrows). (C and Video S5) In *Notch^ts1^* mutant VNCs, wound (arrows) size and vacuolisation increase compared to wild-type and do not shrink over time, exacerbating the damage. (D and Video S6) Upon over-expression of *pros* in IG (with *alrmGAL4*), which inhibits IG proliferation, the lesion (arrows) does not diminish over time either, and vacuolisation also expands. (E and Video S7) Over-expression of *Notch^ICD^* in glia prevents wound (arrows) enlargement and axonal vacuolization, reduces wound size, and promotes repair, here showing full axonal neuropile repair by 13 h. (F) Axonal holes that were filled with glia, heal (arrows). Higher magnification view of (B). (G,H) Temporal profiles of axonal neuropile repair after stabbing injury; controls are wild-type cultured VNCs. Wound area corresponds to the stabbing primary lesion plus vacuolisation. The data are normalised relative to the initial lesion per experiment. (A–F) are time-lapse snapshots of Videos S1, S2, and S5, S6, S7. Axons are visualised with the protein-trap line G9 driving GFP expression in all axons, and glia with DsRed driven by (A–C,E) *repoGAL4* or (D) *alrmGAL4*. Arrows indicate stabbing site. Asterisks indicate boundary of the area covered by original lesion plus vacuoles caused by stabbing. Total sample size per genotype, *n* = VNCs: *wt* 30°C *n* = 3–12; *Notch^ts1^ n* = 2–9; wt 25°C *n* = 3–6; *IG>pros n* = 7–21; *All glia>NotchICD n* = 6–18. Images are projections of several sequential confocal slices, with the time ellapsed after stabbing indicated under each panel. Genotypes: (A,B,F) *UASDsRed/+;G9/+;repoGAL4/+;* (C) *Notch^ts1^UASDsRed/Y;G9/+;repoGAL4*; (D) *UASDsRed/+;G9/UASpros;alrmGAL4/+*; (E) *UASDsRed/+;G9/+;repoGAL4/UASNotch^ICD^myc*.

To test what effects the glial regenerative response (GRR) may have on the axonal bundles, we carried out time-lapse recordings of stabbed larval VNCs, with glial cells labeled with *repoGAL4>* or *alrmGAL4>UAS-DsRed*, and all axons labeled with the GFP-protein-trap line G9. In wild-type larvae the neuropile wound first increased in size, and numerous vacuoles formed, consistently with TEM and confocal microscopy data from fixed samples (Figure 10A,G,H and Video S1, refer also to Figure 2E and Figure 3D,F,G). Subsequently, the vacuoles might disappear and the wound might shrink (Figure 10B,F,G,H and Video S2). In *Notch^ts1^* mutant larvae, wound size in the axonal neuropile was considerably larger, had greater vacuolization than controls, and did not decrease over time (Figure 10C,G and Video S5). Similarly, when glial proliferation was prevented by over-expressing *pros* in IG, wound size and vacuolization were also more extensive than in controls (Figure 10D,H and Video S6). Conversely, when glial cell proliferation was increased by over-expressing *Notch^ICD^*, wound enlargement and vacuolization were considerably constrained, wound size decreased over time, and even repaired (Figure 10E,H and Video S7). In both wild-type and upon over-expression of *Notch^ICD^* there was a correlation between repair and presence of DsRed+ glial processes within or around the vacuoles and in areas of axonal damage (Figure 10F and Video S2, Video S7). Together with the TEM data (Figure 3D–N), the time-lapse data suggest that upon injury, glial processes engulf the vacuoles, and phagocytose axonal fragments and other cellular debris, contributing to repair. Altogether, our data show that Notch and Pros control glial proliferation and differentiation required for glial regeneration and debris clearance, and this enables neuropile repair.

## Discussion

We present here a regenerative response of Interface Glial cells to stabbing injury in the Drosophila CNS, we have identified an underlying gene network, and we show that it can promote repair. This gene network controls the balance between glial proliferation, arrest, and differentiation, and it promotes repair by “hitch-hiking” on a developmental mechanism for structural robustness.

### The Uncovered Gene Network Promotes Glial Regeneration and Neuropile Repair

**Figure 11:**
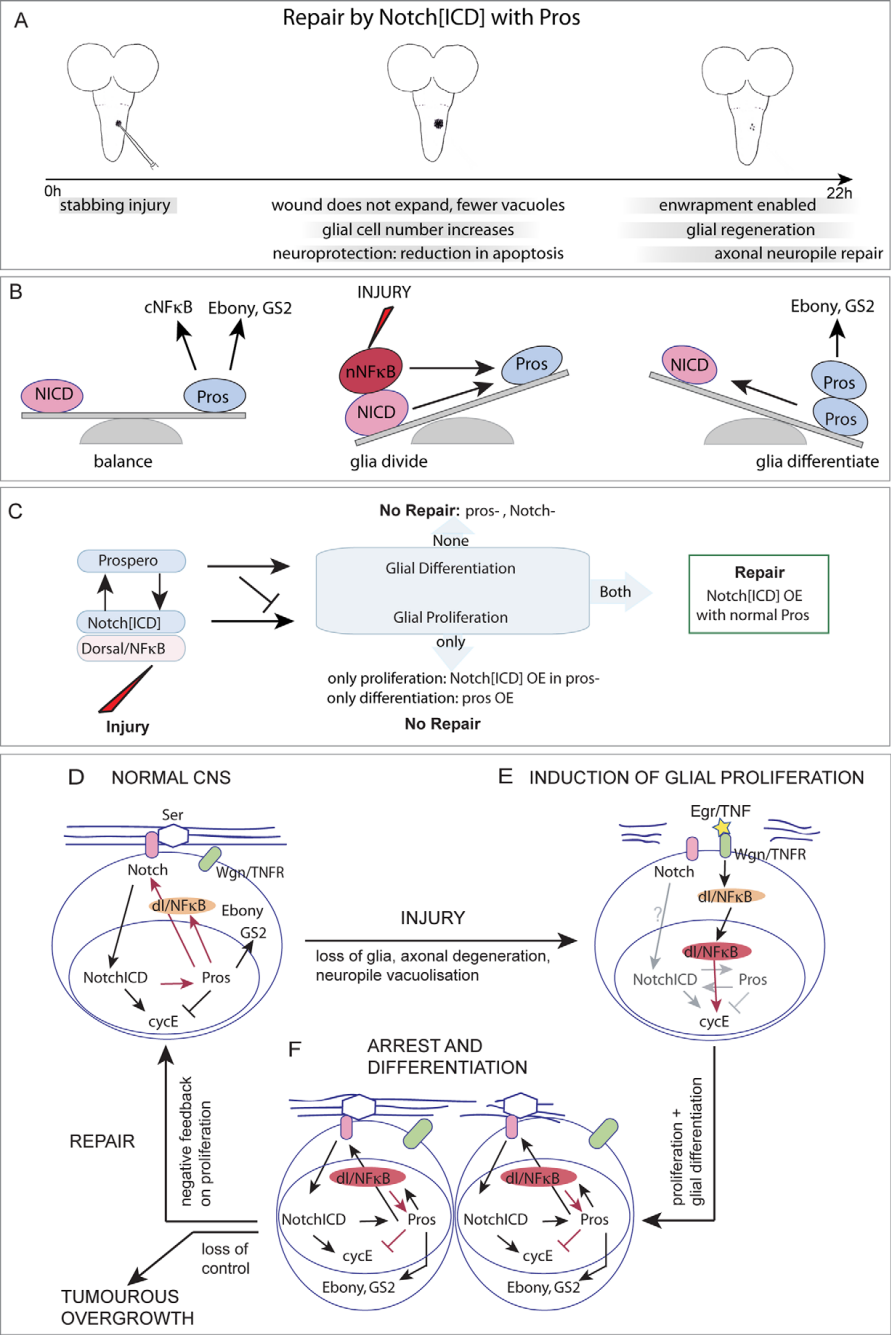
The GRR gene network is a homeostatic mechanism for structural robusntess that enables the glial regenerative response to CNS injury. (A) Over-expression of Notch^ICD^ in glia in the presence of Pros promotes repair. (B) In the normal intact larva, the balance between Notch^ICD^ and Pros keeps IG in the brink of dividing. Pros also promotes the expression of cytoplasmic NFκB and of the glial differentiation factors Ebony and GS2. Upon injury NFκB translocates to the nucleus increasing the relative levels of cell cycle activators, and glia divide. NFκB and Notch^ICD^ activate Pros expression, and as Pros levels rise, Pros halts further glial cell division and promotes glial differentiation. Pros also promotes *Notch* expression, thus restoring the original balance. (C) Altering normal gene function prevents or enhances repair. When glial proliferation and differentiation are both disrupted in *Notch^ts1^* or *pros* mutants, both the glial and axonal lesions enlarge, exacerbating the damage. Promoting only glial proliferation (with Notch^ICD^) or differentiation (with Pros) is not sufficient for repair. The regenerative effect of Notch^ICD^ depends on increasing glial number plus Pros inducing glial differentiation. (D,E,F) Feedback loops result in a homeostatic mechanism for structural robustness: (D) Pros activates the expression of *Notch* and Notch^ICD^ activates the expression of *pros*, and as they have opposite functions in the cell cycle this positive feedback maintains glia with a potential to divide. Pros and Notch^ICD^ levels do not rise uncontrollably as axon-glia interactions between Serrate and membrane Notch constrain the levels of nuclear Notch^ICD^ (negative feedback). Pros also activates the expression of NFκB in glia, which is inactive in the cytoplasm but primes glia to respond to injury. (E) Injury induces the activation of Wgn/TNFR by Egr/TNF, and the nuclear translocation of NFκB, which promotes glial proliferation. (F) Dorsal/NFκB also activates the expression of *pros*, and as the levels of Pros rise in daughter cells, this halts further cell division. This in turn re-establishes the Pros-Notch loop and cell cycle arrest. Pros promotes glial differentiation inducing axonal enwrapment and enabling future glial responses to injury (e.g. phagocytosis). Although Pros and NFκB maintain each other (positive feedback), their levels are constrained as NFκB is normally kept inactive in the cytoplasm (negative feedback). The cell cycle activators Notch and Dorsal/NFκB activate the expression of the cell cycle inhibitor Pros, providing negative feedback on cell division. When these feedback loops break down, tumourous over-growth ensues. Thus this gene network is homeostatic.

Stabbing injury in normal larval VNCs caused an initial loss of IG, wound expansion, and neuropile vacuolization. Ensheathing glia extended large processes within the neuropile, phagocytosed axonal fragments and cellular debris and dissolved vacuoles, some remaining glial cells divided, and neuropile integrity could be restored. This natural mechanism was enhanced by activating Notch signalling in glia in the presence of Pros. Together, Notch^ICD^ and Pros prevented wound enlargement and vacuolization, they prevented injury induced apoptosis, increased ensheathing glial number, and promoted glial regeneration and axonal neuropile repair (Figure 11A). Remarkably, the stabbing injury wound could be completely repaired in these larvae. This was achieved through the balance of glial proliferation and differentiation under the control of Notch and Pros (Figure 11B). Notch^ICD^ promotes glial proliferation and Pros promotes their differentiation. In the normal intact larva, the balance between Notch^ICD^ and Pros keeps IG in the brink of dividing. Pros also promotes the expression of cytoplasmic NFκB and of the glial differentiation factors Ebony and GS2. Upon injury NFκB shuttles to the nucleus increasing the relative levels of cell cycle activators, and glia divide. NFκB and Notch^ICD^ activate Pros expression, and as Pros levels rise, Pros halts further glial cell division and promotes glial differentiation. Pros also promotes *Notch* expression, thus restoring the original balance. We have shown that interfering with the functions of these genes prevents repair (Figure 11C). When both glial proliferation and differentiation were inhibited as in *egr-pros-* double mutant larvae, the glial wound enlarged. When glial proliferation was abolished in *Notch^ts^* mutants or upon over-expression of *pros* in glia, the glial and neuropile wounds enlarged and vacuolisation increased. Conversely, increasing glial proliferation by activating Notch signaling promoted glial regeneration. The regenerative effect of Notch not only relied on the increase in glial cell number, but also on *pros*. Glial regeneration was prevented if *Notch^ICD^* was expressed in a *pros* mutant background. Pros promotes IG differentiation, increasing the complexity of cytoplasmic processes and promoting axonal enwrapment. In the absence of Pros glia have fewer filopodia and lamellipodia, and downregulate the glial differentiation marker Ebony—an enzyme involved in the recycling of neurotransmitters [45]–[48]. Conversely, upon over-expression of *pros*, glia have more processes and up-regulate the expression of the enwrapping glial marker GS2—and enzyme involved in the recycling of Glutamate [49]. The control of glial differentiation by Pros is conceivably required for glia to phagocytose and clear cellular debris, restore neurotransmitter homeostasis, and re-enwrap the neuropile and axons. Thus, the uncovered gene network is regenerative and Pros is the critical link in the control of glial proliferation and differentiation that enables repair.

### How the Glial Regenerative Response Gene Network Operates

The identified gene network enables the regenerative response to injury in the following way. (1) In the normal larva it maintains glial cells arrested with mitotic potential, enabling them to respond to injury (Figure 11D). Generally, a cell that has exited the cell cycle cannot divide again. Pros and Notch together prevent cell cycle exit and maintain glia in a proliferative yet arrested state. That is, the IG can divide, but do not normally do so. This state is achieved as Pros and Notch maintain each other but have antagonistic functions on the cell cycle. Pros prevents cell cycle progression by repressing *cycE*, and Notch promotes the G1/S transition. Their mutual maintenance counterbalances their effects on the cell cycle, and maintains glial cells on the brink of dividing. (2) The gene network enables IG to respond to injury by proliferating at the lesion site (Figure 11E). This is achieved as Pros regulates the expression of *dorsal*/NFκB. Dorsal/NFκB is a transcription factor located in the cytoplasm in its inactive form. Upon injury, the pro-inflammatory cytokine Egr/TNF via its receptor Wgn/TNFR induces the translocation of Dorsal/NFκB to the nucleus, where it promotes cell cycle progression. This breaks the balance of the Pros-Notch loop pushing glia to divide. (3) The gene network restores cell cycle arrest, preventing uncontrolled proliferation (Figure 11F). Dorsal/NFκB activates the expression of *pros*, which inhibits cell cycle progression. Presumably as the total input from cell cycle activators (Notch and NFκB) and inhibitor (Pros) balances out, it restores cell cycle arrest. The antagonistic function of Pros versus Notch and Dorsal/NFκB, and their mutual dependence, restricts cell proliferation. Overgrowth is induced when negative feedback breaks down, upon activation of Notch or Dorsal/NFκB in the absence of Pros. Thus the GRR gene network prevents tumourous overgrowth. (4) The gene network controls glial differentiation, which critically depends on Pros. We have shown that in the absence of Pros, IG have reduced processes, and wound size and vacuolization enlarge. This would indicate that Pros may be required for the phagocytic response of glia and lesion clearance. We have also shown that Pros is required for axonal enwrapment, an end point of repair. Thus the gene network is a homeostatic cycle by which injury triggers a response in glia that not only repairs the wound but also primes the restored glia to respond to further injury or smaller changes. This would suggest that there must be a mechanistic link between Pros and the corpse engulfment pathway of Simu, Draper, Src42A, and Shark [4],[6]–[9]. While our data show that the IG underlie the regenerative response, we cannot rule out that other glial classes might also be involved. For instance, cortex glia have also been reported to be phagocytic and activate Draper [8]. It will be interesting to explore these uncovered research avenues in the future.

### Lineage Specific Relationships of Pros Determine Cellular Outcome

Drosophila Pros and the mammalian homologue Prox1 have different effects on cell proliferation in different cell lineages [10],[37]–[40],[54]–[56]. In Drosophila ganglion mother cells, Pros promotes cell cycle exit by repressing *cycE* expression, and is a tumour suppressor [37]–[40]. However, in Drosophila embryonic glia Pros enables cell division [10], but how this might occur remained unexplained. Our findings demonstrate that Pros functions as a repressor in *cycE* also in glia. However, in IG loss of *pros* does not result in hyperplasia because as Pros maintains Notch signaling, loss of *pros* leads to loss of Notch signaling, consequently reducing cell cycle activation. Although glial cell division initially occurs faster in *pros* mutants as the G1 phase shortens upon the de-repression of *cycE*, cell division soon stops due to the loss of Notch. The diverse outcomes of Pros function depend on cell type specific gene networks.

### The GRR Gene Network Is a Homeostatic Mechanism for Structural Robustness

We have shown that in normal larvae the IG do not divide and that loss of function mutations in the gene network genes can result in normal glial cell number and distribution of glial markers. It would appear that these glial gene functions are uncovered upon injury. However, since in the wild CNS injury most likely results in fruitfly death, this raises the intriguing question of what might this gene network be for in the non-injured fruitfly. The fact that the glial regenerative response is also found in other insects, fish, and humans might imply an underlying common genetic mechanism, but why should it be?

Our findings suggest that the GRR is a homeostatic mechanism that promotes structural robustness in the non-injured animal. Homeostasis is grounded in two features of the feedback loops. Firstly, both feedback loops result in negative feedback on cell proliferation. Injury initially causes cell loss, and glial proliferation and differentiation are subsequently induced followed by cell cycle arrest, restoring cell number and enwrapment. This is achieved as the cell cycle activators (Notch and NFκB) induce the expression of a cell cycle inhibitor (Pros). Thus, although glial cells divide initially, as more activator protein is produced, more inhibitor is produced too, restoring normal cell number but halting further cell division (Figure 11C). This homeostatic control enables glial proliferation for repair while preventing excess, which would result in tumours.

Secondly, the two feedback loops limit the amounts of cell cycle regulators. The mutual maintenance between Pros and Notch, and Pros and NFκB would result in their levels forever rising. Instead, the two positive feedback loops are constrained, in different ways. Pros-Notch feedback is established spatially through interactions between enwrapping glia and the axons that express the Notch ligand Ser. Pros is a transcription factor and can directly activate the expression of Notch. However, Notch only functions as a transcription factor after it has been cleaved at the membrane as Notch^ICD^, which then translocates to the nucleus. Thus positive feedback only takes place when Notch contacts Ser in neighbouring axons. If contacts with Ser are saturated, Notch^ICD^ is not processed and cannot activate Pros. In this way the Pros-Notch loop is stabilized relative to the amount of Ser in axon-glia contacts.

Pros-NFκB feedback is constrained through time, in response to changes in the cellular environment. Although NFκB is a transcription factor, it can only activate Pros expression when it translocates to the nucleus. In the uninjured glia, NFκB is trapped in the cytoplasm and cannot activate *pros*. In this way, Pros-NFκB positive feedback is frozen in time and released only in an injury event.

Following injury, the homeostatic feedback loops restore initial conditions. Injury is likely to compromise contact between axons and glia, perhaps causing an initial drop in Notch signalling in glia, which would consequently down-regulate Pros levels. But injury also triggers the nuclear shuttling of NFκB, inducing cell proliferation and *pros* expression. As Pros levels rise, it halts further cell division and promotes glial differentiation and the expression of Notch. As glial differentiation restores neuron-glia interactions, this activates Notch signalling in glia, re-establishing the Pros-Notch loop, cell cycle arrest and priming glia for future responses to injury, thus closing the cycle.

In normal, non-injured larvae, the IG divide sporadically, and these divisions may represent homeostatic adjustments in glial number in response to cellular changes due to genetic variability or exogenous influences (e.g., changes in temperature). This would help clear debris and neurotransmitters, maintain axonal enwrapment and ionic homeostasis, modulate axonal growth and fasciculation, and provide trophic support to neurons. It would maintain enwrapment of the neuropile preserving architecture. This adjustment could result from fluctuations in axon-glia interactions that altered the relative levels of Notch^ICD^ and Pros. We propose that the normal function of the GRR gene network is the homeostatic regulation of glial proliferation and differentiation to provide structural robustness. The glial regenerative response to injury may “hitch-hike” on this developmental mechanism to restore structural integrity. This would explain why a gene network underlies these events and how it emerged in the course of evolution—as a mechanism that confers robustness, reproducibility, and reliability to CNS structure.

### The Glial Regenerative Gene Network May Be Conserved in Mammalian Glia

We have shown that IG can enwrap the neuropile, axonal bundles, and individual axons. Similar glia in insects have been compared to mammalian oligodendrocytes [57],[58]. However, IG do not form nodes of Ranvier, thus resembling non-myelinating enwrapping glia, such as Remak glia in the peripheral nervous system (PNS) and olfactory ensheathing glia of the CNS [15]. Drosophila IG also express molecules involved in neurotransmitter recycling such as Ebony and Glutamate synthetase (GS2). In vertebrates, neurotransmitter reuptake is mostly carried out by astrocytes in the CNS and Schwann cells in the PNS [59]. NG2+ OPCs and oligodendrocytes also express molecules involved in glutamate recycling, including glutamate synthetase, at non-synaptic sites [60]–[62]. Like microglia and astrocytes in mammals, and Drosophila ensheathing glia in other contexts [63]–[65], Drosophila IG are phagocytic and engulf axonal and other debris [66]. And like Schwann cells, Drosophila Pros+ IG can function as differentiated cells but can also divide. Thus, Drosophila IG cells are neuropile glia that behave like, and carry out multiple functions attributed to, distinct glial types in mammals.

The injury progression we observed—wound expansion, followed by debris clearance, glial regeneration, and neuropile repair—reproduces that documented for insect [12],[13] and mammalian CNS injury [67]. The molecular mechanisms underlying debris clearance have been little explored in mammals [68]. Although astrocytes proliferate to some extent upon injury [18],[19], NG2+ OPCs are the prominent cell type to divide [18],[19],[69]. Replenishing ensheathing glia promotes axonal regrowth and insulation, and protects against axonal degeneration and neuronal death. Thus, transplantation of enwrapping glial progenitors is a relevant strategy for the therapeutic treatment of spinal cord injury and demyelinating diseases [20],[22]. A critical aim has been to identify a gene interacting with Notch1 that will enable differentiation of progenitors into enwrapping oligodendrocytes. Our Drosophila findings have revealed shared functions of Notch between insect IG and NG2+ OPCs: as with IG, Notch1 maintains the mitotic state of OPCs [33]; it is present in OPCs that divide upon injury [34],[35]; and it prevents oligodendrocyte differentiation [70]. We show here that Pros antagonizes Notch function and it has a critical role inducing glial differentiation in fruitflies. The vertebrate homologue Prox1 can promote cell cycle exit and differentiation [54],[71] and antagonize Notch1 function in mammalian neural stem cells [72]. If a gene network similar to that uncovered here operates in human glial progenitors, its manipulation may facilitate CNS repair. The GRR gene network should also bring insights into the understanding of glioma, as Notch, NFκB, and *cycE* are hyper-activated in human gliomas [73],[74].

Finally, like in Drosophila, the response to spinal cord injury by mammalian OPCs recapitulates developmental events [69]. The physiological function of enwrapping glial plasticity in the adult may be to promote re-enwrapment following focal loss and to modulate myelination also during learning [15],[75]. Similarly, the clearance of axonal degeneration by glia during circuit remodeling shares mechanisms with that after injury [4],[66]. Accordingly, the GRR gene network may be a common, homeostatic mechanism for structural robustness and plasticity.

Supporting Information is linked to the online version of the article. Supporting Information comprises Figures S1 to S15, Table S1 with statiscal analyses, Videos S1 to S7 and TextS1 and Text S2 with data, and Text S3 with detailed methods.

## Materials and Methods

Details on methods can be found in Text S3. A summary is given below.

### Genetics

Conventional genetics was used to generate lines of flies bearing multiple mutations, drivers, and other genetic tools. For the description of genotypes and crosses, please see Text S3.

### Temporal Control of Gene Expression in Larval Stages Only

To drive gene expression in larval stages only, thus enabling normal embryogenesis, we used (1) the temperature sensitive GAL4 repressor GAL80ts driven by the general *tubulin* promoter in *tubGAL80ts* flies (Bloomington) [76]; GAL80ts represses GAL4 at 18°C but not at 30°C, so larval GAL4 expression is controlled by shifting larvae from 18°C to 30°C at the required time. (2) *hsGAL4* (gift of S. Brogna) flies, where GAL4 is switched on after heat-shock at 37°C. (3) Notch^ts1^. For details, see Text S3.

### MARCM Clones

Eggs were collected for 6 h and kept at 18°C until heat-shock was applied to the larvae. Mosaic Analysis with a Repressible Cell Marker (MARCM) clones were generated as described [77]; for genotypes, see Text S3.

### Stabbing Injury to the Larval Ventral Nerve Cord

VNCs were dissected from 96 h AEL old larvae (unless otherwise indicated elsewhere) in Shields and Sang M3 insect culture media (Sigma). The VNCs were stabbed from the dorsal side with a fine tungsten needle (Fine Scientific Tools), of 0.5 mm diameter at the base and 1 µm diameter at the tip. Culture of dissected injured or uninjured VNCs was done according to [78] with the indicated adaptations. Each brain was cultured separately in a well (24-well plate) containing 500 µl culture medium with 7.5% fetal bovine serum (Sigma), 1% Penicillin, and streptomycin (Sigma) for 18 to 22 h at 25°C. Control VNCs were dissected and cultured in the same way, without stabbing injury. Following culture, VNCs were fixed and stained as normal.

### Immunohistochemistry, In Situ Hybridization, and BrdU Incorporation

Immunohistochemistry, in situ hybridisations to mRNA and BrdU incorporation experiments of larval VNCs were done following standard procedures. Samples were mounted either in Vectashield with the nuclear dye DAPI (Vector Laboratories) or in 80% Glycerol PBS after staining nuclei with Daunomycin 5 µg/ml (Sigma) or Hoechst33342 5 µg/ml (Sigma). For BrdU detection, the VNCs were treated with 2 M HCl for 20 min at room temperature after immunolabeling for other proteins. Samples were mounted either in Vectashield with DAPI (Vector Laboratories) or in 80% Glycerol PBS after staining nuclei with Daunomycin 5 µg/ml (Sigma) or Hoechst33342 5 µg/ml (Sigma). For antibodies used, details on plasmids, and other, see Text S3.

### Microscopy

Bright field, laser scanning confocal and transmission electron microscopy, and image processing were done following standard procedures (more details in Text S3).

### Time-Lapse Confocal Microscopy Recordings

Time-lapse confocal laser scanning throughout the entire neuropile was done visualizing axons with G9 (gift of W. Chia) [79], an protein-trap line with GFP in all CNS axons, and all glia (except midline glia) with repoGAL4/UASDsRed. Experimental genotypes were generated by crossing G9; repoGAL4/TM6B flies to the following flies: (1) *UASDsRed S197Y* (gift of K. Ito) [80], (2) *UASDsRed S197Y*;*UASNotch^ICD^myc*, (3) *Notch^ts1^ UASDsRed S197Y/FM7(sn+)actGFP*, and (4) by crossing *G9*;*alrmGAL4* (*alrmGAL4* is a gift of Marc Freeman) [64] to *UASDsRed S197Y; UASpros*. The stabbed VNCs were placed dorsal side down in a 35 mm glass based dish (Iwaki) treated with PolyLysine (Sigma). Time-lapse scans (xyzt scan) were carried out using a Leica SP2-AOBS confocal inverted microscope with a temperature controlled chamber set to 25°C, or to 30°C for *Notch^ts1^* and its control experiments, 1- to 2-h intervals per Z-scan (8 time points in average), 20× lens with 4 times zoom, and 1 µm interval between optical slices. The obtained images were processed in ImageJ using plugins Turboreg and Stackreg to correct accidental sample movement. The Videos were arranged using ImageJ and Adobe Photoshop.

### DeadEasy for Automatic Counting of Glial and Apoptotic Cells and Other Quantifications

To count automatically the number of all larval glia stained with anti-Repo and acquired as confocal microscopy images, we purposely wrote DeadEasy Larval Glia software in Java as an ImageJ plug-in. Confocal serial sections were obtained with the BioRad Radiance 2000 confocal microscope as images of x,y = 0.5665 µm/pixel and z = 1 µm/pixel dimensions. The region of interest (ROI) was defined as the region starting from immediately posterior to the Ebony-positive ventral IG of the last thoracic segment to the posterior tip of the VNC, hereby referred to as abdomen. Peripheral nerves exiting the VNC were excluded from the ROI. DeadEasy Larval Glia identifies the stained cells first in 2-D based on shape (circular or elliptical) through each confocal slice and then in 3-D throughout the stack, based on minimum and maximum volume and minimum pixel intensity. DeadEasy creates a stack of processed images, where the identified objects reproduce those of the Repo glia in the raw images, enabling easy comparisons and validation. Each cell can be uniquely identified, as placing the mouse over each cell highlights a number, with which it is possible to check if, for example, two adjacent cells are counted as one. The programme was validated using n = 997 cells out of 3 different stacks of images. The mathematical algorithm for DeadEasy Larval Glia will be published elsewhere (Forero, Kato, and Hidalgo, in preparation).

To count the number of dying cells (labeled with active Caspase3-positive) throughout the VNC, we adapted the programme DeadEasy Caspase [81] to work on larvae. Given the strong variation in background intensity in larval samples, the outlier thresholding method originally used for embryos did not provide good results under the new conditions. Entropy thresholding was used instead, which provided better results. However, some other labeled tissues could not be rejected automatically anymore, given that they did not have a particular shape or size which would have allowed one to differentiate them from the apoptotic cells. Poor signal-noise ratio due to thickness of larval VNCs also resulted in false negatives/positives in deeper slices. Therefore, we manually corrected the error by deleting false positive and adding back false negatives for the final counting, employing the ImageJ DeadEasy Manual macro (described below).

Anti-Ebony stained IG were counted manually with support of the ImageJ DeadEasy Manual macro, which we purposely developed to speed up manual counting and eliminate error. With this macro, Ebony positive cells in a confocal stack of images are labelled manually with a digital colour, and DeadEasy Manual software automatically counts the colour labels.

Wound area was measured on longitudinal confocal microscopy images of the neuropile, using the ROI manager in ImageJ, for the glial wound on anti-Ebony and anti-GS2 stained VNCs, and for the axonal wound and vacuoles in time-lapse on G9-GFP-expressing VNC. The largest outline of the wound throughout the stack of images (i.e., a neuropile) was set as the ROI and measured in µm^2^. The kinetics of the area affected by the wound and vacuoles were obtained by normalising the size of affected area at each timepoint to the size of the area at 0–1 h after stabbing.

### Statistical Analyses

For statistical tests applied to each experiment and *p* values, please see Text S3 and Table S1.

## Supporting Information

Figure S1 3-D rendering of the stabbed Ventral Nerve Cord. Snapshots from Videos S3 and S4 showing that stabbing creates an injury at the dorsal entry site and a secondary injury ventrally (arrows), and results in GS2+ IG loss (asterisks).
(TIF)

Figure S2 The IG can be visualised with Pros, Ebony, Repo, and *alrmGAL4*. (A) *AlrmGAL4>dsRed* drives expression in Pros+ Interface Glia as seen by colocalisation of dsRed, Pros, and Ebony in these cells. (B) Confocal longitudinal sections through different focal planes in the VNC, from dorsal to ventral, to show the IG sub-types. IG co-express the nuclear transcription factor Pros and cytoplasmic Ebony involved in neurotransmitter recycling (arrows). (C) Tranverse view showing colocalisation of Ebony and Repo in IG (arrows) around the axonal neuropile (N); the IG nuclei are located outside, glial processes project into the neuropile. dIG, dorsal Interface Glia; lIG, lateral Iterface Glia; vIG, ventral Interface Glia. Genotype for (A): *UASDsRed/+; alrmGAL4/+*.
(TIF)

Figure S3 IG do not normally divide but remain in G1 arrest. (A,B) The mitotic marker anti-phospho-Histone-H3 (pH3) and MARCM clonal analysis show that the incidence of IG division is rather low in wild-type non-stabbed larval VNCs, meaning that generally IG do not divide. (A) Proliferation of IG could be seen in wild-type larvae by colocalisation of the glial marker anti-Repo and pH3 (arrowhead) at the mid-third instar stage, but only 0.6% VNCs had one mitotic IG (*n* = 181 VNCs), whereas 44% VNCs had at least one pH3 positive glial cell including other glial types. Confocal transverse views through the dorsal half of the VNC neuropile and the neuropile edge is revealed with the nuclear die DAPI (blue). (B, Bi higher magnification) GFP-labelled MARCM clones were induced by heat-shock at the early third instar stage and specimens were fixed at the wandering stage. The GAL4-dependent expression of GFP is repressed by the inhibitor GAL80 and repression is relieved when mitotic recombination is induced by *hs-FLIPase*. Thus, presence of GFP in IG implies that mitosis occurred. Here a two-cell clone is shown, and cytoplasmic projections enwrap the neuropile and project within. IG clones are identified by the exclusion of the neuronal marker Elav (arrowheads) and their enwrapment of the neuropile; the nuclear dye is DAPI. 15 wild-type VNCs had one IG clone; *n* = 1,254 VNCs were analysed. (C) To test whether glial cells were arrested with mitotic potential, we asked whether transient expression of *cycE* in larval glia resulted in an increase in IG entering S-phase as measured with a brief BrdU pulse. Using temperature sensitive GAL80^ts^, larvae were shifted at the restricted temperature and treated with BrdU for 6 h. Transient expression of *cycE* in glia at the mid-third instar larval stage resulted in a significant increase in Ebony+ IG that incorporated BrdU compared to wild-type controls. These data show that in the normal larval CNS, IG are mostly arrested, have not terminally exited the cell cycle, and can be induced to divide. (D) Quantification of data in (C). Genotypes: (A) wild-type, *yw*; (B) MARCM clones, *hsFLP tubGAL80 FRT19A/FRT19A; UASmCD8GFP/+; repoGAL4/+*. (C,D) BrdU, *tubGAL80^ts^/+; repoGAL4/UAScycE*.
(TIF)

Figure S4 Stabbing injury induces the incorporation of BrdU in IG adjacent to the lesion site. BrdU pulse experiment whereby dissected VNCs are cultured in BrdU during a 6 h pulse, and then they are fixed and labeled with antibodies. BrdU is incorporated during S-phase of dividing cells. Stabbing injury induces the incorporation of BrdU in Ebony+ IG adjacent to the lesion site, as the number of Ebony+ BrdU+ IG increases significantly compared to non-stabbed controls (*p*<0.05, Table S1). The wound boundary is indicated by a dashed line; Ebony+ BrdU+ IG are circled in the BrdU channel image. This is a high magnification view of the lesion site. Genotype: wild-type (wt): *yw*.
(TIF)

Figure S5 How DeadEasy larval glia works. To count the number of Repo-positive glial cells, we purposely developed a software program, called DeadEasy Larval Glia. It identifies the larval Repo-positive cells, acquired as confocal microscopy images, and counts them automatically throughout the stack of images in 3D. (A) The VNC is stabbed and labeled with anti-Repo, and a stack of images through the whole thickness of the abdomen (boxed Region of Interest, ROI) is acquired by laser scanning confocal microscopy. (B) DeadEasy processes each confocal slice independently to differentiate signal from noise and identify the stained objects, and it creates a stack of processed slices where the identified objects are in the same locations as the cells in the raw images. (C,Ci) Higher magnification examples showing the cells identified by DeadEasy (Ci) compared to the original raw image (C). (D) The programme is validated by creating a merged stack (colocalising cells appear white) from the stack of raw images (magenta) and the stack of processed images (green). The non-colocalising cells reveal the false positives and false negatives. (E) The objects identified in 2-D are analysed in 3-D. Those with a shape different from a circle or ellipse in 2-D, and below a minimum volume in 3-D, or intensity are ignored.
(TIF)

Figure S6 Ebony is a downstream differentiation target of Pros, but *pros* mutations do not cause major developmental defects in glia. (A) IG have both anti-Pros and anti-Ebony. Here the most ventral sections of the VNC have been eliminated since the neuroblast distribution of Pros would obscure the IG. (B) In *pros^voila1^/pros^S04116^* mutant larvae Ebony is missing, or its levels are reduced correlating with the residual levels of Pros in this hypomorphic allelic combination, compared to wild-type Ebony in (A). (C,D) The levels of Repo and GS2, and the number of glial cells, appear normal in *pros^voila1^/pros^S044116^* mutant larvae. The most relevant single channels are shown in greyscale. (A,C,D) are VNCs from wandering stage larvae and (B) from 96 h AEL VNCs, and all are projections of confocal images through the neuropile, longitudinal views on the left, transverse sections on the right (A–C).
(TIF)

Figure S7 IG loss upon stabbing in pros mutant larvae. (A) Injury in pros mutants causes the loss of Interface Glia, visualised by co-localisation of anti-GFP and Repo. Abundant small spots (arrow) are most likely apoptotic nuclei or apoptotic bodies. (B) IG may die of apoptosis, since Repo+ GFP+ IG nuclei can be surrounded by the cytoplasmic apoptotic marker cleaved-Caspase-3 (arrows). Feathered arrowhead indicates that the small GFP+ spots are apoptotic bodies since they are also Caspase+.
(TIF)

Figure S8 Expression of *egr/TNF, wgn/TNFR,* and *dorsal/NFkB* in the larval VNC. In situ hybridisations showing the distribution of *egr* (A,B) and *wgn* (C,D) transcripts in the larval ventral nerve cord (VNC) and brain. Both genes are expressed in the VNC, at higher levels in the thorax. Intense signal for *egr* and *wgn* transcripts is observed upon stabbing (arrows, B, B′, D–Dii), and of *wgn* in cells surrounding the neuropile, which are likely to be glial cells (arrows, D, Di) based on their location. (Bi,D, Dii) are higher magnification images than (A,B,C) and (Di) is higher magnification than (D); (Bi) is the same specimen as (B); (Dii) is a different specimen from (D and Di). (A, C, D, Di) are dorsal longitudinal views, (B, Bi, Dii) are ventral longitudinal views. Ml, midline. (E) IG express Dorsal as revealed by colocalisation of anti-Dorsal, anti-Ebony, and anti-βgal in IG in larvae bearing the *pros-lacZ* reporter, at the wandering stage. Transverse views.
(TIF)

Figure S9 Overall normal glial patterns in *egr* and *dorsal* mutants. Although there were rather subtle disorganization and slight changes in vIG number in *dorsal^H^/dorsal^1^* and *egr^1^/egr^3^* mutant larvae (unpublished data), total glial number (see Figure 8C) and the distribution of the two glial markers GS2 and Ebony were normal in both mutants. This shows that *dorsal^H^/dorsal^1^* and *egr^1^* mutations do not have dramatic consequences in glial development. Single channels are shown in greyscale. These are VNCs from 96 h AEL old larvae. Anterior is up.
(TIF)

Figure S10 Pros inhibits and Notch promotes cell division. (A) Loss of *pros* function affected the timing of glial cell division. In *pros^voila1^/pros^S044116^* hypomorphic mutant larvae, there were more glial cells in younger larvae (at 96 h AEL) than in wild-type; but in older, wandering larvae (120 h AEL), the number of glial cells in *pros^voila1^/pros^S044116^* mutants was indistinguishable from wild-type. This might suggest that the supernumerary glia in younger *pros* mutant larvae corresponded to faster (but not more) dividing glial precursors. (B) To test this, we expressed constitutively active *Rbf* in glia in *pros^S044116^* mutants, which would prevent the G1/S transition lengthening the G1 phase. This restored normal glial number in the younger larvae, meaning that the excess of glial cells in the mutant younger larvae arose from faster (but not more) glial divisions. (C) We were not able to detect CycE. Nevertheless, over-expression of *pros* in larval glia induced the expression of the CycE inhibitor Dacapo (Dap), the Drosophila p21/p27 homologue. Since Dap inhibits CycE, this is consistent with *pros* inhibiting *cycE* in larval glia. (D,E) Over-expression of *Notch^ICD^* in larvae upregulates *PCNA-GFP* in Ebony+ (D) and Repo+ (E) IG compared to non-heat-shocked controls. Colocalising signal is cytoplasmic, in white (top images). Larvae were heat-shocked at mid-third instar larvae and fixed 9 h later. (F,G) Over-expression of *Notch^ICD^* in larvae induces the incorporation of BrdU in Ebony+ IG. (H) Diagram of the involvement in the regulation of cell cycle progression by Notch, Pros, PCNA, and Dap. *** *p*<0.001; * *p*<0.05. Genotypes: (A) (1) wild-type = *yw*; (2) *pros^voila^/pros^S044116^*; (B) (1) wild-type = *yw*; (2) *pros^S044116^*; (3) *repoGAL4 pros^S044116^/pros^S044116^UASrbf280*; (C) (1) Control: *tubGAL80^ts^/+; repoGAL4/+;* (2) *tubGAL80^ts^/UASpros; repoGAL4/+;* (D) (1) Control: *PCNA-GFP/+;;UASNotch^ICD^myc/hsGAL4* no heat-shock; (2) *PCNA-GFP/+;;UASNotch^ICD^myc/hsGAL4* with heat-shock. (E) (1) Control: *hsGAL4/+*; (2) *hsGAL4/UASNotch^ICD^myc*.
(TIF)

Figure S11 Reciprocal maintenance of Pros and Notch in IG. (A) The Notch ligand Serrate (Ser) is distributed in VNC axons. Longitudinal confocal projection. Nuclei are labelled with Daunomycin; cross-section views on the right. (B) Notch signalling is activated in IG, as revealed by expression of the *Su(H)lacZ* reporter and anti-βgal in non-neuronal cells, in wild-type larval VNCs. Nuclear anti-Pros is surrounded by cytoplasmic anti-bgal in Elav negative cells (arrowheads). (C) Notch signalling is activated in IG, as revealed by colocalisation of anti-βgal and anti-Ebony in IG in wild-type larvae bearing the *Su(H)lacZ* reporter (arrowhead); cross-section views below. (D) Notch signalling is reduced in *Su(H)lacZ; pros^voila1^/pros^S044116^* mutants, as seen by low anti-βgal levels, together with loss of Ebony (arrowhead). This shows that Pros positively regulates Notch in IG. (E) Pros and its downstream target Ebony are downregulated upon expression of the Notch antagonist *numb*, and upregulated upon expression of *Notch^ICD^*, in all glia. (F) Quantification of data in (E) showing that the number of Ebony+ IG was reduced in *pros* mutants due to the downregulation of Ebony expression, and it increased upon activation of Notch in glia. Box plots illustrate sample distribution: line across is the median, the box corresponds to 50% samples at either side of the median, and wiskers correspond to 25% sample quartiles. Numbers over box-plots *n* = number of VNCs analysed, ** *p*<0.01. (G) Summary of Pros-Notch feedback by which Pros and Notch maintain each other. Genotypes from left to right: (D) *Su(H)lacZ; pros^voila1^/pros^S044116^*; (E) All glia stands for repoGAL4: (1) *repoGAL4/UASnumb*; (2) *repoGAL4/UASNotch^ICD^myc*; (F) (1) *pros^voila1^/pros^S044116^*; (2) *repoGAL4/UASnumb*; (3) *repoGAL4/UASNotch^ICD^myc*.
(TIF)

Figure S12 No abdominal neuroblasts proliferate in *pros* mutants over-expressing Notch^ICD^ in glia. (A, C) The colocalisation of the mitotic marker anti-phospho-Histone-H3 (pH3) and the neuroblast marker anti-Miranda (Mira) is restritcted to the thorax in wild-type (A) and *pros*- (C) mutant larvae. (B) There is no colocalisation of pH3 and Mira in the abdomen of *pros* mutant VNCs over-expressing *Notch^ICD^* in glia. pH3+ and Mira-negative spots in the abdomen correspond to glia, as shown in Figure 5B of the main manuscript, as they colocalise with the glial marker Repo. Genotypes: (A) wild-type = *yw*; (B) *pros^S044116^repoGAL4/pros^S044116^UASNotch^ICD^myc*; (C) *pros^voila1^/pros^S044116^*.
(TIF)

Figure S13 Normal HB9 and FasII patterns in *pros* mutants and in *pros* mutants expressing *Notch^ICD^* in glia. Confocal longitudinal projections of larvae labelled with the neuronal markers (A,Ai) nuclear anti-HB9 and (B) axonal FasII. (A,Ai) The number of HB9 neurons is virtually the same in the three different genotypes. (B) The three major, dorsal FasII fascicles appear normal in the three different genotypes (arrowheads). These are VNC from wandering stage larvae. (Ai,Bi) are higher magnification detail of (A,B). Anterior is up; ml, midline. Genotypes: wild-type = *yw*; *pros* mutant: *pros^S044116^/pros^S044116^*; glia>NICD in pros- = *pros^S044116^repoGAL4/UASNotch^ICD^myc pros^S044116^*.
(TIF)

Figure S14 Over-expression of *Notch^ICD^* does not induce GS2 expression in non-enwrapping glia. (A,D) Horizontal views of the abdominal VNC showing that GS2 expression is restricted to enwrapping glia and does not coincide with repoGAL4>mCD8GFP except for the neuropile and nerves exiting the CNS. (C,D) Transverse views showing that while GFP is present throughout the cortex, there is no GS2 expression outside the neuropile. Note also that *Notch^ICD^* upregulates GFP predominantly within the neuropile. Genotypes: (A,B) Control: *w; UASmCD8GFP/+; repoGAL4/+*; (C,D) *w; UASmCD8GFP/+; repoGAL4/UASNotch^ICD^myc*.
(TIF)

Figure S15 Over-expression of *dTRAF2* induces glial proliferation. To test whether activation of Dorsal/NFkB can induce cell cycle progression, we induced a pulse of *dTRAF2* expression at the mid-third instar stage using *hsGAL4*, during which larvae were fed with BrdU for a 6 h period. (A) Compared to controls, the number of Ebony+ IG cells incorporating BrdU increased significantly in larvae over-expressing *dTRAF2*, quantification in (B), showing that expression of *dTRAF2* induces the G1-S transition in glia. Larvae were heat-shocked at 96 h AEL, treated with a BrdU pulse after 6 h, fixed 3 h later. Genotypes: (1) *hsGAL4/+*; (2) *UASdTRAF2/+;;hsGAL4/+*.
(TIF)

Table S1 Statistical analysis: Sample sizes, tests and p values.
(PDF)

Text S1 Evidence that IG do not normally divide but are arrested in G1 and can be induced to divide.
(DOC)

Text S2 Evidence that the increase in glial number is not due to neuroblast divisions.
(DOC)

Text S3 Detailed methods.
(DOC)

Video S1 Temporal progression of injury in wild-type. The wound first expands and vacuoles form in the axonal neuropile. A wild-type VNC was filmed from 0 to 13 h after stabbing. Glial cells were labelled with DsRed and all CNS axons with the axonal protein-trap line G9-GFP. Longitudinal view, anterior is up; merge is shown in colour, individual channels in greyscale. This video is from a different specimen from that shown in Figure 1B. Single frames for this video are shown in Figure 10A. Genotype: *UASDsRed/+;G9/+;repoGAL4/+*.
(MOV)

Video S2 Correlation between healing of vacuoles and presence of glia. In wild-type stabbed VNCs, some vacuoles or holes are healed, and these are frequently filled with glial cells prior to repair. Time-lapse video of a wild-type VNC filmed from 6 to 20 h after stabbing injury. Glial cells are labelled with DsRed, and all CNS axons are labelled with the axonal protein-trap line G9-GFP. Longitudinal view, anterior is up; merge is shown in colour, individual channels in greyscale. This video is from a different specimen from that shown in Figure 1B. Single frames for this video are shown in Figure 10B. Genotype: *UASDsRed/+;G9/+;repoGAL4/+*.
(MOV)

Video S3 3-D visualisation of a control non-stabbed VNC. 3-D rendering animation of a wild-type VNC expressing the glial reporter *repoGAL4>UASmCD8GFP*, fixed and labelled with anti-GFP to visualise all glia, and with anti-GS2 for the IG enwrapping the axonal neuropile. The GFP channel is switched off for part of the movie to highlight the GS+ enwrapping glia. Snapshots of this video are shown in Figure S1.
(MOV)

Video S4 3-D visualisation of a stabbed VNC. The arrow shows the entry dorsal stabbing site and as the video turns a ventral lesion. At the lesion site (asterisk), GS2+ IG were lost. The VNC was labelled with *repoGAL4>UASmCD8GFP*, anti-GFP, and anti-GS2. Snapshots are shown in Figure S1.
(MOV)

Video S5 The injury wound does not contract but expands in *Notch^ts1^* mutant larvae. The wound expands, and abundant vacuoles form in the axonal neuropile in *Notch^ts1^* mutants. Time-lapse video of a *Notch^ts1^* mutant VNC filmed from 7 to 17 h after stabbing. Glial cells are labelled with DsRed, and all CNS axons are labelled with the axonal protein-trap line G9-GFP. Single frames are shown in Figure 10C. Genotype: *UASDsRed Notch^ts1^/Y; G9/+; repoGAL4/*+.
(MOV)

Video S6 The wound area expands upon expression of *pros* in IG. Wound area and vacuolisation increase when glial proliferation is inhibited upon the over-expression of *pros* in IG. Time-lapse video of a VNC expressing *pros* in IG filmed from 7 to 15 h after stabbing injury. IG cells are labelled with DsRed and all CNS axons are labelled with the axonal protein-trap line G9-GFP. Single frames are shown in Figure 10D. Genotype: *UASDsRed/+;G9/UASpros; alrmGAL4/+*.
(MOV)

Video S7 Temporal progression of injury upon expression of *Notch^ICD^* in glia. When glial proliferation is induced by expressing *Notch^ICD^* in glia, the wound does not expand as it did in wild-type, and there is complete axonal repair in this specimen by 13 h. Time-lapse video of a VNC expressing *Notch^ICD^* in glia filmed from 0 to 21 h after stabbing injury. Glial cells are labelled with DsRed, and all CNS axons are labelled with the axonal protein-trap line G9-GFP. Longitudinal view, anterior is up; merge is shown in colour, individual channels in greyscale. Single frames are shown in Figure 10E. Genotype: *UASDsRed/+;G9/+:repoGAL4/UASNotch^ICD^myc*.
(MOV)

## Abbreviations

AEL: after egg laying
BrdU: bromodeoxyuridine
CNS: central nervous system
CyCE: cyclin E
Dap: Dacapo
dl: dorsal
Egr: Eiger
FasII: fasciclin 2
GFP: green fluorescent protein
GS2: glutamine Synthetase-2
IG: interface glia
JNK: c-Jun N-terminal kinase
MARCM: mosaic analysis with a repressive cell marker
Mira: Miranda
NFκB: nuclear factor-kappa B
Notch^ICD^ and NICD: Notch Intracellular domain
OPC: Oligodendrocyte progenitor cells
PCNA: proliferating cell nuclear antigen
PH3: Phospho-Histone H3
Pros: Prospero
Rbf: Retinoblastoma-family protein
TNF: Tumor necrosis factor
TNFR: Tumor necrosis factor receptor
VNC: ventral nerve cord
Wgn: Wengen
YFP: Yellow Fluorescent Protein
