# Supplementary figures and images for: The Glial Regenerative Response to Central Nervous System Injury Is Enabled by Pros-Notch and Pros-NFκB Feedback
Source: PLoS Biol. 2011 Aug 30;9(8):e1001133. doi: 10.1371/journal.pbio.1001133 (PMC3166069; doi:10.1371/journal.pbio.1001133)

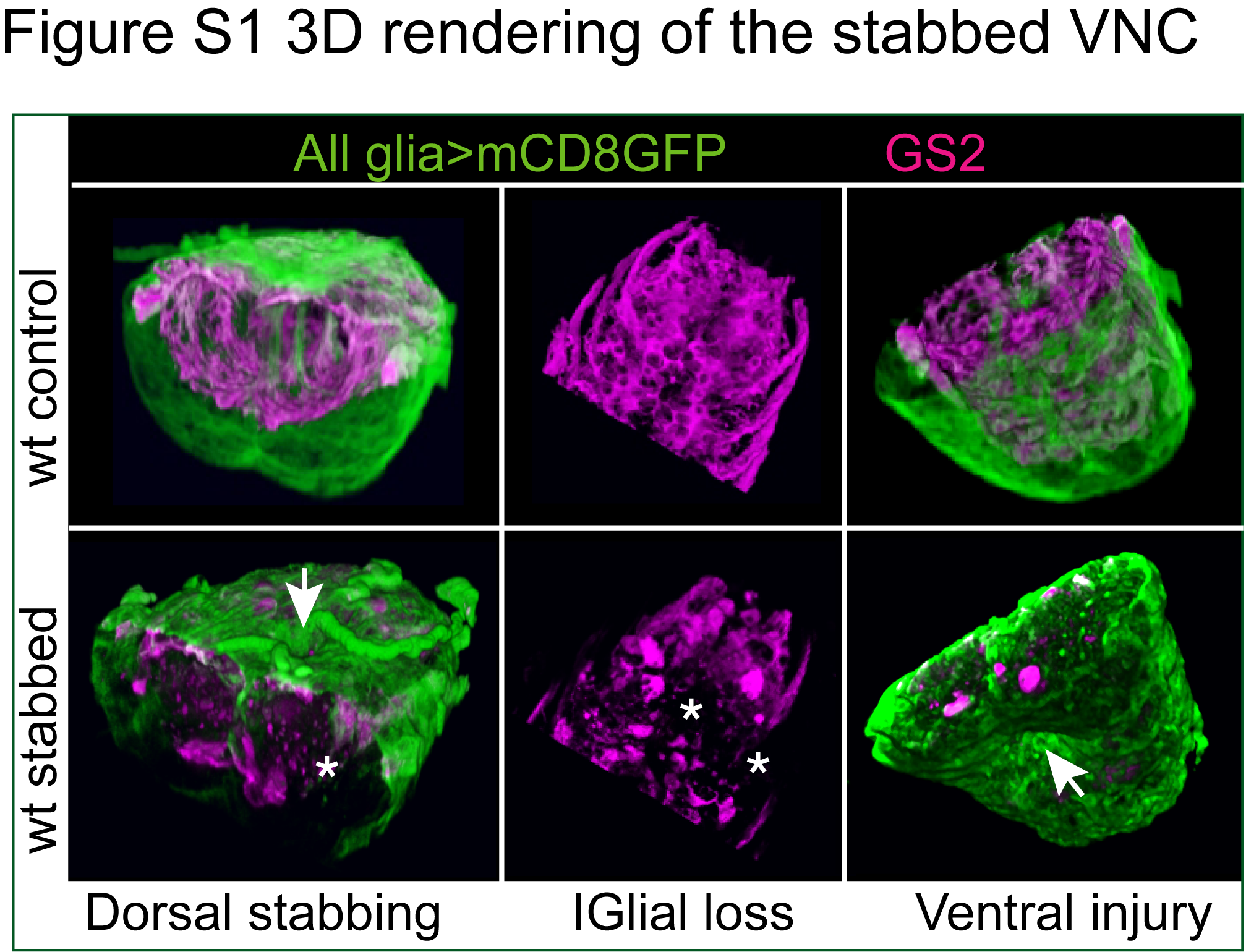

Supplement: Figure S1 — 3-D rendering of the stabbed Ventral Nerve Cord. Snapshots from Videos S3 and S4 showing that stabbing creates an injury at the dorsal entry site and a secondary injury ventrally (arrows), and results in GS2+ IG loss (asterisks). (TIF) [file pbio.1001133.s001.tif]

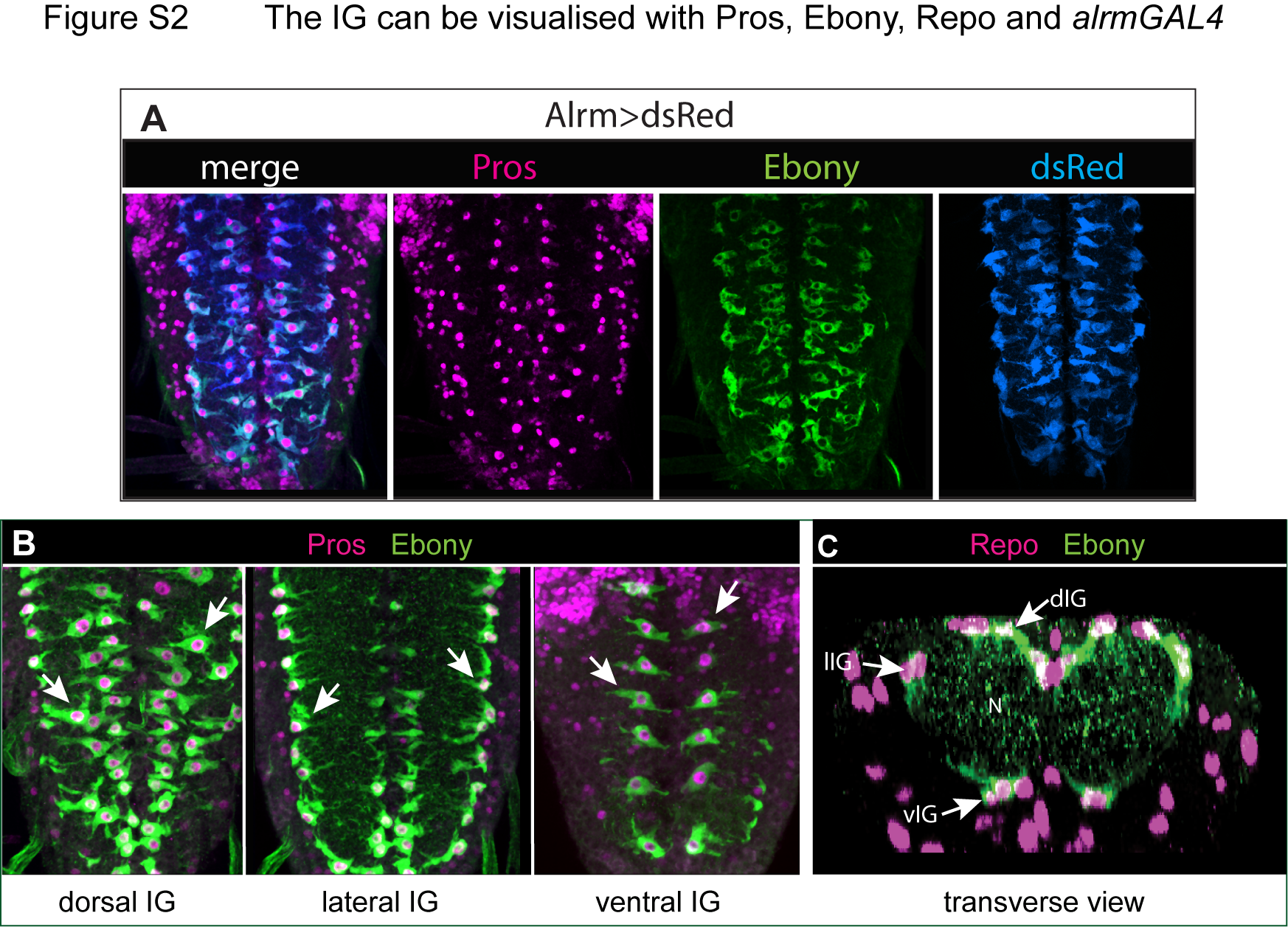

Supplement: Figure S2 — The IG can be visualised with Pros, Ebony, Repo, and alrmGAL4. (A) AlrmGAL4>dsRed drives expression in Pros+ Interface Glia as seen by colocalisation of dsRed, Pros, and Ebony in these cells. (B) Confocal longitudinal sections through different focal planes in the VNC, from dorsal to ventral, to show the IG sub-types. IG co-express the nuclear transcription factor Pros and cytoplasmic Ebony involved in neurotransmitter recycling (arrows). (C) Tranverse view showing colocalisation of Ebony and Repo in IG (arrows) around the axonal neuropile (N); the IG nuclei are located outside, glial processes project into the neuropile. dIG, dorsal Interface Glia; lIG, lateral Iterface Glia; vIG, ventral Interface Glia. Genotype for (A): UASDsRed/+; alrmGAL4/+. (TIF) [file pbio.1001133.s002.tif]

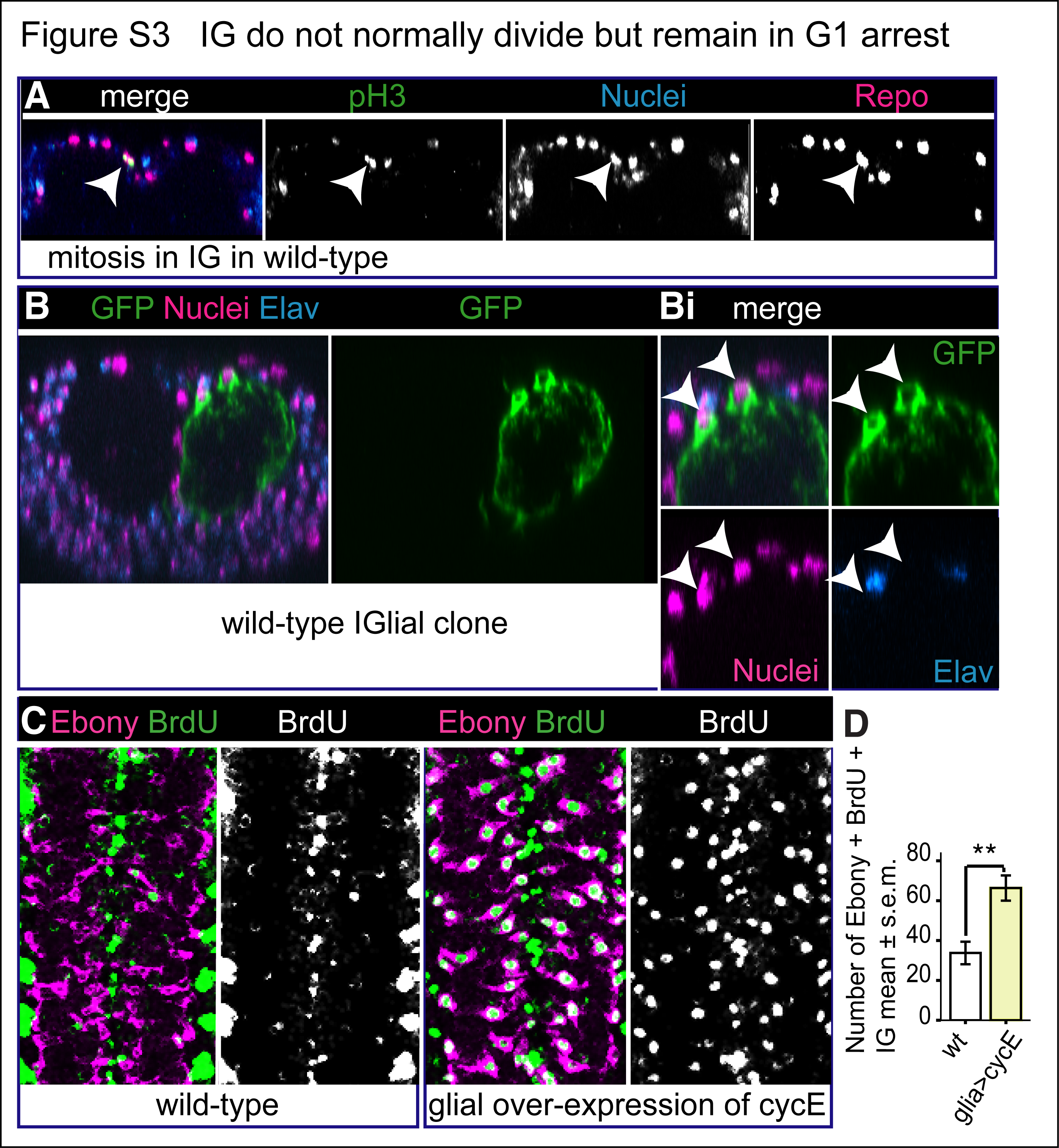

Supplement: Figure S3 — IG do not normally divide but remain in G1 arrest. (A,B) The mitotic marker anti-phospho-Histone-H3 (pH3) and MARCM clonal analysis show that the incidence of IG division is rather low in wild-type non-stabbed larval VNCs, meaning that generally IG do not divide. (A) Proliferation of IG could be seen in wild-type larvae by colocalisation of the glial marker anti-Repo and pH3 (arrowhead) at the mid-third instar stage, but only 0.6% VNCs had one mitotic IG (n = 181 VNCs), whereas 44% VNCs had at least one pH3 positive glial cell including other glial types. Confocal transverse views through the dorsal half of the VNC neuropile and the neuropile edge is revealed with the nuclear die DAPI (blue). (B, Bi higher magnification) GFP-labelled MARCM clones were induced by heat-shock at the early third instar stage and specimens were fixed at the wandering stage. The GAL4-dependent expression of GFP is repressed by the inhibitor GAL80 and repression is relieved when mitotic recombination is induced by hs-FLIPase. Thus, presence of GFP in IG implies that mitosis occurred. Here a two-cell clone is shown, and cytoplasmic projections enwrap the neuropile and project within. IG clones are identified by the exclusion of the neuronal marker Elav (arrowheads) and their enwrapment of the neuropile; the nuclear dye is DAPI. 15 wild-type VNCs had one IG clone; n = 1,254 VNCs were analysed. (C) To test whether glial cells were arrested with mitotic potential, we asked whether transient expression of cycE in larval glia resulted in an increase in IG entering S-phase as measured with a brief BrdU pulse. Using temperature sensitive GAL80ts, larvae were shifted at the restricted temperature and treated with BrdU for 6 h. Transient expression of cycE in glia at the mid-third instar larval stage resulted in a significant increase in Ebony+ IG that incorporated BrdU compared to wild-type controls. These data show that in the normal larval CNS, IG are mostly arrested, have not terminally exited t [file pbio.1001133.s003.tif]

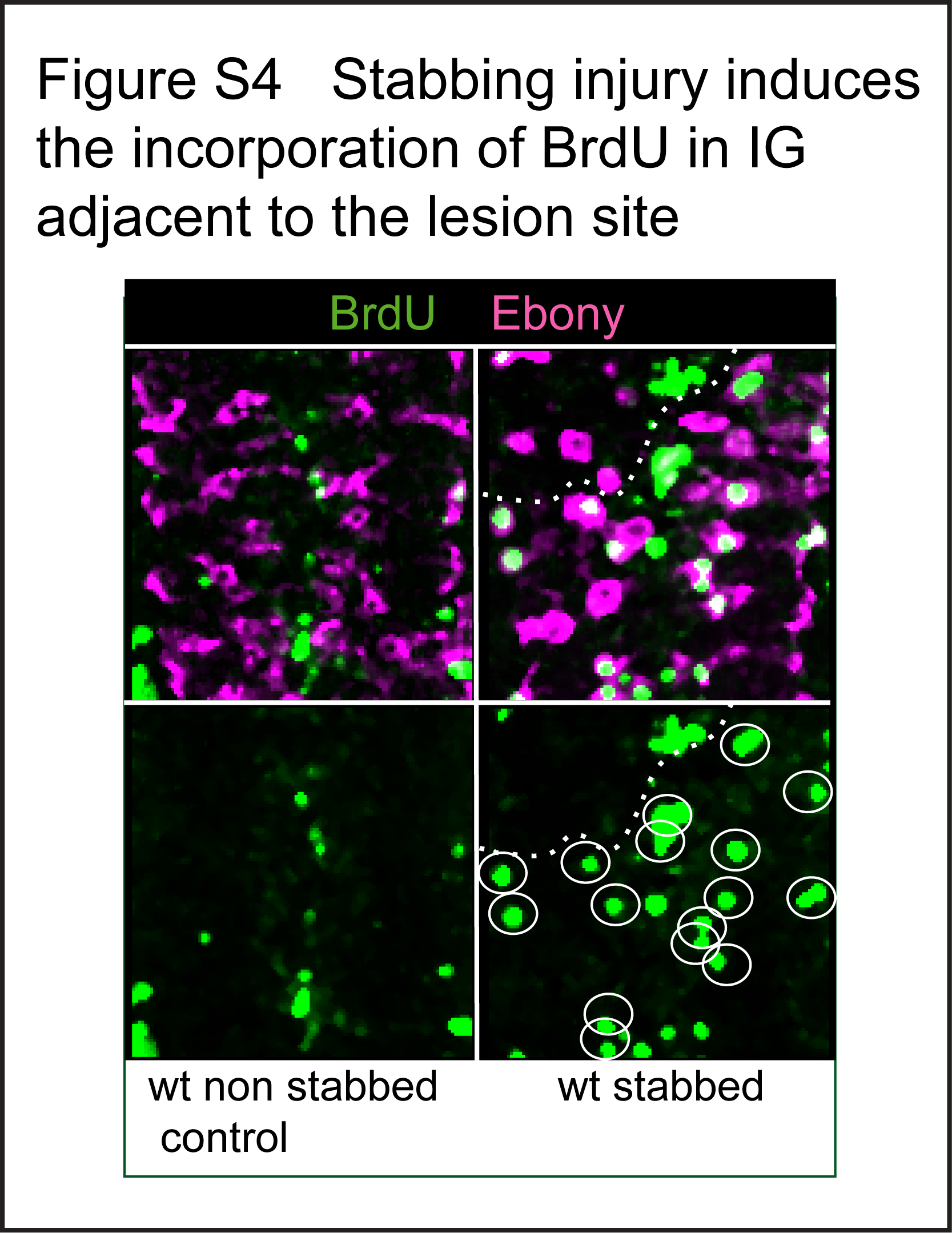

Supplement: Figure S4 — Stabbing injury induces the incorporation of BrdU in IG adjacent to the lesion site. BrdU pulse experiment whereby dissected VNCs are cultured in BrdU during a 6 h pulse, and then they are fixed and labeled with antibodies. BrdU is incorporated during S-phase of dividing cells. Stabbing injury induces the incorporation of BrdU in Ebony+ IG adjacent to the lesion site, as the number of Ebony+ BrdU+ IG increases significantly compared to non-stabbed controls (p<0.05, Table S1). The wound boundary is indicated by a dashed line; Ebony+ BrdU+ IG are circled in the BrdU channel image. This is a high magnification view of the lesion site. Genotype: wild-type (wt): yw. (TIF) [file pbio.1001133.s004.tif]

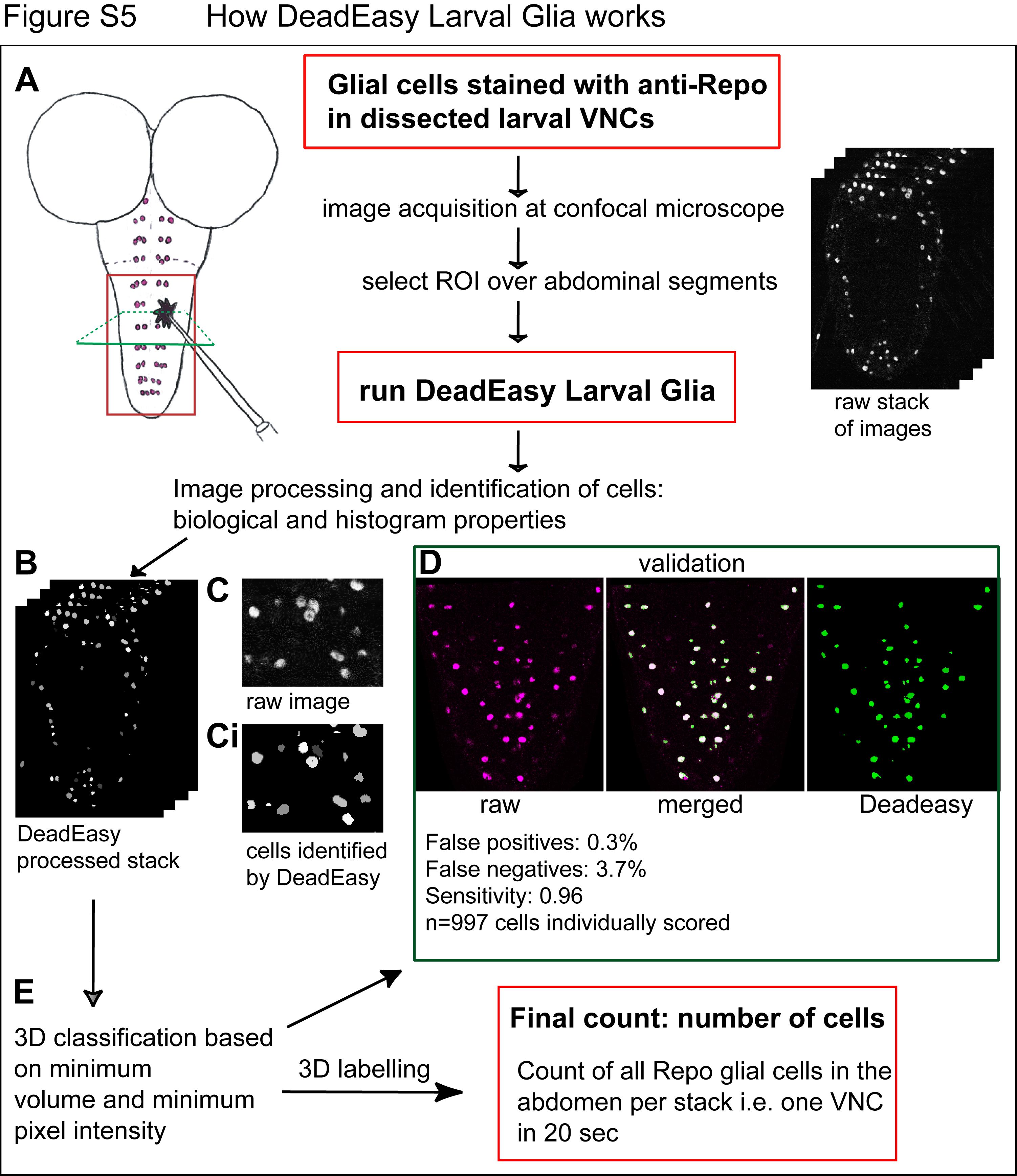

Supplement: Figure S5 — How DeadEasy larval glia works. To count the number of Repo-positive glial cells, we purposely developed a software program, called DeadEasy Larval Glia. It identifies the larval Repo-positive cells, acquired as confocal microscopy images, and counts them automatically throughout the stack of images in 3D. (A) The VNC is stabbed and labeled with anti-Repo, and a stack of images through the whole thickness of the abdomen (boxed Region of Interest, ROI) is acquired by laser scanning confocal microscopy. (B) DeadEasy processes each confocal slice independently to differentiate signal from noise and identify the stained objects, and it creates a stack of processed slices where the identified objects are in the same locations as the cells in the raw images. (C,Ci) Higher magnification examples showing the cells identified by DeadEasy (Ci) compared to the original raw image (C). (D) The programme is validated by creating a merged stack (colocalising cells appear white) from the stack of raw images (magenta) and the stack of processed images (green). The non-colocalising cells reveal the false positives and false negatives. (E) The objects identified in 2-D are analysed in 3-D. Those with a shape different from a circle or ellipse in 2-D, and below a minimum volume in 3-D, or intensity are ignored. (TIF) [file pbio.1001133.s005.tif]

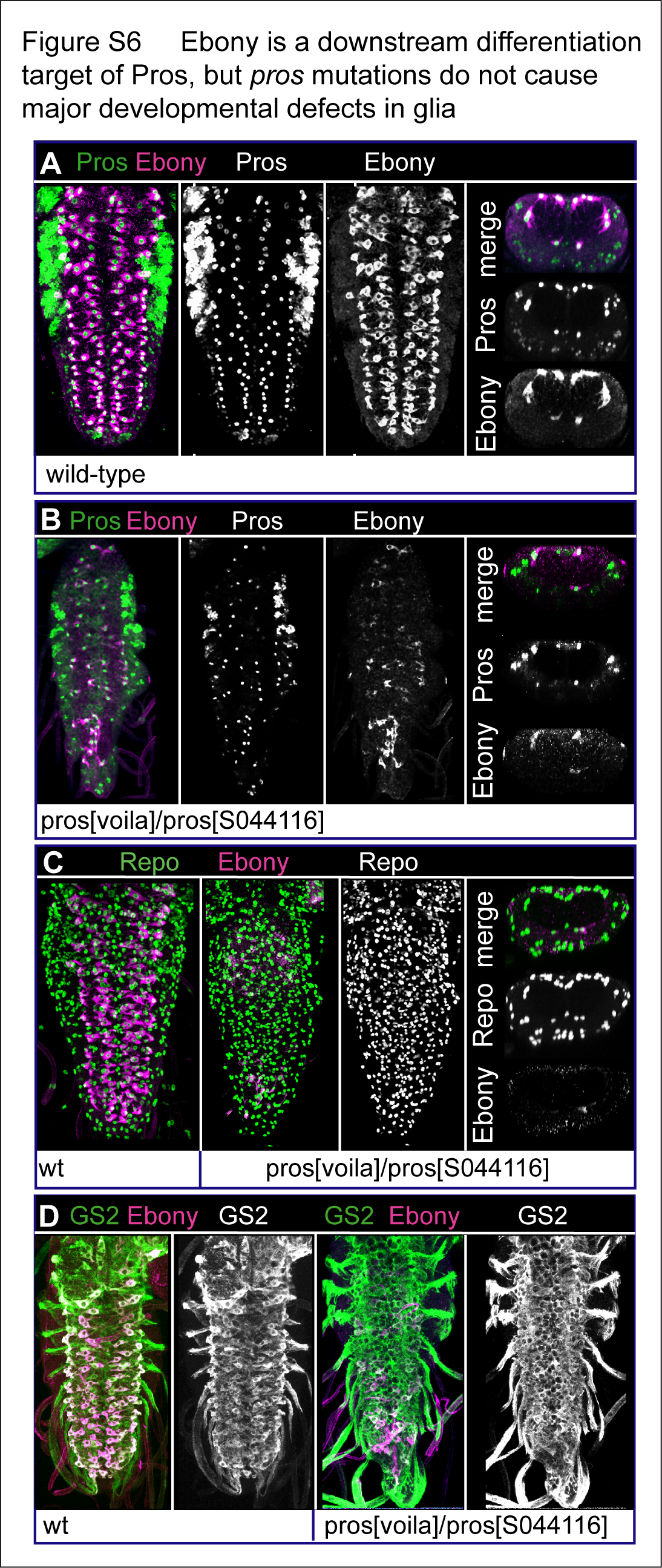

Supplement: Figure S6 — Ebony is a downstream differentiation target of Pros, but pros mutations do not cause major developmental defects in glia. (A) IG have both anti-Pros and anti-Ebony. Here the most ventral sections of the VNC have been eliminated since the neuroblast distribution of Pros would obscure the IG. (B) In prosvoila1/prosS04116 mutant larvae Ebony is missing, or its levels are reduced correlating with the residual levels of Pros in this hypomorphic allelic combination, compared to wild-type Ebony in (A). (C,D) The levels of Repo and GS2, and the number of glial cells, appear normal in prosvoila1/prosS044116 mutant larvae. The most relevant single channels are shown in greyscale. (A,C,D) are VNCs from wandering stage larvae and (B) from 96 h AEL VNCs, and all are projections of confocal images through the neuropile, longitudinal views on the left, transverse sections on the right (A–C). (TIF) [file pbio.1001133.s006.tif]

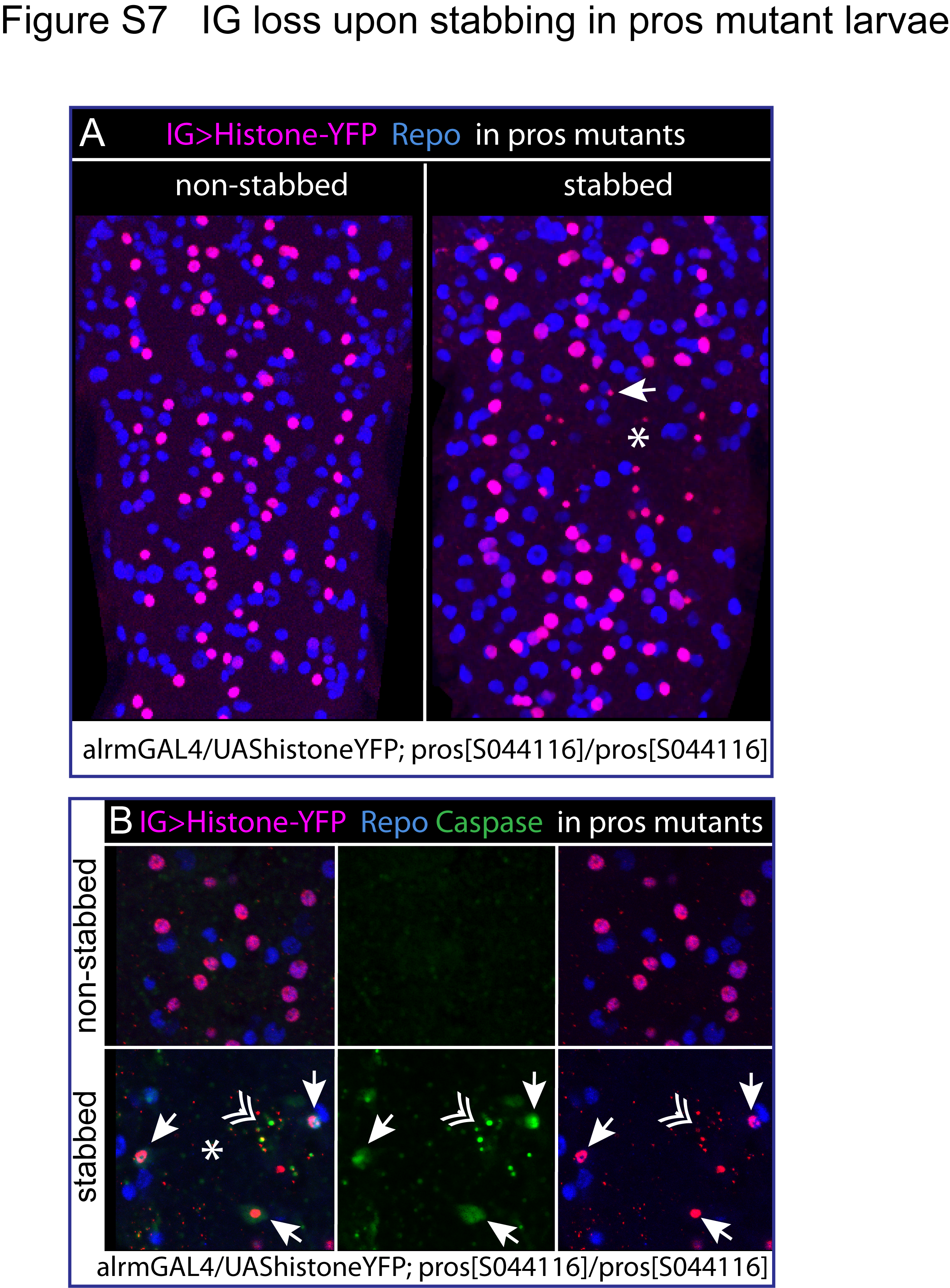

Supplement: Figure S7 — IG loss upon stabbing in pros mutant larvae. (A) Injury in pros mutants causes the loss of Interface Glia, visualised by co-localisation of anti-GFP and Repo. Abundant small spots (arrow) are most likely apoptotic nuclei or apoptotic bodies. (B) IG may die of apoptosis, since Repo+ GFP+ IG nuclei can be surrounded by the cytoplasmic apoptotic marker cleaved-Caspase-3 (arrows). Feathered arrowhead indicates that the small GFP+ spots are apoptotic bodies since they are also Caspase+. (TIF) [file pbio.1001133.s007.tif]

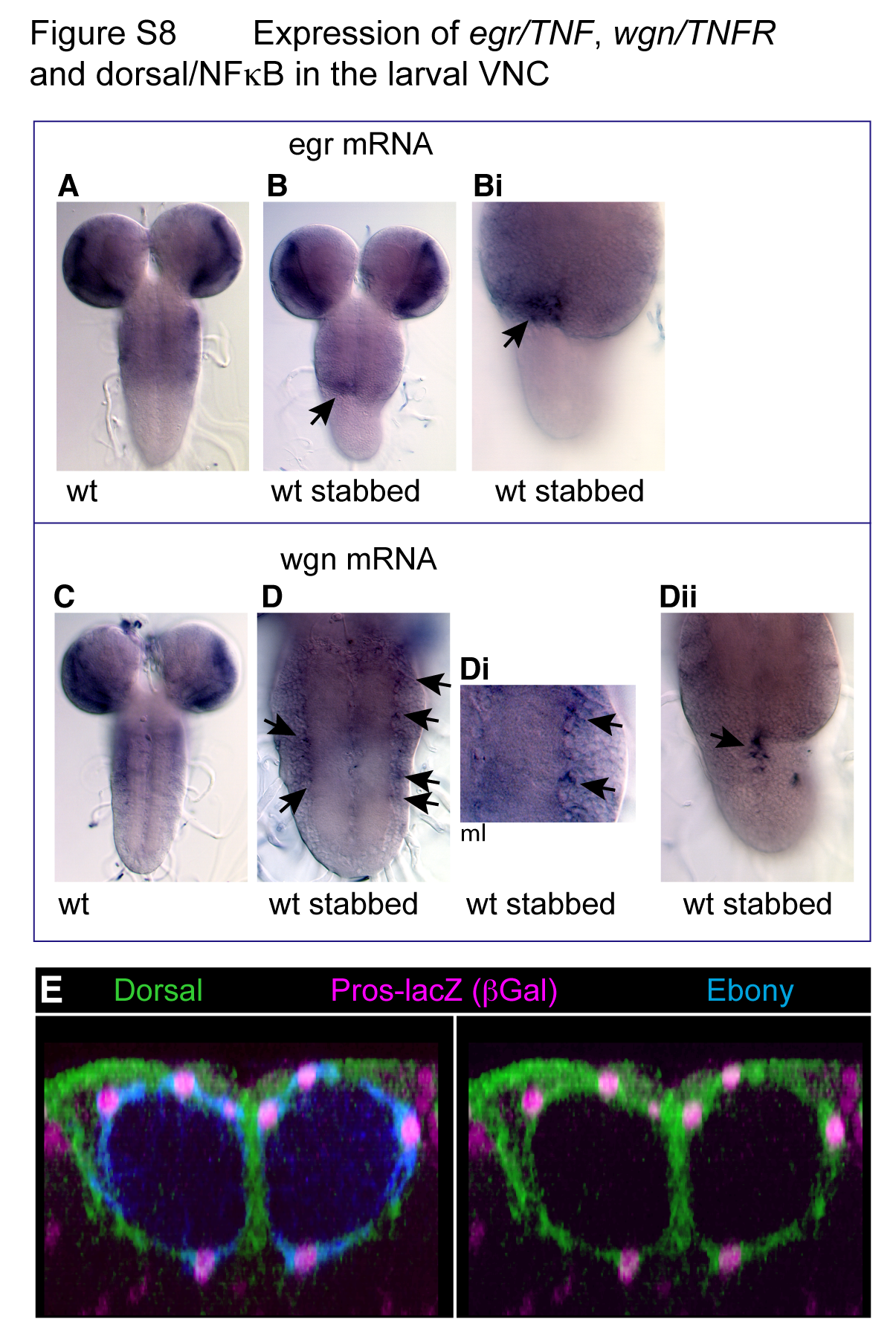

Supplement: Figure S8 — Expression of egr/TNF, wgn/TNFR, and dorsal/NFkB in the larval VNC. In situ hybridisations showing the distribution of egr (A,B) and wgn (C,D) transcripts in the larval ventral nerve cord (VNC) and brain. Both genes are expressed in the VNC, at higher levels in the thorax. Intense signal for egr and wgn transcripts is observed upon stabbing (arrows, B, B′, D–Dii), and of wgn in cells surrounding the neuropile, which are likely to be glial cells (arrows, D, Di) based on their location. (Bi,D, Dii) are higher magnification images than (A,B,C) and (Di) is higher magnification than (D); (Bi) is the same specimen as (B); (Dii) is a different specimen from (D and Di). (A, C, D, Di) are dorsal longitudinal views, (B, Bi, Dii) are ventral longitudinal views. Ml, midline. (E) IG express Dorsal as revealed by colocalisation of anti-Dorsal, anti-Ebony, and anti-βgal in IG in larvae bearing the pros-lacZ reporter, at the wandering stage. Transverse views. (TIF) [file pbio.1001133.s008.tif]

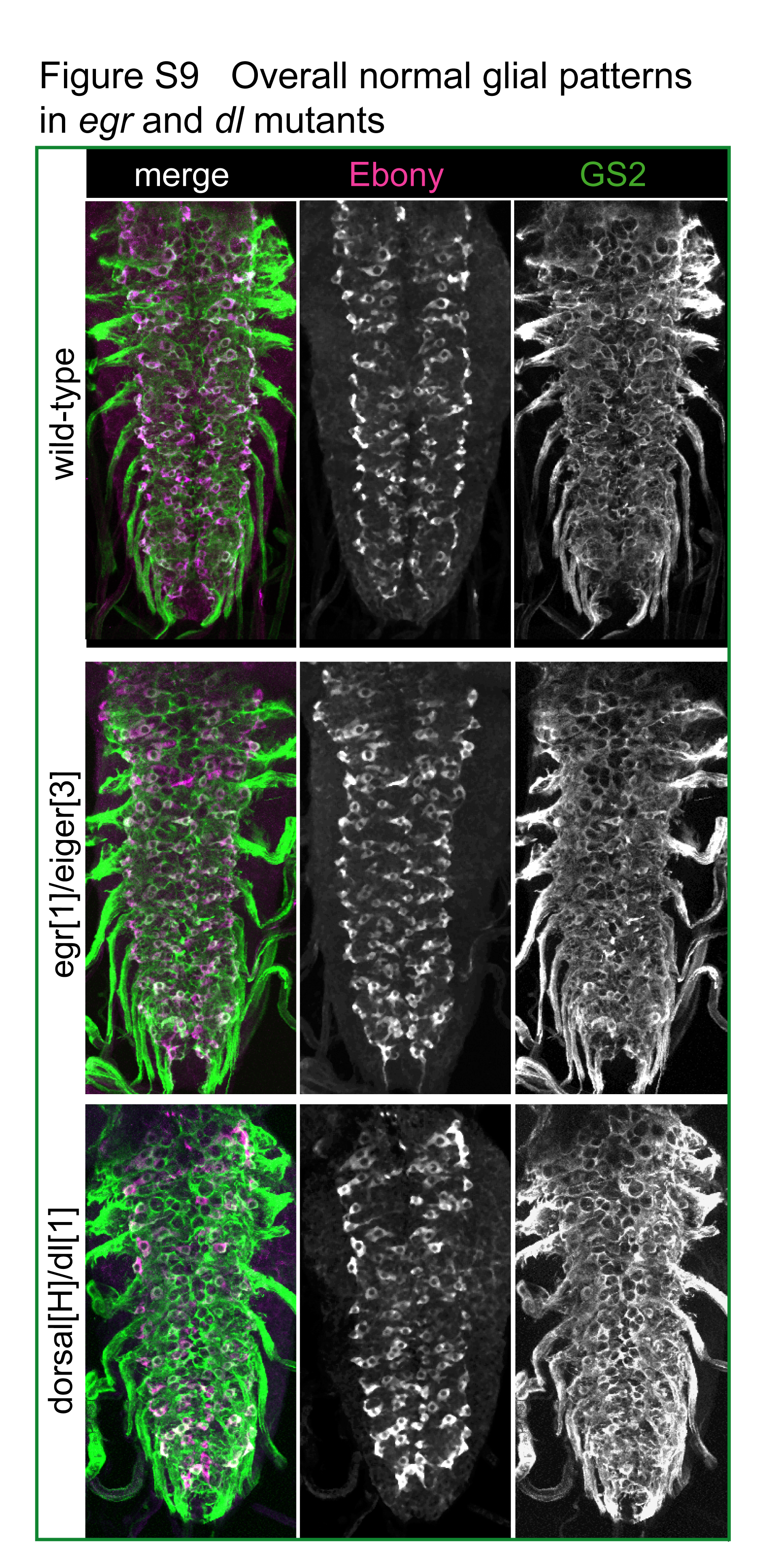

Supplement: Figure S9 — Overall normal glial patterns in egr and dorsal mutants. Although there were rather subtle disorganization and slight changes in vIG number in dorsalH/dorsal1 and egr1/egr3 mutant larvae (unpublished data), total glial number (see Figure 8C) and the distribution of the two glial markers GS2 and Ebony were normal in both mutants. This shows that dorsalH/dorsal1 and egr1 mutations do not have dramatic consequences in glial development. Single channels are shown in greyscale. These are VNCs from 96 h AEL old larvae. Anterior is up. (TIF) [file pbio.1001133.s009.tif]

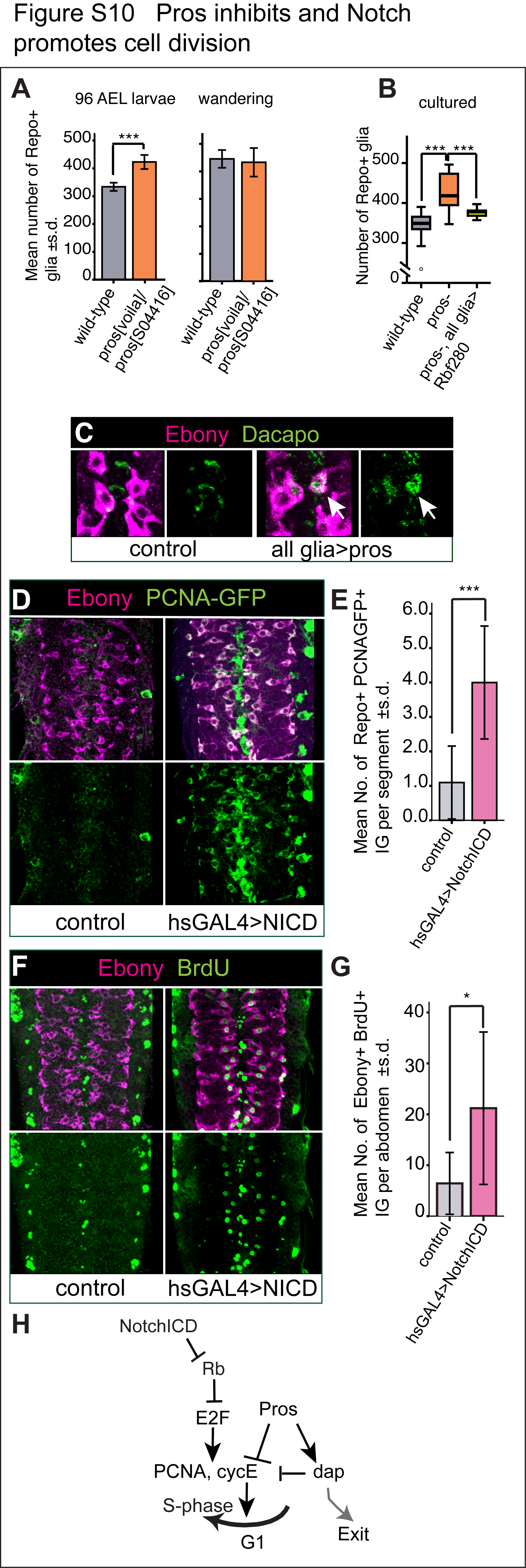

Supplement: Figure S10 — Pros inhibits and Notch promotes cell division. (A) Loss of pros function affected the timing of glial cell division. In prosvoila1/prosS044116 hypomorphic mutant larvae, there were more glial cells in younger larvae (at 96 h AEL) than in wild-type; but in older, wandering larvae (120 h AEL), the number of glial cells in prosvoila1/prosS044116 mutants was indistinguishable from wild-type. This might suggest that the supernumerary glia in younger pros mutant larvae corresponded to faster (but not more) dividing glial precursors. (B) To test this, we expressed constitutively active Rbf in glia in prosS044116 mutants, which would prevent the G1/S transition lengthening the G1 phase. This restored normal glial number in the younger larvae, meaning that the excess of glial cells in the mutant younger larvae arose from faster (but not more) glial divisions. (C) We were not able to detect CycE. Nevertheless, over-expression of pros in larval glia induced the expression of the CycE inhibitor Dacapo (Dap), the Drosophila p21/p27 homologue. Since Dap inhibits CycE, this is consistent with pros inhibiting cycE in larval glia. (D,E) Over-expression of NotchICD in larvae upregulates PCNA-GFP in Ebony+ (D) and Repo+ (E) IG compared to non-heat-shocked controls. Colocalising signal is cytoplasmic, in white (top images). Larvae were heat-shocked at mid-third instar larvae and fixed 9 h later. (F,G) Over-expression of NotchICD in larvae induces the incorporation of BrdU in Ebony+ IG. (H) Diagram of the involvement in the regulation of cell cycle progression by Notch, Pros, PCNA, and Dap. *** p<0.001; * p<0.05. Genotypes: (A) (1) wild-type = yw; (2) prosvoila/prosS044116; (B) (1) wild-type = yw; (2) prosS044116; (3) repoGAL4 prosS044116/prosS044116UASrbf280; (C) (1) Control: tubGAL80ts/+; repoGAL4/+; (2) tubGAL80ts/UASpros; repoGAL4/+; (D) (1) Control: PCNA-GFP/+;;UASNotchICDmyc/hsGAL4 no heat-shock; (2) PCNA-GFP/+;;UASNotchICDmyc/hsGAL4 with heat-shock. (E) (1) Control: hsGAL4/+; (2 [file pbio.1001133.s010.tif]

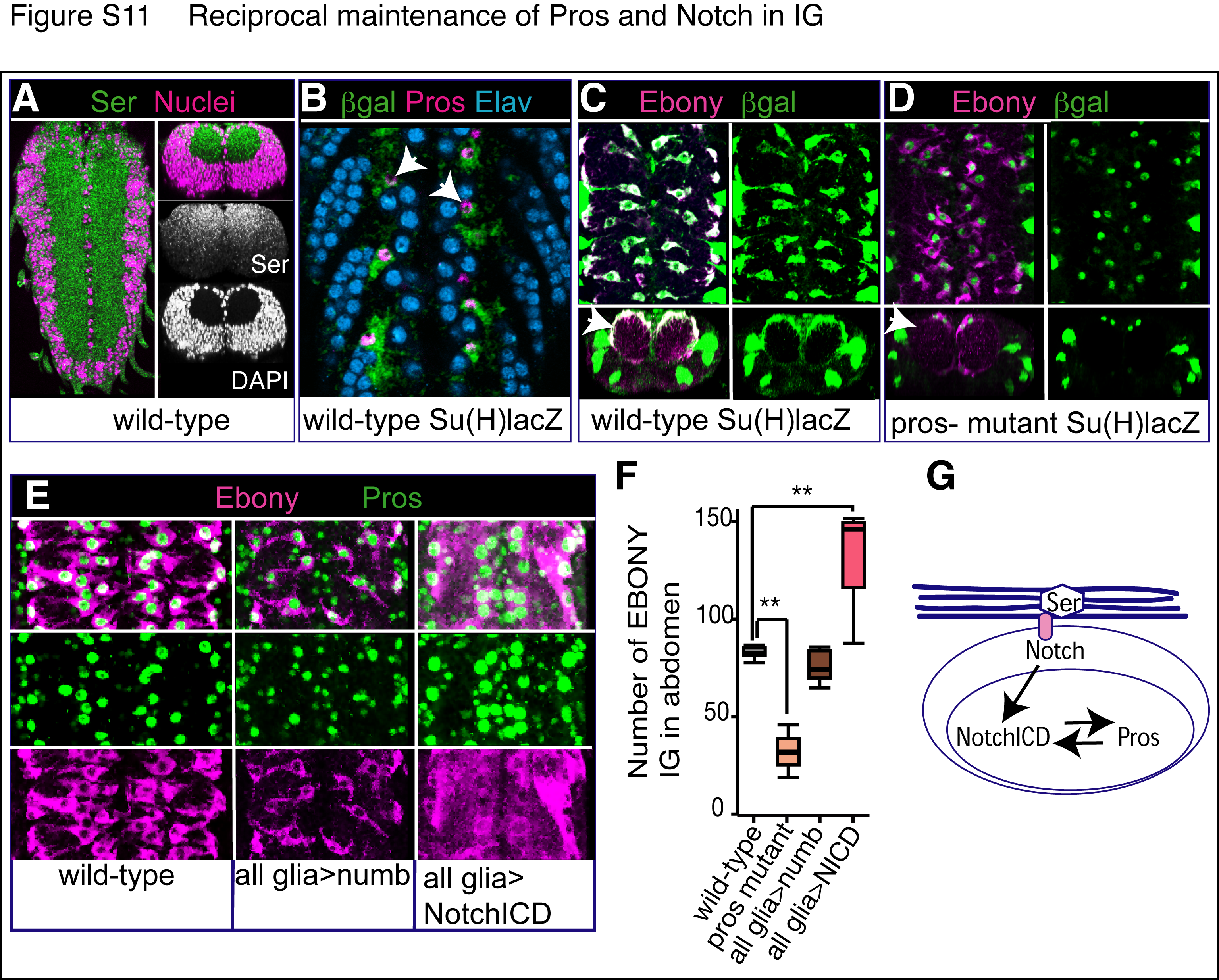

Supplement: Figure S11 — Reciprocal maintenance of Pros and Notch in IG. (A) The Notch ligand Serrate (Ser) is distributed in VNC axons. Longitudinal confocal projection. Nuclei are labelled with Daunomycin; cross-section views on the right. (B) Notch signalling is activated in IG, as revealed by expression of the Su(H)lacZ reporter and anti-βgal in non-neuronal cells, in wild-type larval VNCs. Nuclear anti-Pros is surrounded by cytoplasmic anti-bgal in Elav negative cells (arrowheads). (C) Notch signalling is activated in IG, as revealed by colocalisation of anti-βgal and anti-Ebony in IG in wild-type larvae bearing the Su(H)lacZ reporter (arrowhead); cross-section views below. (D) Notch signalling is reduced in Su(H)lacZ; prosvoila1/prosS044116 mutants, as seen by low anti-βgal levels, together with loss of Ebony (arrowhead). This shows that Pros positively regulates Notch in IG. (E) Pros and its downstream target Ebony are downregulated upon expression of the Notch antagonist numb, and upregulated upon expression of NotchICD, in all glia. (F) Quantification of data in (E) showing that the number of Ebony+ IG was reduced in pros mutants due to the downregulation of Ebony expression, and it increased upon activation of Notch in glia. Box plots illustrate sample distribution: line across is the median, the box corresponds to 50% samples at either side of the median, and wiskers correspond to 25% sample quartiles. Numbers over box-plots n = number of VNCs analysed, ** p<0.01. (G) Summary of Pros-Notch feedback by which Pros and Notch maintain each other. Genotypes from left to right: (D) Su(H)lacZ; prosvoila1/prosS044116; (E) All glia stands for repoGAL4: (1) repoGAL4/UASnumb; (2) repoGAL4/UASNotchICDmyc; (F) (1) prosvoila1/prosS044116; (2) repoGAL4/UASnumb; (3) repoGAL4/UASNotchICDmyc. (TIF) [file pbio.1001133.s011.tif]

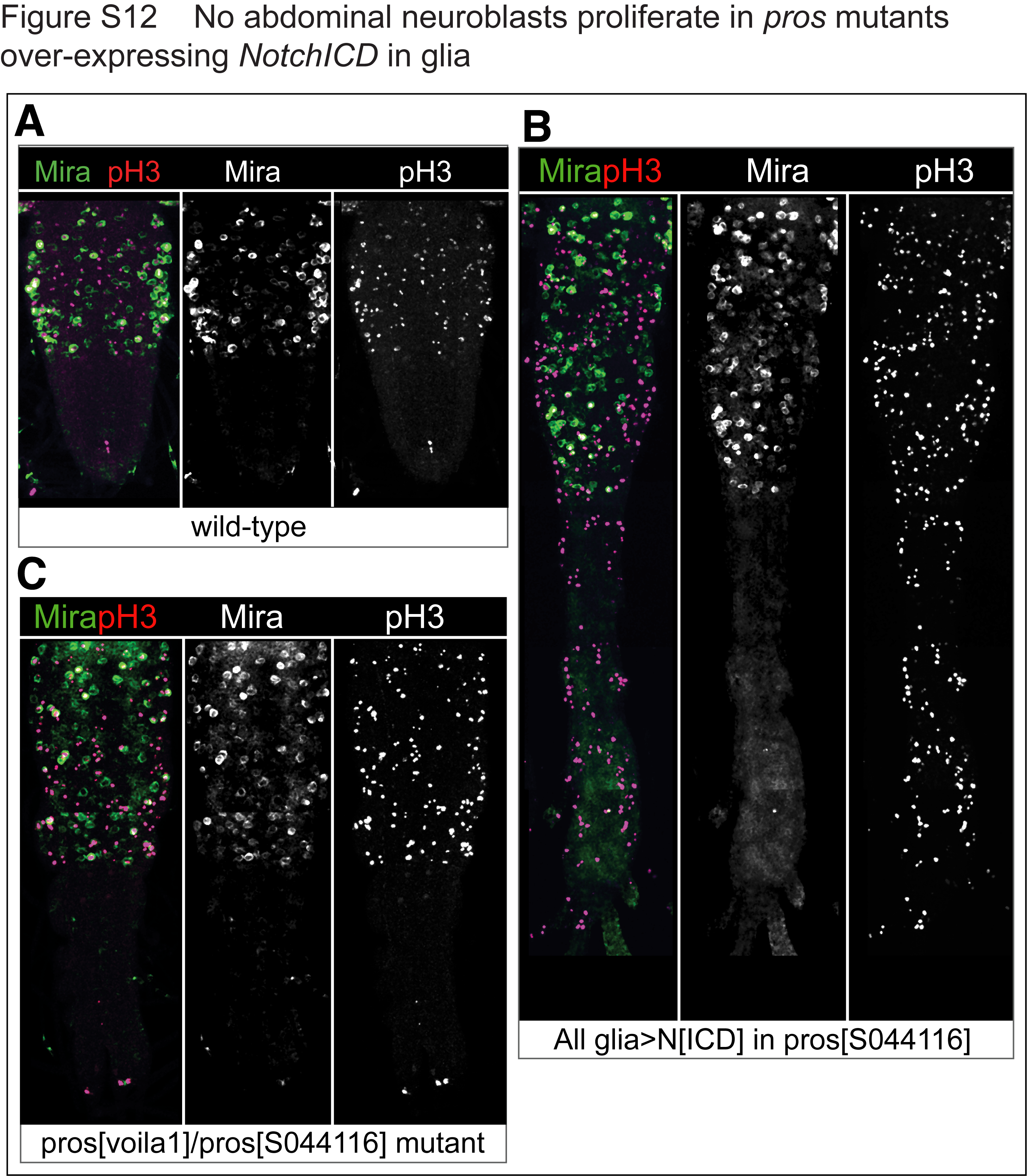

Supplement: Figure S12 — No abdominal neuroblasts proliferate in pros mutants over-expressing NotchICD in glia. (A, C) The colocalisation of the mitotic marker anti-phospho-Histone-H3 (pH3) and the neuroblast marker anti-Miranda (Mira) is restritcted to the thorax in wild-type (A) and pros- (C) mutant larvae. (B) There is no colocalisation of pH3 and Mira in the abdomen of pros mutant VNCs over-expressing NotchICD in glia. pH3+ and Mira-negative spots in the abdomen correspond to glia, as shown in Figure 5B of the main manuscript, as they colocalise with the glial marker Repo. Genotypes: (A) wild-type = yw; (B) prosS044116repoGAL4/prosS044116UASNotchICDmyc; (C) prosvoila1/prosS044116. (TIF) [file pbio.1001133.s012.tif]

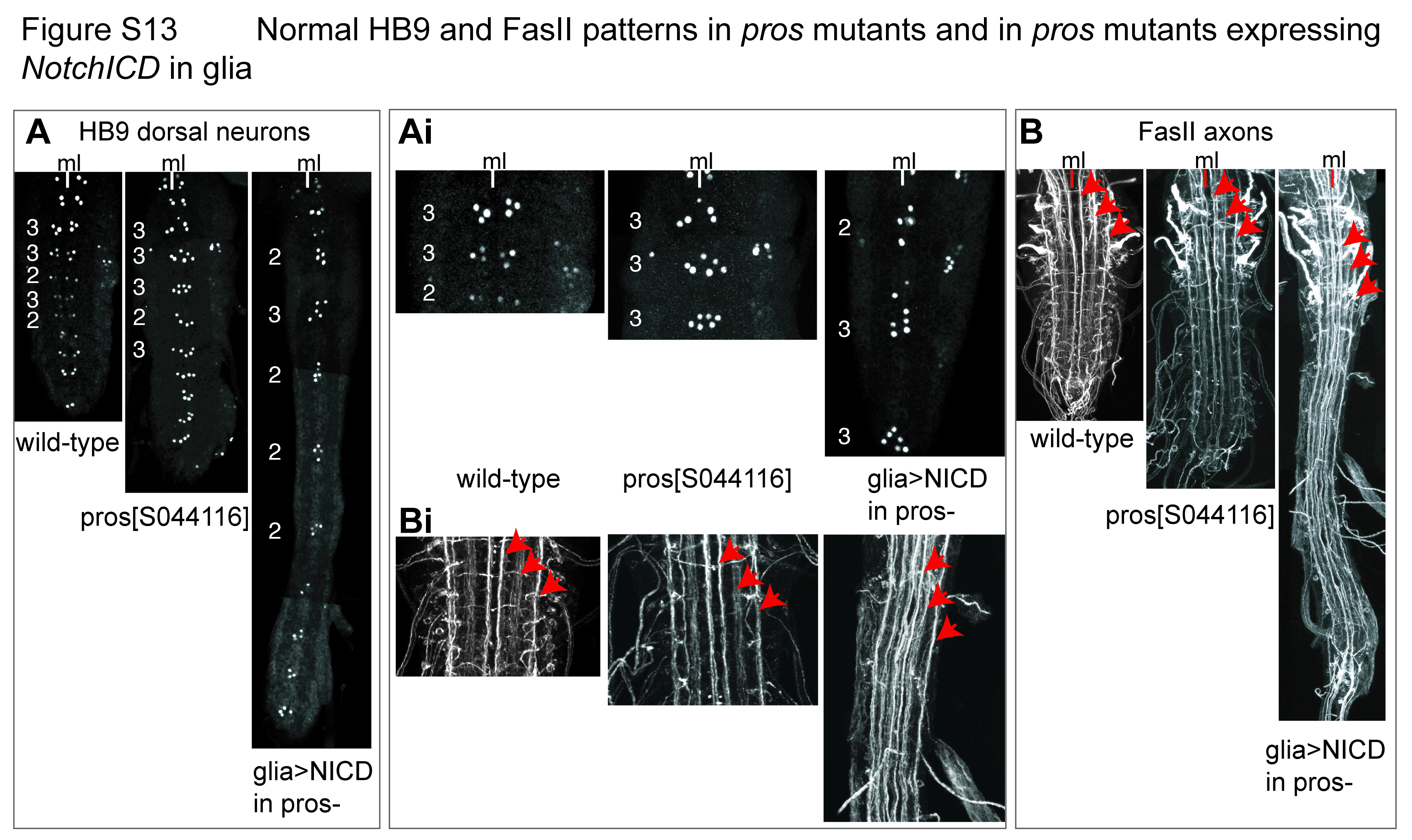

Supplement: Figure S13 — Normal HB9 and FasII patterns in pros mutants and in pros mutants expressing NotchICD in glia. Confocal longitudinal projections of larvae labelled with the neuronal markers (A,Ai) nuclear anti-HB9 and (B) axonal FasII. (A,Ai) The number of HB9 neurons is virtually the same in the three different genotypes. (B) The three major, dorsal FasII fascicles appear normal in the three different genotypes (arrowheads). These are VNC from wandering stage larvae. (Ai,Bi) are higher magnification detail of (A,B). Anterior is up; ml, midline. Genotypes: wild-type = yw; pros mutant: prosS044116/prosS044116; glia>NICD in pros- = prosS044116repoGAL4/UASNotchICDmyc prosS044116. (TIF) [file pbio.1001133.s013.tif]

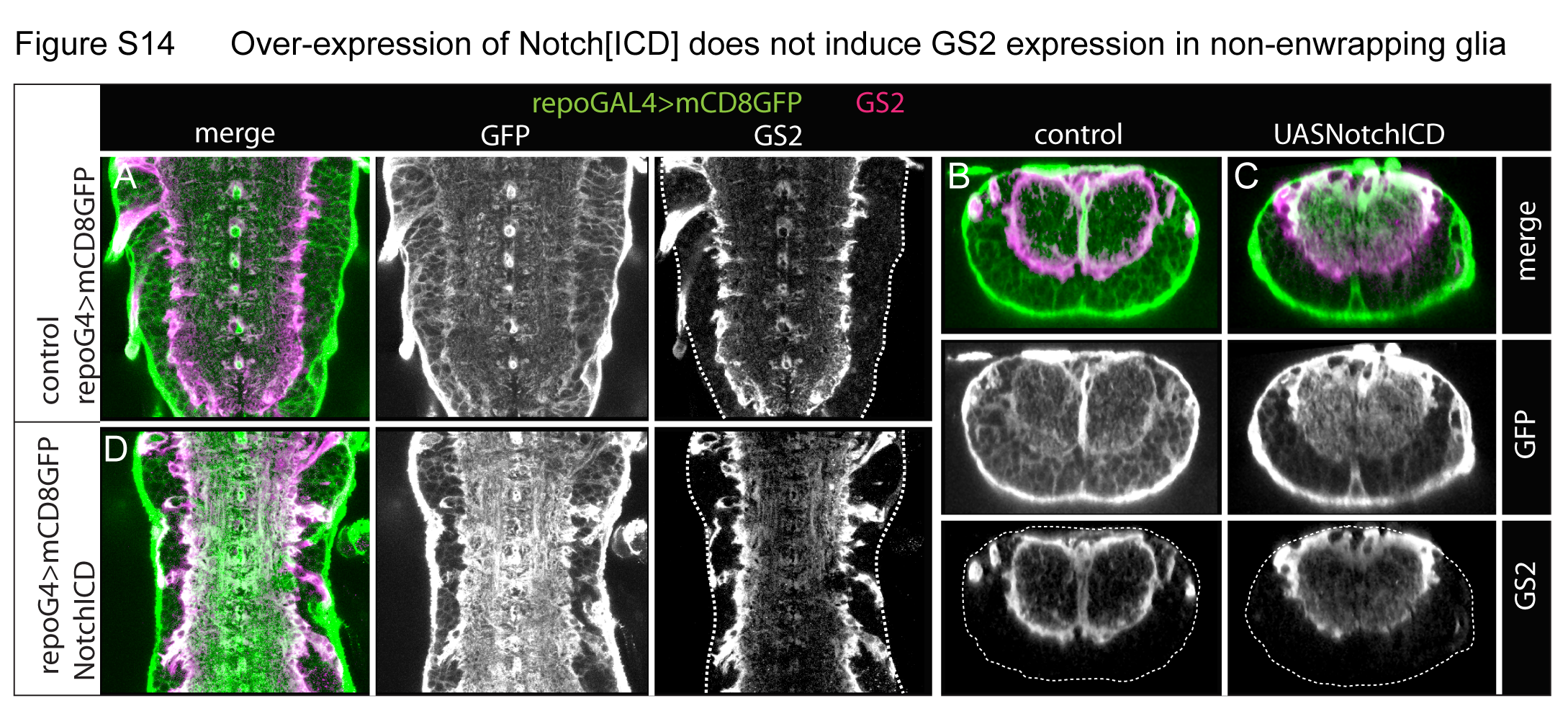

Supplement: Figure S14 — Over-expression of NotchICD does not induce GS2 expression in non-enwrapping glia. (A,D) Horizontal views of the abdominal VNC showing that GS2 expression is restricted to enwrapping glia and does not coincide with repoGAL4>mCD8GFP except for the neuropile and nerves exiting the CNS. (C,D) Transverse views showing that while GFP is present throughout the cortex, there is no GS2 expression outside the neuropile. Note also that NotchICD upregulates GFP predominantly within the neuropile. Genotypes: (A,B) Control: w; UASmCD8GFP/+; repoGAL4/+; (C,D) w; UASmCD8GFP/+; repoGAL4/UASNotchICDmyc. (TIF) [file pbio.1001133.s014.tif]

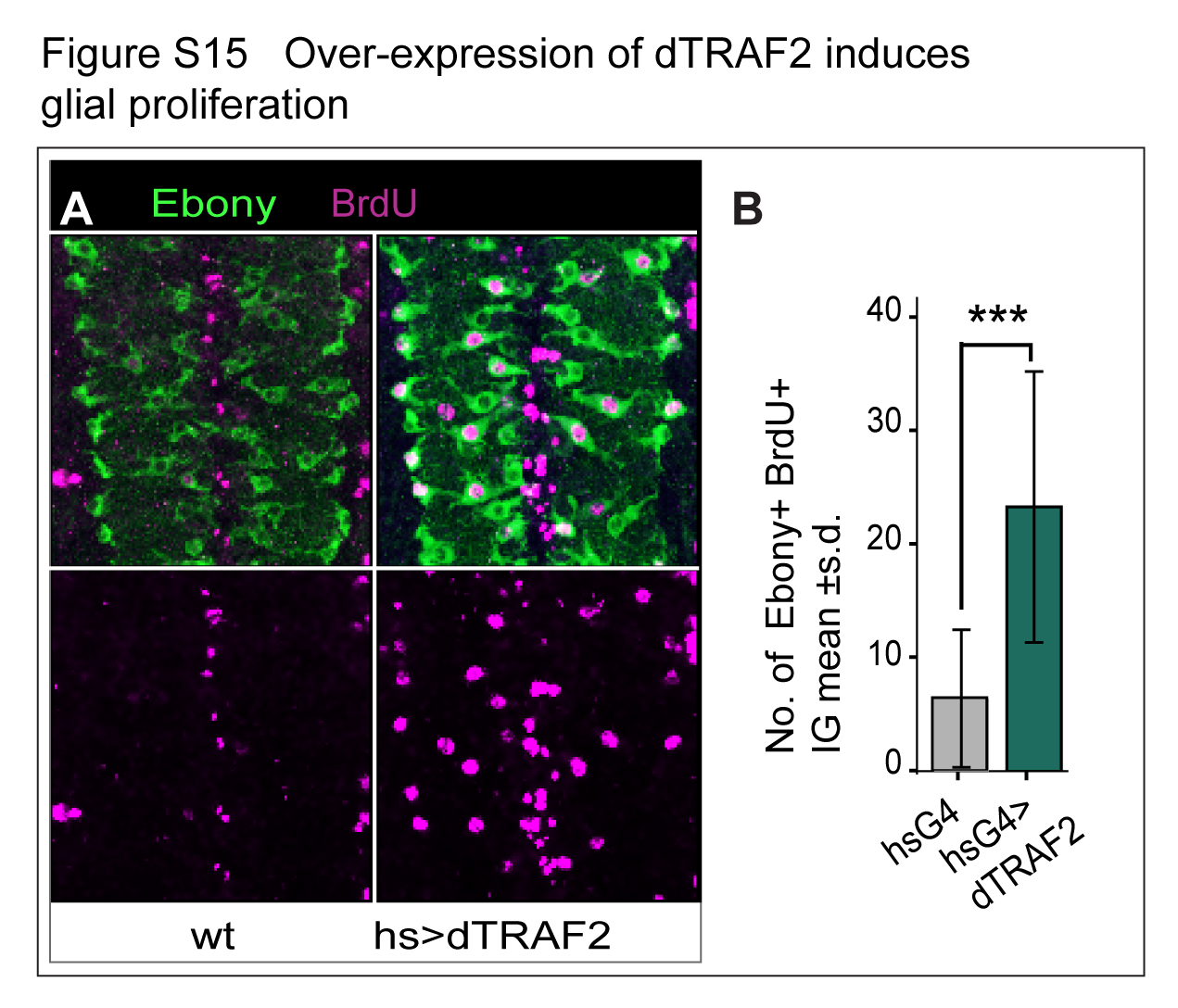

Supplement: Figure S15 — Over-expression of dTRAF2 induces glial proliferation. To test whether activation of Dorsal/NFkB can induce cell cycle progression, we induced a pulse of dTRAF2 expression at the mid-third instar stage using hsGAL4, during which larvae were fed with BrdU for a 6 h period. (A) Compared to controls, the number of Ebony+ IG cells incorporating BrdU increased significantly in larvae over-expressing dTRAF2, quantification in (B), showing that expression of dTRAF2 induces the G1-S transition in glia. Larvae were heat-shocked at 96 h AEL, treated with a BrdU pulse after 6 h, fixed 3 h later. Genotypes: (1) hsGAL4/+; (2) UASdTRAF2/+;;hsGAL4/+. (TIF) [file pbio.1001133.s015.tif]
