# Supplementary material for: The Glial Regenerative Response to Central Nervous System Injury Is Enabled by Pros-Notch and Pros-NFκB Feedback
Source: PLoS Biol. 2011 Aug 30;9(8):e1001133. doi: 10.1371/journal.pbio.1001133 (PMC3166069; doi:10.1371/journal.pbio.1001133)
Supplement: Table S1 — Statistical analysis: Sample sizes, tests and p values. (PDF) [file pbio.1001133.s016.pdf]

Table S1 Statistical analysis: sample sizes, tests and p values

Examination					
Multiple comparison before pair-wise comparison				TEST	p VALUE
TEST GENOTYPE		vs.	CONTROL GENOTYPE	TEST	p VALUE
**Number of abdominal PCNAGFP+ PROS+ EBONY+ IG - Injury**					**Figure 4C**
PCNAGFP	Stabbed (n=8)	vs.	not stabbed (n=9)	Mann-WhitenyU-test	p=0.000
**% hemisegments with >1 EBONY+ vIG in abdomen - Injury**					**Figure 4F**
yw	Stabbed (n=336)	vs.	not stabbed (n=320)	Fisher's exact test	p=0.047
**Number of abdominal REPO+ glia - Injury**					**Figure 4G**
yw injured, *yw* uninjured, *repoGAL4/UASrbf280* injured, *repoGAL4/UASrbf280* uninjured				Levene's test<br>Oneway ANOVA	p=0.371<br>p=0.000
yw	Stabbed (n=22)	vs.	not stabbed (n=20)	Student t-test	p=0.004
repoGAL4/UASrbf280	Stabbed (n=13)	vs.	not stabbed (n=15)	Student t-test	p=0.110
**Number of abdominal REPO+ glia -Injury**					**Figure 5A**
yw	Stabbed (n=22)	vs.	not stabbed (n=20)	Student t-test	p=0.004
repoGAL4/UASrbf280	Stabbed (n=15)	vs.	not stabbed (n=13)	Student t-test	p=0.110
repoGAL4/UASnumb	Stabbed (n=14)	vs.	not stabbed (n=20)	Student t-test	p=0.598
pros^{voila1}/*pros*^{S044116}	Stabbed (n=10)	vs.	not stabbed (n=12)	Student t-test	p=0.311
Df(1)Exel7463/pBacwgn^{e00637}	Stabbed (n=12)	vs.	not stabbed (n=9)	Student t-test	p=0.005
dorsal^{1}/*dorsal*^{H}	Stabbed (n=22)	vs.	not stabbed (n=18)	Student t-test	p=0.064
**Number of abdominal REPO+ glia -Wandering stage**					**Figure 6D**
yw, pros^{voila1}/*pros*^{S044116}*,repoGAL4/UASnumb, repoGAL4/UAS UASNotch*^{ICD} *repoGAL4/UASNotch*^{ICD}*UAScycE, pros*^{S044116} *repoGAL4/pros*^{S044116}*UAS UASNotch*^{ICD}				Levene's test<br>Kruskal-Wallis test	p=0.000<br>p=0.000
repoGAL4/ UASNotch^{ICD} (n=6)		vs.	*yw* (n=22)	Mann-Whiteny U-test	p=0.000
repoGAL4/ UASNotch^{ICD}*UAScycE* (n=5)		vs.	*repoGAL4/ UASNotch*^{ICD} (n=6)	Mann-Whiteny U-test	p=0.006
pros^{S044116}*repoGAL4/ pros*^{S044116} *UASNotch*^{ICD} (n=7)		vs.	*repoGAL4/ UASNotch*^{ICD} (n=6)	Mann-Whiteny U-test	p=0.032
**Number of abdominal EBONY+ IG - Wandering stage**					**Figure 6E**
repoGAL4/ UASNotch^{ICD} (n=4)		vs.	*yw* (n=6)	Mann-Whiteny U-test	p=0.003
**Number of mCD8GFP+ IG - Wandering stage**					**Figure 6F**
UASmCD8GFP/+;alrmGAL4/UASNotch^{ICD} (n=6)		vs.	*UASmCD8GFP/+;alrmGAL4/+* (n=7)	Student t-test	p=0.003
**Number of abdominal REPO+ glia –wandering stage**					**Figure 6G**
tubGAL80ts/UASpros;repoGAL4/+ (n=6)		vs.	*tubGAL80ts/+;repoGAL4/+* (n=6)	Student t-test	p=0.000
**Number of abdominal REPO+ glia - Wandering stage**					**Figure 8C**
yw, pros^{voila1}/*pros*^{S044116}, *eiger1, dorsal*^{1}/*dorsal*^{H}, *UASdTraf2/+;repoGAL4/+,UASdTraf2/+;dorsal*^{1}/*dorsal*^{H}*;repoGAL4/+, UASdTraf2/+; pros*^{S044116} *repoGAL4/ pros*^{S044116}				Levene's test<br>Oneway ANOVA	p=0.687<br>p=0.000
UASdTraf2/+;repoGAL4/+ (n=6)		vs.	*yw* (n=22)	Student t-test	p=0.000
UASdTraf2/+;pros^{S044116}*repoGAL4/pros*^{S044116} (n=6)		vs.	*UASdTraf2/+;repoGAL4/+* (n=6)	Student t-test	p=0.000
UASdTraf2/+; dorsal^{1}/*dorsal*^{H}*;repoGAL4/+* (n=6)		vs.	*UASdTraf2/+;repoGAL4/+* (n=6)	Student t-test	p=0.003
**Area occupied by glial wound (measured on EBONY) -Injury**					**Figure 9C**
egr^{1}*;pros*^{voila1}/*pros*^{S044116} (n=10)		vs.	*yw* (n=22)	Student t-test	p=0.019
**Area occupied by glial wound (measured on GS2) -Injury**					**Figure 9D**
yw, repoGAL4/UASNotch^{ICD}, *pros*^{S044116} *repoGAL4/pros*^{S044116}*UAS UASNotch*^{ICD}				Levene's test<br>Kruskal-Wallis test	p=0.001<br>P=0.001
repoGAL4/UASNotch^{ICD} (n=12)		vs.	*yw* (n=12)	Mann-Whiteny U-test	p=0.000
pros^{S044116}*repoGAL4/pros*^{S044116}*UASNotch*^{ICD} (n=7)		vs.	*repoGAL4/UASNotch*^{ICD} (n=12)	Mann-Whiteny U-test	p=0.017
**Number of abdominal ACTIVE CASPASE 3+ cells -Injury**					**Figure 9E**
yw injured, *yw* uninjured *repoGAL4/UASNotch*^{ICD} stabbed, *repoGAL4/UASNotch*^{ICD} not stabbed,				Levene's test<br>Oneway ANOVA	p=0.332<br>p=0.000
yw	Stabbed (n=8)	vs.	not stabbed (n=8)	Student t-test	p=0.000
repoGAL4/UASNotch^{ICD}	stabbed (n=8)	vs.	*yw* stabbed (n=7)	Student t-test	p=0.002

Test genotype		Control genotype	Test	p-value
**% of abdominal BrdU+ Ebony+ over Ebony+ -Injury**				**MS**
yw Stabbed (n=10)	vs.	not stabbed (n=9)	Mann-Whiteny U-test	p=0.034
**Number of abdominal histoneYFP+ IG -Injury**				**MS**
alrmGAL4/UAShistoneYFP ;$pros^{S044116}$/$pros^{S044116}$ Stabbed (68.8, n=13)	vs.	not stabbed (81.8, n=8)	Student t-test	p=0.007
**Number of intense DORSAL+ EBONY+ IG -Injury**				**MS**
yw Stabbed (50%, n=10)	vs.	not stabbed (0%, n=9)	Fisher's Exact test	p=0.033
egr^{1} Stabbed (0%, n=10)	vs.	*yw* Stabbed (50%, n=10)	Fisher's Exact test	p=0.033
**Number of DAP+ EBONY+ IG in a segment**				**MS**
tubGAL80ts/UASpros;repoGAL4/+ (n=10)	vs.	*tubGAL80ts/+;repoGAL4/+* (n=6)	Mann-Whiteny U-test	p=0.016
**Number of abdominal EBONY+ IG - Wandering stage**				**MS**
UASdTraf2/+;repoGAL4/+ (87.1, n=7)	vs.	*yw* (84.1, n=12)	Student t-test	p=0.001
**Number of abdominal EBONY+ IG - Wandering stage**				**Text S1**
tubGAL80ts/+; repoGAL4/UAScycE (89.9, n=7)	vs.	*yw* (81.3, n=4)	Student t-test	P=0.006
**Number of abdominal EBONY+ BrdU+ IG**				**Supplementary Figure 3D**
tubGAL80ts/+; repoGAL4/UAScycE (n=13)	vs.	*yw* (n=12)	Student t-test	p=0.001
**Number of abdominal REPO+ glia -96h AEL**				**Supplementary Figure 10A**
$pros^{voila1}$/$pros^{S04411}$ (n=6)	vs.	yw (n=7)	Student t-test	p=0.000
**Number of abdominal REPO+ glia -Wandering stage**				**Supplementary Figure 10A**
$pros^{voila1}$/$pros^{S044116}$ (n=22)	vs.	*yw* (n=22)	Student t-test	p=0.542
**Number of abdominal REPO+ glia - Culture**				**Supplementary Figure 10B**
yw, $pros^{voila1}$/$pros^{S044116}$, $pros^{S044116}$ repoGAL4 /$pros^{S044116}$UASrbf280			Levene's test	p=0.004
			Kruskal-Wallis test	p=0.000
$pros^{voila1}$/$pros^{S044116}$ (n=12)	vs.	yw (n=20)	Mann-Whiteny U-test	p=0.000
$pros^{S044116}$repoGAL4/$pros^{S044116}$UASrbf280 (n=13)	vs.	*$pros^{voila1}$/$pros^{S044116}$* (n=12)	Mann-Whiteny U-test	p=0.000
**Number of PCNAGFP+ enwrapping glia in a segment**				**Supplementary Figure 10E**
PCNAGFP/+;;hsGAL4/UAS$Notch^{ICD}$ heatshocked (n=8)	vs.	not heatshocked (n=11)	Student t-test	p=0.000
**Number of abdominal EBONY + BrdU IG**				**Supplementary Figure 10G**
hsGAL4/UAS$Notch^{ICD}$ (n=10)	vs.	*hsGAL4/+* (n=7)	Student t-test	p=0.014
**Number of abdominal EBONY+ IG - Wandering stage**				**Supplementary Figure 11F**
yw, $pros^{voila1}$/$pros^{S044116}$, repoGAL4/UASnumb, repoGAL4/UAS$Notch^{ICD}$			Levene's test	p=0.000
			Kruskal-Wallis test	p=0.000
$pros^{voila1}$/$pros^{S044116}$ (n=4)	vs.	*yw* (n=12)	Mann-Whiteny U-test	p=0.003
repoGAL4/ UAS$Notch^{ICD}$ (n=4)	vs.	*yw* (n=6)	Mann-Whiteny U-test	p=0.003
**Number of abdominal EBONY + BrdU IG**				**Supplementary Figure 15B**
UASdTraf2/+;;hsGAL4/+ (n=9)	vs.	*hsGAL4/+* (n=7)	Student t-test	p=0.000

White writing on black background indicates experiment; black writing on grey background indicates tests applied to multiple genotypes within one experiment; black on white background is for pair-wise comparisons. Test genotypes are on the left, compared to the control genotypes on the right. For multiple comparisons, One Way Anova and Kruskal-Wallis tests were used; for pair-wise comparisons, Fisher’s exact, Mann-Whitney-U and Student-t tests were applied. For sample sizes, see figures.
