# Supplementary material for: The Glial Regenerative Response to Central Nervous System Injury Is Enabled by Pros-Notch and Pros-NFκB Feedback
Source: PLoS Biol. 2011 Aug 30;9(8):e1001133. doi: 10.1371/journal.pbio.1001133 (PMC3166069; doi:10.1371/journal.pbio.1001133)
Supplement: Text S1 — Evidence that IG do not normally divide but are arrested in G1 and can be induced to divide. (DOC) [file pbio.1001133.s017.doc]

**SUPPORTING INFORMATION**

**The Glial Regenerative Response to Central Nervous System Injury Is Enabled by Pros-Notch and Pros-NFκB Feedback**

Kentaro Kato^1^, Manuel G. Forero^1^, Janine C. Fenton^1,2^, Stephanie Fennell^1^ and Alicia Hidalgo^1^*

**TEXT S1 Evidence that IG do not normally divide but are arrested in G1 and can be induced to divide**

Labelling normal, non-stabbed larvae with the mitotic marker anti-phospho-Histone H3 (pH3) revealed cell divisions in cortex and surface glia (data not shown), but rarely in IG, consistently with a previous report [1]. Only 0.6% VNCs had one pH3+ IG at the mid-third instar stage (n=181 VNCs Figure S3A). Furthermore, only 1.2% VNCs had one GFP-labelled mitotic recombination MARCM clone of IG (n=1254 VNCs, Figure S3B,Bi). These data show that IG do not normally divide.

Cells that are not actively proliferating may be arrested in the G1 or G2 phases of the cell cycle, or may have terminally exited the cell cycle (G0). Cells in G1/G2 have mitotic potential, whereas cells in G0 do not: cells in G1 can enter S-phase immediately after increasing Cyclin E (CycE) levels; cells in G0 either do not enter the cell cycle again even in the presence of CycE, or may take considerably longer to do so [2]. To find out whether IG are in G1 or G0, we transiently over-expressed *cycE* and asked if this pushed IG into S-phase. To induce the transient expression of *cycE* in glia at the mid-third instar larval stage, we made use of the GAL4 repressor GAL80^ts^, which functions at 18°C but not at 30°C. A pulse of *cycE* over-expression was induced by shifting *tubGAL80^ts^;repoGAL4*/*UAScycE* larvae to 30°C at the mid-third instar stage, and fixing larvae 6 hours later. This led to an increase in the number of IG that incorporated BrdU compared to controls (Figure S3C,D). Sustained expression of *cycE* from the mid-third instar larva to the wandering stage increased the total number of IG compared to wild-type, implying that more cells completed cell division (p<0.01). These experiments reveal that in the normal, non-injured larva, generally IG do not divide. However, IG do not terminally exit the cell cycle but are maintained in G1 arrest, ready to divide.

REFERENCES

1. Prokop A, Technau GM (1994) BrdU incorporation reveals DNA replication in non dividing glial cells in the larval abdominal CNS of Drosophila. Roux's Arch Dev Biol 204: 54-61.

2. Griffiths RL, Hidalgo A (2004) Prospero maintains the mitotic potential of glial precursors enabling them to respond to neurons. EMBO J 23: 2440-2450.
