# Supplementary material for: The Glial Regenerative Response to Central Nervous System Injury Is Enabled by Pros-Notch and Pros-NFκB Feedback
Source: PLoS Biol. 2011 Aug 30;9(8):e1001133. doi: 10.1371/journal.pbio.1001133 (PMC3166069; doi:10.1371/journal.pbio.1001133)
Supplement: Text S2 — Evidence that the increase in glial number is not due to neuroblast divisions. (DOC) [file pbio.1001133.s018.doc]

**SUPPORTING INFORMATION**

**The Glial Regenerative Response to Central Nervous System Injury Is Enabled by Pros-Notch and Pros-NFκB Feedback**

Kentaro Kato^1^, Manuel G. Forero^1^, Janine C. Fenton^1,2^, Stephanie Fennell^1^ and Alicia Hidalgo^1^*

**TEXT S2 Evidence that the increase in glial number is not due to neuroblast divisions**

To test whether the dramatic increase in glial number and VNC size were due to direct, cell-autonomous functions of Pros and Notch in glia and not to non-autonomous effects on neuroblast proliferation, we analysed the distribution of the neuroblast marker Miranda (Mira) and the mitotic marker pH3 in *pros* mutants expressing *Notch^ICD^* in glia (Figure S12A-C). There was no ectopic distribution of Mira+ cells, and no co-localisation of Mira and pH3, in the abdominal VNC. The distribution of HB9 and FasII positive neurons was also unaltered (Figure S13A,Ai,B,Bi). These data show that the increase in abdominal VNC size in *repoGAL4 pros^S044116^/UASNotch^ICD^pros^S044116^* larvae was not due to a non-autonomous excess of neuroblast proliferation nor an increase in the number of differentiated neurons.
