# Supplementary material for: The Glial Regenerative Response to Central Nervous System Injury Is Enabled by Pros-Notch and Pros-NFκB Feedback
Source: PLoS Biol. 2011 Aug 30;9(8):e1001133. doi: 10.1371/journal.pbio.1001133 (PMC3166069; doi:10.1371/journal.pbio.1001133)
Supplement: Text S3 — Detailed methods. (DOC) [file pbio.1001133.s019.doc]

**SUPPORTING INFORMATION**

**The Glial Regenerative Response to Central Nervous System Injury Is Enabled by Pros-Notch and Pros-NFκB Feedback**

Kentaro Kato^1^, Manuel G. Forero^1^, Janine C. Fenton^1,2^, Stephanie Fennell^1^ and Alicia Hidalgo^1^*

**TEXT S3: DETAILED METHODS**

**Genetics**

Experiments were carried out in mid-third instar larvae at 96h after egg laying (AEL) or at the wandering stage (120h AEL at 25°C in wild-type) from 3h egg laying collections raised at 25°C, unless otherwise indicated. Stocks of the following genotypes were used, and stocks bearing combinations of mutants, reporters, GAL4 or UAS were generated by conventional genetics: **Wild-type**, *yw*. **Reporter lines**, (1) *pros-lacZ;* (2) *p12xSu(H)bs-lacZ*(gift of M.Okabe)[1], which contains 12 binding sites for Su(H) upstream of lacZ; (3) *PCNA-GFP,* a protein fusion of GFP to PCNA expressed under the control of E2F binding sites (gift of Matthew Freeman)[2]; (4) *G9*, a protein-trap line that drives expression of GFP in all axons (gift of W. Chia)[3]. **Mutants,** (1) *pros^S044116^*/*TM6B* (Szeged) and *pros^voila1^*/*TM6B* (gift of J.F.Ferveur)[4] for trans-heterozygous *pros* mutant larvae; and *p12xSu(H)bs-lacZ; pros^S044116^*/*TM6B* and *p12xSu(H)bs-lacZ; pros^voila1^*/*TM6B* for trans-heterozygous *pros* mutant bearing the Su(H)lacZ reporter. (2) *dorsal^1^ cn^1^ sca^1^*/*CyOactGFP* (Bloomington) and *dorsal^H^*/*CyOactGFP* (gift of T.Ip) for trans-heterozygous *dorsal* mutant larvae; (3) *w; eiger^1^* and *w; eiger^3^*  for trans-heterozygous *eiger* mutants (gift of M.Miura*)*[5];(4) *wgn^e00637^w a*nd *Df(1)Exel7463 w* /*FM7actGFP* (Bloomington) for trans-heterozygous *wengen* mutant larvae; (5) *eiger^1^*; *pros^S044116^/TM6B* flies and *eiger^1^*; *pros^voila1^*/*TM6B* for double mutants of *egr* and *pros*. (6) *Notch^ts1^/FM7(sn+)actGFP* (Bloomington) is a temperature sensitive allele of Notch which is a loss of function mutant at 30°C (restrictive temperature) but not at 18°C (permissive temperature). **Over-expression** experiments were performed by crossing either *w; repoGAL4*/*TM6B* or w; *alrmGAL4* (gift of Marc Freeman)[6] flies to flies with the following UAS transgenes: (1) *w; UASNotch^ICD^myc*/*TM6B* (gift of A.Martinez-Arias); (2) *w; UASnumb/TM6B* (gift of A.Martinez-Arias); (3) *w, EP1516* (*UASdTraf2)*(Bloomington)[7,8]; (4) *w; UASrbf280*/*TM6B* (gift of B.Edgar)[9]; (7) *w; UASNotch^ICD^ myc* *UAScycE*/*TM6B*[10]; (8) *w;UASmCD8GFP*; (9) *w; UASpros* (gift of F.Matsuzaki); (10) *UASdsRed-S197Y*; (11) *w;UAShistoneYFP.* **Rescue experiment with dTraf2 over-expression** was done by crossing *dorsal^1^ cn^1^ sca^1^*/*CyOactGFP; repoGAL4/TM6B* to *w EP1516/y; dorsal^H^*/*CyOactGFP***. Over-expression in *pros* mutant background** was carried out crossing *pros^S044116^repoGAL4*/*TM6B* flies to: (1) *pros^S044116^UASrbf280*/*TM6B*; (2) *pros^S044116^UASNotch^ICD^ myc/TM6B;* (3) *w EP1516*; *pros^S044116^*/*TM6B*, and also by crossing *w;alrmGAL4;pros^S044116^/TM6B* flies to *w;UAShistoneYFP*; *pros^S044116^/TM6B.* **Cell morphology of IG** was visualised with *mCD8GFP* expressed under the control of the IG specific driver *alrmGAL4* (gift of M. Freeman)[6] in progeny larvae of the following crosses: (1) w; *alrmGAL4* crossed to *w;UASmCD8GFP*; (2) *UASmCD8GFP;pros^S044116^*/TM6B to *alrmGAL4;pros^S044116^*/TM6B; (3) *w; UASpros* to *w;UASmCD8GFP;alrmGAL4;* (4) *Notch^ts1^, UASDsRed S197Y /FM7(sn+)actGFP* *to w;UASmCD8GFP;alrmGAL4*; (5) *w; UASNotch^ICD^ myc* to *w;UASmCD8GFP;alrmGAL4*. In all cases, homozygous and trans-heterozygous mutant larvae and other experimental genotypes were identified by the lack of *TM6B* (as *Tb-)* or *CyOactGFP* (as *GFP-)* or *FM7(sn+)actGFP* balancers.

**Temporal control** **of gene expression in larval stages only**

To drive gene expression in larval stages only, thus enabling normal embryogenesis, we used the temperature sensitive GAL4 repressor GAL80^ts^ - the activity of which is inhibited at 30°C - driven by the general *tubulin* promoter in *tubGAL80^ts^* flies (Bloomington)[11], or *hsGAL4* (gift of S.Brogna) flies where GAL4 is switched on after heat-shock at 37°C. *tubGAL80^ts^*; *repoGAL4* flies were crossed with flies bearing: (1) w;*UAScycE* (gift of C.Lehner); (2) *w; UASNotch^ICD^ myc*/*TM6B;* (3) w;*UASPros* (gift of F.Matsusaki). For over-expression of *cycE* in larval glia, mid-third instar larvae (8 days AEL at 18°C) were placed at 30°C for 6 hours for the BrdU (1mg/ml) incorporation experiment (Figure S3C), and for 24 hours to count Ebony glia (Table S1). For a pulse of over-expression of *pros* (Figure S10C), larvae at mid-third instar stage were heat-shocked for 1 hour at 37°C and kept at 30°C for another 7 hours. For larval over-expression of *pros* (Figure 6G) first instar larvae (2 days after egg laying – AEL - at 18°C) were shifted to 30°C, and fixed when control larvae *(tubG80ts/+;repoGAL4/+)* reared in the same conditions wandered. *hsGAL4* flies were crossed to flies bearing: (1) *PCNA-GFP*; *UASNotch^ICD^* myc; (2) *UASNotch^ICD^ myc;* and (3) *w; EP1516(UASdTraf2)*. Mid-third instar larvae raised at 18°C were heat-shocked at 37°C for 30 minutes and kept at 25°C. For the experiments with *PCNA-GFP*; *UASNotch^ICD^myc* (Figure S10D,E), larval CNSs were fixed after 9 hours. For the experiments with EP1516 and *UASNotch^ICD^* myc, larvae were fed with BrdU (1mg/ml) containing food for 3 hours after 6 hours of the heat-shock (Figure S10F,G and Figure S14A,B). Control flies were of the same genotypes but not heat-shocked or heat-shocked *hsGAL4/+*. **To knock down Notch function during larval stages only**, temperature sensitive *Notch^ts1^* embryos were kept at the permissive temperature (18°), and were shifted to the restrictive temperature (30°) at hatching (first instar) and kept at this temperature until early to mid third instar stage when experiments were performed.

**MARCM clones**

Eggs were collected for 6 hours and kept at 18°C until heat-shock was applied to the larvae. Mosaic Analysis with a Repressible Cell Marker (MARCM)[12] clones were generated after crossing: (1) For wild-type clones: *hsFLP tubP-GAL80* *FRT19A*; *repoGAL4*/*TM3Ser* to *FRT19A*; *UASmCD8GFP*. (2) For *pros* mutant clones: *hsFLP*; *neoFRT82B* *tubP-GAL80* to *actGAL4 UASGAPGFP*; *neoFRT82B* *pros^JO13^* *F263*/*TM6B*. Early to mid-third instar larvae were heat-shocked at 37°C for 1 hour and kept at 25°C until the wandering stage.

**Stabbing injury to the larval ventral nerve cord**

This has been fully described in the main manuscript.

**Immunohistochemistry, in situ hybridization and BrdU incorporation.**

Larval VNCs were dissected in PBS and fixed with 4% Formaldehyde PEM (100 mM PIPES, 2 mM EGTA, 1 mM MgSO_4_, pH 7) for 50 min at room temperature (RT). After washing with 0.3% TritonX PBS, brains were treated with 0.5% TritonX PBS for 30 minutes at RT. Blocking was in 10% normal goat serum (Vector Laboratories) 0.3% TritonX PBS for 1 hour, and incubation with primary and fluorescent-conjugated secondary antibodies was performed at 4°C overnight. For BrdU detection, the VNCs were treated with 2M HCl for 20 min at room temperature after immunolabeling for other proteins. Samples were mounted either in Vectashield with DAPI (Vector Laboratories) or in 80% Glycerol PBS after staining nuclei with Daunomycin 5μg/ml (Sigma) or Hoechst33342 5µg/ml (Sigma).

Primary antibodies were at the following dilutions: mouse anti-REPO 1:250 (DSHB); (2) rabbit anti-Ebony 1:250 (gift of S.Carroll)[13]; (3) mouse anti-Glutamine Synthetase 1:250 (Millipore); (4) mouse anti-Pros 1:250 (DSHB); (5) mouse anti-Serrate 1:250 (gift of K.Irvine and DSHB); (6) rat anti-Elav 1:250 (DSHB); (7) mouse anti-Dorsal 1:100 (gift of D.Ferrandon and DSHB); (8) mouse anti-Dacapo 1:100 (gift of H.Vaessin); (9) rabbit anti-cleaved Caspase-3 1:100 (Cell Signalling Technology) and 1:1000 (abCam); (10) chick anti-β-galactosidase 1:1000 (Kamiya Biomedical); (11) rabbit anti-β-galactosidase 1:1000 (Cappel); (12) rabbit anti-GFP 1:1000 (Molecular Probes); (13) chick anti-GFP 1:1000 (Millipore); (14) mouse anti-BrdU 1:100 (GE Healthcare); (15) mouse anti-FasII ID4 1:250 (DSHB); (16) Guinea pig anti-HB9 1:2500 (gift of H. Broihier); (17) mouse anti-Miranda 1:10 (gift of W.Chia); (18) mouse anti-BP102 1:100 (DSHB); (19) rabbit anti-pHistone-H3 (Upstate Biotechnolgy). Alexa Fluor 488, 546, 660 or 647-conjugated secondary antibodies (Molecular Probes) were used.

In situ hybridisations to mRNA were performed with DIG-labelled antisense RNA probes following standard methods (Ambion kit) except that VNCs were fixed with 4% para-formaldehyde in PEM for 50 minutes at RT°C. After Proteinase-K treatment, VNCs were hybridised. DIG-RNA probes for *eiger* were made from plasmid SD18286 BDGP EST (DGRC), linearised with Eco RV and transcribed with SP6 RNA polymerase; *wengen* DIG-RNA probes were made from plasmid RE29502 BDGP EST (DGRC) linearising with SacI and transcribed with T3 RNA polymerase.

BrdU incorporation experiments were carried out by culturing non-stabbed or stabbed dissected VNCs in culture medium containing 0.3µg/ml BrdU (Sigma) for 6 hours (after stabbing) (Figure S4); or feeding larvae with yeast paste containing 1mg/ml BrdU after the shift to non-permissive temperature (Figure S3C, Figure S10D,F and Figure S14A).

**Microscopy**

Images from VNCs stained by immunohistochemistry were acquired using laser scanning confocal microscopy in the following ways: 1) anti-Repo (for automatic quantification using DeadEasy Larval Glia) and anti-Ebony stained samples were scanned using a BioRad Radiance 2000 confocal microscope. 2) All other samples were scanned using a Leica SP2-AOBS confocal microscope. Bright-field microscopy was carried out using a Zeiss Axioplan 2 microscope, JVC camera and Neotec Image Grabber card. The Z-projections of confocal sequential images were generated with ImageJ, and transverse sections and their projections were generated using Zeiss Image Browser or Volocity (Improvision) software. Image J, Adobe Photoshop and Adobe Illustrator were used for image processing.

To examine the morphology of IG in multiple genotypes by Transmission Electron Microscopy, CNS from wandering larvae were dissected in cold PBS and fixed with primary fixative (2.5% glutaraldehyde in 0.05M phosphate buffer, pH7.3) at 4°C overnight. To examine the morphology of IG in stabbed specimens, CNS were dissected, stabbed and cultured for 6 hours as described above, and fixed with primary fixative as above. The samples were postfixed with 1% osmium tetroxide in 0.05M phosphate buffer for 1hour. Subsequently the samples were washed, dehydrated and embedded in an Araldite/Epon resin. After sectioning with 70-90nm steps and mounting on grids, the sections were stained with 30% Methanolic Uranyl Acetate for 7 minutes, and then with Reynolds Lead Citrates for 7 minutes. Transmission electron microscopy was carried out using JEOL 1200EX, and images were recorded with a Gatan digital camera. Image montages were generated using Adobe photoshop.

**Time-lapse confocal microscopy recordings**

This has been fully described in the main manuscript text.

**DeadEasy for automatic counting of glial and apoptotic cells, and other quantifications**

This has been fully described in the main manuscript text.

**Statistical analyses**

Statistical tests carried out for as shown in figures and in the text, sample sizes, tests applied and p values are given in Table S1. In all cases confidence interval was 95%. Our null hypotheses were that the experimental number of cells in each case did not differ from the controls, except for Figure 9C,D where the null hypothesis was that the area of the wound in experimental VNCs did not differ from the control. Statistical analyses were carried using SPSS windows v.15. When sample distribution was normal (or could be assumed to be normal), and variances were not significantly different according to Levene’s test, across the whole range of genotypes to be tested, we applied the One-way ANOVA test to compare means. Pair-wise comparisons of two genotypes were carried out for the most relevant genotypes only and in cases where there were only two genotypes, using the Student t-test. These data were represented as bar charts showing the mean ± standard deviation.

For experiments where sample distributions were not normal, the non-parametric Kruskal-Wallis test was applied for multiple comparisons and the Mann-Whitney U-test for pair-wise comparisons. These data are represented with box-plots, where the line across the box is the median, the box represents the distribution of 25% samples above and 25% samples below the median, and the whiskers represent the 25% quartiles furthest away from the median.

To quantify the effect of injury on the number of Repo-positive glial cells (Figure 5A), we normalised each genotype relative to the non-stabbed controls of the respective genotypes. The data are represented as percentage change in the number of Repo-positive cells: (number of Repo glia in stabbed/non-stabbed-1)x100, i.e. increases in glial number are positive values and decreases in glial number are negative values. The mean number of Repo-positive cells in non-stabbed and stabbed samples of the same genotype were compared using Student’s t-test.

For categorical data (Figure 4F), the Fisher’s exact test was performed to ask whether there was a difference in the frequency of having more than one vIG in a hemisegment between two populations .

**REFERENCES**

1. Go MJ, Eastman DS, Artavanis-Tsakonas S (1998) Cell proliferation control by Notch signaling in Drosophila development. Development 125: 2031-2040.

2. Thacker SA, Bonnette PC, Duronio RJ (2003) The contribution of E2F-regulated transcription to Drosophila PCNA gene function. Curr Biol 13: 53-58.

3. Morin X, Daneman R, Zavortnik M, Chia W (2001) A protein trap strategy to detect GFP-tagged proteins expressed from their endogenous loci in Drosophila. PNAS 98: 15050-15055.

4. Guenin L, Grosjean Y, Fraichard S, Acebes A, Baba-Aissa F, et al. (2007) Spatio-temporal expression of Prospero is finely tuned to allow the correct development and function of the nervous system in Drosophila melanogaster. Dev Biol 304: 62-74.

5. Igaki T, Kanda H, Yamamoto-Goto Y, Kanuka G, Kuranaga E, et al. (2002) Eiger, a TNF superfamily liogand that triggers the Drosophila JNK pathway. EMBO J 21: 3009-3018.

6. Doherty J, Logan MA, Tasdemir OE, Freeman MR (2009) Ensheathing glia function as phagocytes in the adult Drosophila brain. J Neurosci 29: 4768-4781.

7. Cha G-H, Cho KS, Lee JH, Kim M, Park J, et al. (2003) Discrete functions of TRAF1 asnd TRAF2 in Drosophila melanogaster mediated by c-Jun N-terminal kinase and NFkB-dependent signaling pathways. Molecular and Cellular Biology 23: 7982-7991.

8. Rorth P (1996) A modular misexpression screen in Drosophila detecting tissue-specific phenotypes. Proc Natl Acad Sci U S A 93: 12418-12422.

9. Xin S, Weng L, Xu J, Du W (2002) The role of RBF in developmentally regulated cell proliferation in the eye disc and in Cyclin D/Cdk4 induced cellular growth. Development 129: 1345-1356.

10. Griffiths RL, Hidalgo A (2004) Prospero maintains the mitotic potential of glial precursors enabling them to respond to neurons. EMBO J 23: 2440-2450.

11. McGuire SE, Le PT, Osborn AJ, Matsumoto K, Davis RL (2003) Spatiotemporal rescue of memory dysfunction in Drosophila. Science 302: 1765-1768.

12. Lee T, Luo L (1999) Mosaic analysis with a repressible cell marker for studies of gene function in neuronal morphogenesis. Neuron 22: 451-461.

13. Suh J, Jackson FR (2007) Drosophila ebony activity is required in glia for the circadian regulation of locomotor activity. Neuron 55: 435-447.
